# Supplementary material for: First-trimester exposure to macrolides and risk of major congenital malformations compared with amoxicillin: A French nationwide cohort study
Source: PLoS Med. 2025 Apr 15;22(4):e1004576. doi: 10.1371/journal.pmed.1004576 (PMC12021278; doi:10.1371/journal.pmed.1004576)
Supplement: S1 Text — (DOCX) [file pmed.1004576.s001.docx]

**Text S1.**  Presentation of the databases in the SNDS

The SNDS is a repository of medico-administrative data that encompasses reimbursed healthcare services for all French healthcare insurance system beneficiaries. Consequently, the SNDS includes health reimbursement data for approximately 67 million residents (99% of the French population), making it one of the world’s largest homogeneous claims databases. The primary components of the SNDS include the Inter-Scheme Consumption Data (DCIR), the Program for the Medicalization of Information Systems (PMSI), the registry of beneficiaries from Medical-Social Care Facilities (R-ESMS), and the National Death Register. The components of the SNDS can be linked through a pseudonymized personal identification number (irreversible coding of beneficiaries’ identifiers, allowing reidentification by cross-referencing certain data). The pseudonymization aids in protecting individual privacy in the SNDS while maintaining the capability to link data across multiple databases. Access to the SNDS is tightly controlled and granted under strict regulations.

The DCIR database contains data on all outpatient services reimbursed by the French National Health Insurance, including drugs (coded according to the Anatomical Therapeutic Chemical Classification System [ATC]), outpatient visits, and laboratory tests, but does not provide information on medical indications, which can be deduced from other data. Patients with costly chronic diseases (LTD: long-term diseases), such as cancer, are 100% reimbursed for their health expenditure, and the diagnosis is recorded (coded according to the International Classification of Diseases, Tenth Revision [ICD-10]). The PMSI database also contains the procedures performed during hospital stays, as well as the principal (DP), related (DR), and associated (DA) diagnoses, all coded according to ICD-10. The DP corresponds to the diseases justifying admission to the hospital. To be recorded as a hospitalization diagnosis in the database (DP, DR, or DA), the diagnosis must have an impact on medical care. Procedures are coded using the French medical classification of clinical procedures (CCAM, Classification commune des actes médicaux). Drugs used during hospital stays are generally not available, except for certain high-cost drugs. Data available for hospital stays include admission date, discharge date, and procedure date.
