# Supplementary material for: First-trimester exposure to macrolides and risk of major congenital malformations compared with amoxicillin: A French nationwide cohort study
Source: PLoS Med. 2025 Apr 15;22(4):e1004576. doi: 10.1371/journal.pmed.1004576 (PMC12021278; doi:10.1371/journal.pmed.1004576)
Supplement: S1 Table — (DOCX) [file pmed.1004576.s002.docx]

**Table S1.** Algorithms to identify teratogenic infections or suspected teratogenic infections

| **Infection** |  | **Hospital discharge diagnoses codes (International Classification of Diseases, 10th revision)** | **Drugs (ATC class)** |
| --- | --- | --- | --- |
| Toxoplasmosis | Mother | Toxoplasmosis (B58) | Pyrimethamine (P01BD01) AND Sulfadiazine (J01EC02)  OR  Spiramycin (J01FA02) if at least three dispensing between date of conception and date of pregnancy outcome |
|  | Infant | Congenital toxoplasmosis (P371) | Pyrimethamine (P01BD01) AND Sulfadiazine (J01EC02) up to one year of life |
| Syphilis | Mother | Syphilis complicating pregnancy, childbirth, and the puerperium (O981)  Early syphilis (A51)  Late syphilis (A52)  Other and unspecified syphilis (A53)  Cardiovascular syphilis (I980)  Late syphilis of kidney (N290) | Benzylpenicillin (J01CE01) OR benzathine benzylpenicillin (J01CE08) |
|  | Infant | Congenital syphilis (A50) | Benzylpenicillin (J01CE01) OR benzathine benzylpenicillin (J01CE08) up to one year of life |
| Rubella | Mother | Rubella (B06)  Rubella arthritis (M014)  Maternal care for damage to fetus from maternal: cytomegalovirus/rubella (O353) |  |
|  | Infant | Congenital rubella syndrome (P350) |  |
| Cytomegalovirus | Mother | Cytomegaloviral disease (B25)  Cytomegaloviral mononucleosis (B271)  HIV disease resulting in Cytomegaloviral disease (B202)  Cytomegaloviral cholangitis (K8700)  Cytomegaloviral colitis (K93820)  Cytomegaloviral retinitis (H3200)  Maternal care for damage to fetus from maternal: cytomegalovirus/rubella (O353) |  |
|  | Infant | Congenital cytomegalovirus infection (P351) |  |
| Herpes | Mother | Herpes gestationis (O264), only PD/RD |  |
|  | Infant | Congenital herpes viral [herpes simplex] infection (P352), only PD/RD |  |
| Varicella | Mother | Varicella (B01) |  |
|  | Infant | Congenital varicella (P358) |  |
| Lymphocytic choriomeningitis virus | Mother | Lymphocytic choriomeningitis (A872) |  |
|  | Infant |  |  |
| Zika | Mother | Zika virus disease (A925) |  |
|  | Infant | Congenital Zika virus disease (P354) |  |
| - *For each diagnosis, one hospital discharge diagnosis code or one drug dispensing was sufficient to have a high sensitivity of diagnosis.* - *All codes were used to identify diseases including PD/RD/AD during all pregnancy: PD: Principal diagnoses. RD: Related diagnoses. AD: Associated diagnoses. For herpes, only PD/RD were used as intrauterine herpes is very rare compared to neonatal herpes and the risk of low specificity is greater.* - *Infant Hospital discharge diagnoses were retrieved up to two years of life.* | | | |
