# Supplementary material for: First-trimester exposure to macrolides and risk of major congenital malformations compared with amoxicillin: A French nationwide cohort study
Source: PLoS Med. 2025 Apr 15;22(4):e1004576. doi: 10.1371/journal.pmed.1004576 (PMC12021278; doi:10.1371/journal.pmed.1004576)
Supplement: S2 Table — (DOCX) [file pmed.1004576.s003.docx]

**Table S2.** List of known teratogenic drugs

| Misoprostol | Thalidomide | Vinorelbine |
| --- | --- | --- |
| Phenindione | Methotrexate | Etoposide |
| warfarin | valproic acid | Teniposide |
| acenocoumarol | Topiramate | Doxorubicin |
| fluindione | Venetoclax | Daunorubicin |
| Tazarotene | Cyclophosphamide | Epirubicin |
| Acitretin | Chlorambucil | Idarubicin |
| isotretinoin (oral) | Melphalan | Mitoxantrone |
| alitretinoin | Chlormethine | Bleomycin |
| Danazol | Busulfan | Mitomycin |
| Raloxifene | Lomustine | Procarbazine |
| Finasteride | Fotemustine | methyl amino levulinate |
| Dutasteride | Pipobroman | Bexarotene |
| Ribavirin | Dacarbazine | Ixazomib |
| Megestrol | Methotrexate | Vismodegib |
| Goserelin | Mercaptopurine | Sonidegib |
| tamoxifen | Pemetrexed | Olaparib |
| fulvestrant | Fludarabine | Niraparib |
| Bicalutamide | Cytarabine | Rucaparib |
| anastrozole | Fluorouracil | Asparaginase |
| exemestane | Capecitabine | Altretamine |
| Mycophenolic acid | Tegafur | Hydroxycarbamide |
| leflunomide | Trifluridine | Estramustine |
| teriflunomide | Vinblastine | Mitoguazone |
| cladribine | Vincristine | Anagrelide |
