# Supplementary material for: First-trimester exposure to macrolides and risk of major congenital malformations compared with amoxicillin: A French nationwide cohort study
Source: PLoS Med. 2025 Apr 15;22(4):e1004576. doi: 10.1371/journal.pmed.1004576 (PMC12021278; doi:10.1371/journal.pmed.1004576)
Supplement: S3 Table — (DOCX) [file pmed.1004576.s004.docx]

**Table S3.** Algorithms for identifying major congenital malformations in the SNDS

Major congenital malformations (MCMs) were defined according to the classification system of the European Surveillance of Congenital Anomalies (EUROCAT) Guideline. The presence of MCMs was identified using algorithms based on inpatient ICD-10 diagnostic codes, CCAM (“*Classification Commune des Actes Médicaux”)* codes for relevant surgery, or medical procedures in the records of live-born infants within one year after delivery, or up to two years for epispadias/exstrophy of urinary bladder, hypospadias, and severe microcephaly. The algorithms were constructed by the EPI-PHARE team with experts in malformation coding. We then compared the frequency of each subtype of MCMs observed among live births in the EPI-MERES register with data from the EUROCAT registry [1]. From 2010 to 2021, the prevalence of MCMs was 181 cases per 10,000 births in the EPI-MERES Register (compared to 204 in the EUROCAT database). The prevalence of individual MCM subtypes among live births was generally consistent with EUROCAT data (median relative difference: -3%).

| **Malformation Group or Subgroup** | **ICD-10 diagnosis codes,**  **Exclusion criteria and medical procedures when necessary** | **CCAM codes used for medical procedures, surgical repair, and imaging** |
| --- | --- | --- |
| **Nervous system anomalies** |  |  |
| Anencephaly | Q00 |  |
| Encephalocele and meningocele | Q01, do not included if associated with Anencephaly (Q00) |  |
| Spina Bifida | Q05, do not included if associated with Anencephaly (Q00) or Encephalocele (Q01) |  |
| Hydrocephaly | Q03, do not included if associated with Neural Tube defect group (Q00, Q01, Q05) |  |
| Severe microcephaly | Q02, do not included if associated with Neural Tube defect group (Q00, Q01, Q05) + at least 1 MRI within 2 years or death |  |
| Arhinencephaly/Holoprosencephaly | Q041, Q042, do not included if associated with Neural Tube defect group (Q00, Q01, Q05) |  |
| Agenesis of the corpus callosum | Q040, do not included if associated with Neural Tube defect group (Q00, Q01, Q05) |  |
| **Eye anomalies** |  |  |
| Cystic eyeball/Other anophthalmos/Microphthalmos | Q110, Q111, Q112 |  |
| Cystic eyeball/Other anophthalmos | Q110, Q111 |  |
| Congenital cataract | Q120 + specific medical procedures within 1 year or death | BFPA002, BGFA008, BFGA002, BGFA001, BFGA008 |
| Congenital glaucoma | Q150 + specific medical procedures within 1 year or death | BHQP002, BEFA008, BEPA003, BGFA014 |
| **Ear, face, and neck anomalies** |  |  |
| Anotia and atresia | Q160, Q161 |  |
| **Heart defects** |  |  |
| Common arterial truncus | Q200 |  |
| Double outlet right ventricle | Q201 |  |
| Double outlet left ventricle | Q202 |  |
| Complete transposition of great arteries (D-TGA) | Q203 |  |
| Corrected transposition of great arteries (L-TGA) | Q205 + surgical repair within 1 year OR death | DZMA010 |
| Single ventricle | Q204, do not included if associated with Hypoplastic left heart syndrome (Q234) or Hypoplastic right heart syndrome (Q226) |  |
| Ventricular septal defect | Q210 |  |
| Atrial septal defect | Q211 + at least one echography within 1 year OR death | DZQJ001, DZQJ006, DZQJ008, DZQJ009, DZQJ010, DZQJ011, DZQM00, DZQM006 |
| Atrioventricular septal defect | Q212 |  |
| Tetralogy of Fallot | Q213 |  |
| Tricuspid stenosis and atresia | Q224 |  |
| Ebstein’s anomaly | Q225 |  |
| Pulmonary valve stenosis | Q221 |  |
| Pulmonary valve atresia | Q220 |  |
| Aortic valve atresia/stenosis | Q230 |  |
| Mitral valve atresia/stenosis | Q232 + surgical repair within 1 year OR death |  |
| Hypoplastic left heart | Q234 |  |
| Hypoplastic right heart | Q226 |  |
| Coarctation of aorta | Q251 |  |
| Aortic atresia | Q252 |  |
| Anomalous pulmonary venous return | Q262 |  |
| PDA (Patent Ductus Arteriosus) as only CHD in term infants | Q250 + surgical closure within 1 year OR Q25O still present after 6 months OR death  AND not part of a ductus dependent congenital heart defects, namely: transposition of great arteries (Q203), hypoplastic left heart (Q234) and coarctation of aorta (Q251) | DASF001 |
| **Respiratory anomalies** |  |  |
| Choanal stenosis or atresia | Q300+ surgical repair within 1 year or death | GCME004, GCME003, GCME002, GCMA001, GCME001, [GCCD001](https://www.aideaucodage.fr/ccam-gccd001) |
| **Oro-facial clefts** |  |  |
| Cleft palate | Q35 exclude if associated with holoprosencephaly, anencephaly, or cleft lip subgroups |  |
| Cleft lip with or without cleft palate | Q36, Q37 |  |
| **Digestive system** |  |  |
| Esophageal atresia | Q390, Q391 |  |
| Duodenal atresia | Q410 |  |
| Atresia or stenosis of intestine | Q411-Q418 |  |
| Ano-rectal atresia or stenosis | Q420-Q423 + surgical repair within 1 year or death | HJAD001, HJEA001, HJEA002, HJEA003, HJEA004, HHCA002, HJMA001, HKEA001, HKMA006, HHCC007 |
| Hirschsprung’s disease | Q431 + surgical repair within 1 year or death | HJFD003, HJFC001, HJFA016, HHCA002, HHCC007 |
| Anomalies of intestinal fixation | Q433 |  |
| Atresia of bile ducts | Q442 + surgical repair within 1 year or death | HLCA001 |
| Annular pancreas | Q451 |  |
| Diaphragmatic hernia | Q790 |  |
| **Abdominal wall defects** | | |
| Gastroschisis | Q793+ surgical repair within 1 year or death | LMSA003, LMSA001, LMSA005 |
| Omphalocele | Q792 + surgical repair within 1 year or death | LMSA003, LMSA001, LMSA005 |
| **Anomalies of kidney and urinary tract** | | |
| Renal agenesis, unilateral | Q600 |  |
| Renal agenesis, bilateral/ Potter syndrome | Q601, Q606 |  |
| Multicystic enal dysplasia | Q614 |  |
| Hydronephrosis | Q620, Q621, Q623 exclude if associated with Q627 + at least 2 records for echography within 1 year or death | JAQM003, JAQM004, JAQJ001, JAQM001 |
| Lobulated, fused and horseshoe kidney | Q631, Q632 |  |
| Epispadias/ Exstrophy of urinary bladder | Q640, Q641 |  |
| Posterior urethral valves | Q642 + surgical repair within 1 year or death | JEFE005, JEPH001 |
| Prune belly syndrome | Q794 |  |
| **Genital anomalies** |  |  |
| Hypospadias | Q54 excluding Q544 + surgical repair within 1 or 2 years | JEMA006, JEMA014, JEMA019, JEMA020, JEMA021, JEMA011 |
| Indeterminate sex | Q56 |  |
| **Limb anomalies** |  |  |
| Limb reduction defects | Q71, Q72, Q73 |  |
| Club foot | Q660 + specific medical procedures within 1 year | NHRP003, NJAB001, PCPB002 |
| Hip dislocation | Q650, Q651, Q652 + surgical repair within 1 year OR at least 2 diagnostic tests within 1 year OR death | Surgery: NEEP003, NEEA004, ZEMP002, NEQP001, NEQH001, NEEP006, NEQP002, NZMP012, ZEMP010  diagnostic test: NEQM001, NEQH002, NAQK071, NEQC001 |
| Polydactyly | Q69 + surgical repair within 1 year OR death | MZFA008, MZFA012, MZFA015, MZFA014, NZFA011, NZFA012 |
| Syndactyly | Q70 + surgical repair within 1 year OR death | MJPA014, MZPA002, QDPA001 |
| **Other anomalies** |  |  |
| Craniosynostosis | Q750 + surgical repair within 1 year or death | LAFA900, LAMA006, LANC001, LAPA005, LAPA006, LAPA008, LAPA016, LAEA002, LAEA004, LAEA006, LAEA009, LARA001, LARA002, LARA003, LARA004 |
| Situs inversus | Q893 |  |
| Septo-optic dysplasia | Q044 |  |
| Vascular disruption anomalies | Q411, Q412, Q418, Q710, Q712, Q720, Q722, Q730, Q793 |  |
| Laterality anomalies | Q206, Q240, Q890, Q893 |  |
| Conjoined twins | Q894 |  |
| **Chromosomal anomaly/teratogenic infection (exclusion criteria)** |  |  |
| Skeletal dysplasia | Q77, Q780-Q788 |  |
| Down syndrome | Q90 |  |
| Trisomy 13/Patau syndrome | Q914-Q917 |  |
| Trisomy 18/Edwards syndrome | Q910-Q913 |  |
| Turner syndrome | Q96 |  |
| Triploidy and polyploidy | Q927 |  |
| Teratogenic syndromes resulting in major malformation | Q86, P350, P351, P354, P358, P371 (in teratogenic infection) |  |

**Reference**

1. Marty L, Miranda S, Weill A, Dray-Spira R. Prévalence des malformations congénitales majeures en France entre 2010 et 2021 : estimations parmi les neuf millions d’enfants et fœtus du registre EPI-MERES. Journal of Epidemiology and Population Health. 2024;72: 202373. doi:10.1016/j.jeph.2024.202373
