# Supplementary material for: First-trimester exposure to macrolides and risk of major congenital malformations compared with amoxicillin: A French nationwide cohort study
Source: PLoS Med. 2025 Apr 15;22(4):e1004576. doi: 10.1371/journal.pmed.1004576 (PMC12021278; doi:10.1371/journal.pmed.1004576)
Supplement: S4 Table — (DOCX) [file pmed.1004576.s005.docx]

**Table S4.** Definition of covariates included in the propensity score model and their assessment periods

| **Variable** | **Categories** | **Definition** |
| --- | --- | --- |
| Gestational age at the first exposure | Continuous | Gestational week when treatment of antibiotic of interest (macrolide or amoxicillin) was first started |
| **Socio-demographic** |  |  |
| Maternal age | Continuous | Maternal age at the pregnancy onset |
| Year of pregnancy | 2010/2011/2012/2012/2014/2015/2016/2017/2018/2019/2020 | Year of pregnancy end |
| Region of residence | Île-de-France (Paris), Centre-Val de Loire, Bourgogne-Franche-Comté, Normandie, Hauts-de-France, Grand Est, Pays de la Loire, Bretagne, Nouvelle-Aquitaine, Occitanie, Auvergne-Rhône-Alpes, Provence-Alpes-Côte d’Azur, Corse, French overseas territories |  |
| Complementary Healthcare Coverage | Yes/No |  |
| Deprivation index | Q1/Q2/Q3/Q4/Q5/Missing |  |
| **Pregnancy-related healthcare utilization** |  |  |
| Reimbursed folic acid supplementation | Yes/No | Having at least one fill for products containing folic acid from 3 months before pregnancy onset to the end of first trimester |
| Assisted reproduction | Yes/No | Having at least one reimbursement within 3 months before pregnancy onset for one of the following procedures: oocyte pick-up, embryo transfer, and artificial insemination. These procedures are fully covered by the French health insurance after medical validation and up to the maternal age of 43 years. |
| **Lifestyle factors** |  |  |
| Smoking-related conditions | Yes/No | Assessment period: spanning from five years prior to pregnancy until the end of pregnancy, including delivery hospitalizations.  Hospital discharge diagnoses (ICD-10 codes):  - Z716, F17, T652, Z720 (since 2006)  - I731, J41, J42, J43, J44, T652 (since 2014) (*)  Long-term disease status (ICD-10 codes):  - Z716, F17, T652, Z720 (since 2006)  Specific medications (ATC codes):  - N06AX12 or N07BA (since 2014)  - R03AC18, R03AC19, R03BB0,4 R03BB0,5 R03BB06, R03BB0,7 R03AL04, R03AL0,5 R03AK04, with (*)  Nicotine replacement therapies or a consultation on tobacco addictiveness |
| Alcohol-related conditions | Yes/No | Assessment period: spanning from five years prior to pregnancy until the end of pregnancy, including delivery hospitalizations.  Hospital discharge diagnoses (ICD-10 codes):  - F10, E244, G312, G621, G721, I426, K292, K70, K860, T51, Z502, Z714  Long-term disease status (ICD-10 codes):  - F10, E244, G312, G621, G721, I426, K292, K70, K860, T51, Z502, Z714  Specific medications (ATC codes):  - N07BB01, N07BB03, N07BB04, N07BB05 |
| Substance use disorders | Yes/No | Assessment period: spanning from five years prior to pregnancy until the end of pregnancy, including delivery hospitalizations.  Hospital discharge diagnoses (ICD-10 codes): F11  Long-term disease status (ICD-10 codes): F11 |
| **Proxies for pre-existing conditions** |  |  |
| Antihypertensive drug use | Yes/No | At least 3 fills for antihypertensive drugs in the previous year |
| Obesity-related hospital discharge/long-term disease diagnoses | Yes/No | Assessment period: spanning from five years prior to pregnancy until the end of pregnancy, including delivery hospitalizations.  Hospital discharge diagnoses (ICD-10 codes): E66  Long-term disease status (ICD-10 codes): E66 |
| Antidiabetic drug use or diabetes-related hospital discharge/long-term disease diagnoses | Yes/No | Hospital discharge diagnoses (ICD-10 codes):  - E10 to E14, G59.0*, G63.2*, G73.0*, G99.0*, H28.0*, H36.0*, I79.2*, L97*, M14.2*, M14.6*, N08.3*  Long-term disease status (ICD-10 codes): E10 to E14  Specific medications (ATC codes):  - Having at least 3 fills (on different dates) of oral or injectable antidiabetics (insulin or agonist GLP-1)  - Or having at least 2 fills when at least one 3-month supply is dispensed in the year n (the year prior index date) and/or in the year n-1]  - Medications in ATC2 A10 class (excluding benfluorex – code ATC A10BX06) |
| **Healthcare burden before pregnancy** |  |  |
| Prior hospitalization | Yes/No | Having at least one hospitalization not related to pregnancy and treatment cycles within 6 months before pregnancy onset |
| No. of consultations with general practitioners | Continuous | Number of consultation dates with general practitioners in outpatient settings within 6 months before pregnancy onset |
| No. of prescribed drugs not antibiotics | Continuous | Number of non-antibiotics prescribed for pregnant women within 6 months before pregnancy onset |
