# Supplementary material for: First-trimester exposure to macrolides and risk of major congenital malformations compared with amoxicillin: A French nationwide cohort study
Source: PLoS Med. 2025 Apr 15;22(4):e1004576. doi: 10.1371/journal.pmed.1004576 (PMC12021278; doi:10.1371/journal.pmed.1004576)
Supplement: S5 Table — (DOCX) [file pmed.1004576.s006.docx]

**S5 Table.** Description of antibiotic treatment during the first trimester and pregnancy outcome of the study cohort

|  | **Any macrolide** | **Azithromycin** | **Spiramycin** | **Clarithromycin** | **Josamycin** | **Roxithromycin** | **Erythromycin** | **Amoxicillin** |
| --- | --- | --- | --- | --- | --- | --- | --- | --- |
| **Variables** | **(n=140,708)** | **(n=42,585)** | **(n=35,359)** | **(n=21,527)** | **(n=19,100)** | **(n=18,257)** | **(n=7,462)** | **(n=592,652)** |
| **Antibiotic treatment** |  |  |  |  |  |  |  |  |
| **No. of exposed antibiotic agents** |  |  |  |  |  |  |  |  |
| 1 | 111,263 (79.1) | 33,157 (77.9) | 28,824 (81.7) | 16,411 (76.2) | 13,949 (73.0) | 13,838 (75.8) | 5,084 (68.1) | 496,752 (83.8) |
| 2 | 24,204 (17.2) | 7,640 (17.9) | 5,300 (15.0) | 4,144 (19.3) | 4,074 (21.3) | 3,560 (19.5) | 1,841 (24.7) | 82,224 (13.9) |
| >=3 | 5,241 (3.7) | 1,788 (4.2) | 1,135 (3.2) | 972 (4.5) | 1,077 (5.6) | 859 (4.7) | 537 (7.2) | 13,676 (2.3) |
| **No. of prescriptions filled for antibiotic of interest** |  |  |  |  |  |  |  |  |
| 1 | 126,210 (94.3) | 41,430 (97.3) | 32,928 (93.4) | 21,059 (97.8) | 18,353 (96.1) | 17,705 (97.0) | 6,944 (93.1) | 544,137 (91.8) |
| 2 | 7,185 (5.4) | 1,027 (2.4) | 2,324 (6.6) | 442 (2.1) | 709 (3.7) | 507 (2.8) | 456 (6.1) | 44,438 (7.5) |
| >=3 | 490 (0.4) | 128 (0.3) | 7 (0.0) | 26 (0.1) | 38 (0.2) | 45 (0.2) | 62 (0.8) | 4,077 (0.7) |
| **Maternal characteristics** |  |  |  |  |  |  |  |  |
| **Gestational age category** |  |  |  |  |  |  |  |  |
| Preterm |  |  |  |  |  |  |  |  |
| Extremely preterm | 387 (0.3) | 137 (0.3) | 73 (0.2) | 43 (0.2) | 62 (0.3) | 52 (0.3) | 27 (0.4) | 1,480 (0.2) |
| Very preterm | 777 (0.6) | 255 (0.6) | 179 (0.5) | 105 (0.5) | 90 (0.5) | 107 (0.6) | 62 (0.8) | 2,947 (0.5) |
| Moderate to late preterm | 7,443 (5.3) | 2,320 (5.4) | 1,740 (4.9) | 1,070 (5.0) | 1,049 (5.5) | 1,011 (5.5) | 438 (5.9) | 29,776 (5) |
| Full term | 130,848 (93.0) | 39,474 (92.7) | 32,975 (93.5) | 20,097 (93.4) | 17,755 (93.0) | 16,906 (92.6) | 6,882 (92.2) | 553,868 (93.5) |
| Late term | 1,253 (0.9) | 399 (0.9) | 292 (0.8) | 212 (1.0) | 144 (0.8) | 181 (1.0) | 53 (0.7) | 4,581 (0.8) |
| **Delivery method** |  |  |  |  |  |  |  |  |
| Vaginal birth | 109,757 (78.0) | 33,091 (77.7) | 27,766 (78.7) | 16,783 (78.0) | 14,961 (78.3) | 14,036 (76.9) | 5,779 (77.4) | 466,553 (78.7) |
| Programmed C-section | 10,618 (7.5) | 3,113 (7.3) | 2,582 (7.3) | 1,790 (8.3) | 1,447 (7.6) | 1,460 (8) | 525 (7) | 44,860 (7.6) |
| C-section, unspecified | 3 (0) | 2 (0) | 1 (0) | 0 (0) | 0 (0) | 0 (0) | 0 (0) | 28 (0) |
| C-section, urgent | 19,332 (13.7) | 6,041 (14.2) | 4,680 (13.3) | 2,803 (13) | 2,555 (13.4) | 2,641 (14.5) | 1,104 (14.8) | 76,617 (12.9) |
| Missing | 998 (0.7) | 338 (0.8) | 230 (0.7) | 151 (0.7) | 137 (0.7) | 120 (0.7) | 54 (0.7) | 4,594 (0.8) |
