# Supplementary material for: First-trimester exposure to macrolides and risk of major congenital malformations compared with amoxicillin: A French nationwide cohort study
Source: PLoS Med. 2025 Apr 15;22(4):e1004576. doi: 10.1371/journal.pmed.1004576 (PMC12021278; doi:10.1371/journal.pmed.1004576)
Supplement: S6 Table — (DOCX) [file pmed.1004576.s007.docx]

**S6 Table.** Standardized differences (%) of pregnancies exposed to macrolides overall/each of the six macrolides and amoxicillin during the first trimester (before and after propensity score adjustment)

|  | **Macrolides overall** | | **Azithromycin** | | **Spiramycin** | | **Clarithromycin** | | **Josamycin** | | **Roxithromycin** | | **Erythromycin** | |
| --- | --- | --- | --- | --- | --- | --- | --- | --- | --- | --- | --- | --- | --- | --- |
| **Characteristics** | **St. Diff. (%) before PS** | **St. Diff. (%) after PS** | **St. Diff. (%) before PS** | **St. Diff. (%) after PS** | **St. Diff. (%) before PS** | **St. Diff. (%) after PS** | **St. Diff. (%) before PS** | **St. Diff. (%) after PS** | **St. Diff. (%) before PS** | **St. Diff. (%) after PS** | **St. Diff. (%) before PS** | **St. Diff. (%) after PS** | **St. Diff. (%) before PS** | **St. Diff. (%) after PS** |
| GA at the first exposure | -34.7 | -0.8 | -73.7 | -0.8 | 34.7 | -0.5 | -89.5 | 0.9 | -2.3 | -0.1 | -65.1 | -1.5 | 12.6 | 0 |
| **Socio-demographic** |  |  |  |  |  |  |  |  |  |  |  |  |  |  |
| Maternal age | 2.8 | 0.3 | 4.2 | 0.6 | -1.8 | 0.0 | 2.9 | 0.1 | 3.1 | 0.2 | 8.2 | 0.1 | 1.4 | -0.3 |
| Complementary Health Coverage | -3.6 | -0.3 | 1.8 | -0.7 | -11 | 0.2 | -1.9 | -0.4 | -4.1 | 0.1 | -1.7 | -0.1 | -7.1 | 0.3 |
| Deprivation index, quintiles |  |  |  |  |  |  |  |  |  |  |  |  |  |  |
| Q1 (least deprived) | 2.8 | 0.3 | 8.2 | 0.7 | -3.3 | -0.3 | -2.5 | -0.2 | 4 | 0 | 2.6 | 0.9 | 11.4 | -0.8 |
| Q2 | -1.2 | 0.2 | 0.2 | 0.5 | -1.9 | 0 | -0.6 | 0.1 | -0.7 | -0.1 | -5.6 | -0.2 | 1.7 | -0.3 |
| Q3 | -1.3 | 0.3 | -0.7 | 0.4 | -1.3 | -0.1 | 1.1 | 0.1 | -2.6 | -0.1 | -3 | 0 | -5 | 0.1 |
| Q4 | -1.0 | 0.1 | -4 | 0.1 | 2.2 | 0.1 | 0.5 | 0 | -2.2 | -0.1 | 0.8 | 0.2 | -6.3 | 0.1 |
| Q5 (most deprived) | -2.4 | 0.0 | -8.9 | -0.2 | 4.2 | 0.3 | 0.5 | 0 | 0.1 | -0.1 | -2.9 | 0.1 | -10.5 | 0.2 |
| Missing | 6.1 | -1.5 | 9.8 | -2.3 | -1 | -0.2 | 1.5 | -0.1 | 2.7 | 0.6 | 13.8 | -1.5 | 14.4 | 1.3 |
| Year of delivery |  |  |  |  |  |  |  |  |  |  |  |  |  |  |
| 2010 | 5.7 | -0.4 | -13.3 | -0.5 | 14.9 | 0.7 | 18.7 | 0.8 | 4.4 | -0.2 | 8.5 | 0.2 | 10.5 | 0 |
| 2011 | 4.6 | -0.1 | -11.3 | -0.3 | 13.1 | 0.3 | 16.4 | 0.7 | 1.5 | -0.2 | 8.4 | 0.3 | 10 | 0 |
| 2012 | 4.2 | 0 | -6.8 | 0 | 9.1 | -0.2 | 11.2 | 0.2 | 7.3 | 0.1 | 6.3 | 0.3 | 6.3 | 0 |
| 2013 | 2.6 | 0.2 | -3.9 | 0.1 | 4.7 | -0.5 | 5 | -0.1 | 6.2 | 0 | 5.5 | 0.4 | 4.9 | 0 |
| 2014 | 0.7 | 0.2 | -0.8 | 0.2 | 0.1 | -0.6 | -2.1 | -0.2 | 5.2 | -0.1 | 3 | 0.2 | 4.2 | 0.1 |
| 2015 | -0.9 | 0.2 | 1.4 | 0.3 | -3.3 | -0.4 | -6 | -0.2 | 2 | -0.2 | 0.7 | 0.1 | -0.4 | 0.1 |
| 2016 | -2.4 | 0.2 | 2.4 | 0.3 | -4.9 | -0.4 | -9.1 | -0.3 | -0.5 | -0.1 | -4.5 | -0.2 | -3.1 | 0.1 |
| 2017 | -3.7 | 0 | 3.5 | 0.3 | -7.8 | -0.1 | -8.1 | -0.4 | -3.8 | 0.1 | -6.9 | -0.3 | -8.1 | 0 |
| 2018 | -4.1 | 0 | 6.3 | 0 | -10.7 | 0.3 | -13.2 | -0.4 | -6.2 | 0.3 | -6.7 | -0.3 | -5.4 | 0 |
| 2019 | -5.4 | -0.2 | 8.2 | -0.1 | -13.2 | 0.6 | -13.7 | -0.5 | -9.2 | 0.4 | -12 | -0.7 | -12.7 | -0.1 |
| 2020 | -4.2 | -0.1 | 13.0 | -0.4 | -14.1 | 0.7 | -15.0 | -0.5 | -13.2 | -0.2 | -9.6 | -0.5 | -16.3 | -0.1 |
| Region of residence |  |  |  |  |  |  |  |  |  |  |  |  |  |  |
| Ile-de-France (Paris) | -1.9 | 0.4 | 3.6 | 0.5 | -12.7 | -0.3 | -12.5 | -0.6 | 8.0 | 0.0 | -1.0 | 1.3 | 16.0 | -1.3 |
| Centre-Val-de-Loire | -2.6 | -0.1 | -6.5 | -0.3 | -0.1 | -0.2 | -2.8 | -0.2 | -0.4 | -0.1 | -0.8 | 0.1 | -4.3 | 0.2 |
| Bourgogne-Franche-Comté | 1.2 | -0.1 | -3.5 | 0.0 | 10.9 | 1.1 | 1.9 | 0.2 | 0.2 | -0.1 | -7.7 | -0.5 | -2.9 | 0.1 |
| Normandie | -0.2 | 0.1 | -3.0 | -0.1 | 0.1 | -0.3 | 4.0 | 0.1 | -2 | 0.0 | 3.7 | 0.3 | -1.3 | 0.1 |
| Hauts-de-France | 1.3 | 0.1 | -7.7 | -0.1 | 10.6 | 0.4 | 5.7 | 0.4 | 3.3 | -0.1 | -0.9 | 0.1 | -8.7 | 0.3 |
| Grand Est | 3.0 | -0.1 | 2.0 | 0.1 | 7.4 | 0.2 | 5.5 | 0.3 | 1.9 | -0.2 | -1.5 | 0.0 | -7.7 | 0.2 |
| Pays de la Loire | -1.8 | 0.0 | -2.2 | 0.1 | 2.2 | -0.1 | -2.4 | -0.1 | -3.3 | 0.0 | -5.2 | -0.3 | -7.2 | 0.1 |
| Bretagne | -2.2 | 0.4 | 2.1 | 1.3 | -2.0 | -0.2 | -2.5 | -0.1 | -7.3 | 0.2 | -7 | -0.4 | -4.8 | 0.1 |
| Nouvelle-Aquitaine | -1.0 | 0.2 | -3.8 | -0.1 | -0.4 | -0.3 | 1.0 | 0.1 | -6.3 | 0.1 | 8.9 | 0.4 | -5.9 | 0.2 |
| Occitanie | -1.1 | 0.1 | -1.1 | 0.0 | -2.5 | -0.2 | 5.4 | 0.1 | -3.7 | 0.0 | -3.6 | -0.1 | -1.8 | 0.1 |
| Auvergne-Rhone-Alpes | -2.1 | 0.0 | -2.4 | 0.3 | -4.1 | -0.3 | -3.2 | -0.2 | -1.8 | -0.1 | 0.8 | -0.3 | 3.0 | -0.3 |
| Provence-Alpes-Côte d’Azur | 1.3 | 0.2 | 8.4 | 0.3 | -4.9 | -0.2 | 3.2 | 0.0 | -0.1 | -0.1 | -4.7 | -0.2 | -3.2 | 0.1 |
| Corse | 2.0 | -0.3 | 2.3 | -0.1 | 4.0 | 0.6 | 0.4 | 0.0 | -1.2 | 0.0 | 3.2 | 0.0 | -0.4 | 0.0 |
| French overseas territories | 7.4 | -1.8 | 12.2 | -2.7 | -1.9 | -0.2 | 0.1 | -0.2 | 4.5 | 0.6 | 16.1 | -1.6 | 17.6 | 1.4 |
| **Pregnancy-related healthcare utilization** |  |  |  |  |  |  |  |  |  |  |  |  |  |  |
| Reimbursed folic acid supplementation | -1.1 | -0.1 | 7.1 | 0.3 | -3.7 | -0.1 | -13.6 | -0.4 | -0.9 | 0.1 | -4.1 | -1.2 | 8.9 | -0.2 |
| Assisted reproduction | 10.3 | -1.1 | 19.0 | -0.7 | 1.1 | 0.0 | -9.7 | -0.8 | 0.1 | 0.0 | 27.5 | -3.1 | 4.0 | 0.0 |
| **Lifestyle factors** |  |  |  |  |  |  |  |  |  |  |  |  |  |  |
| Smoking-related conditions | -0.1 | 0.0 | -1.1 | -0.1 | 1.4 | 0.1 | 2.6 | 0.2 | -0.4 | 0.0 | -0.2 | 0.0 | -6.8 | 0.1 |
| Alcohol-related conditions | 1.0 | -0.2 | 1.6 | -0.3 | -0.1 | 0.0 | 1.1 | 0.0 | -0.1 | 0.0 | 2 | 0.0 | 1.9 | 0.1 |
| Substance use disorders | -0.2 | -0.1 | 0.5 | -0.2 | -1.7 | 0.0 | 1.6 | 0.0 | -1.3 | 0.0 | 0.6 | 0.1 | -1.7 | 0.0 |
| **Proxies for pre-existing conditions** |  |  |  |  |  |  |  |  |  |  |  |  |  |  |
| Antihypertensive drug use | 0.7 | 0.0 | 0.5 | -0.1 | -0.2 | 0.0 | 1.4 | 0.0 | 0.8 | 0.1 | 2.7 | 0.1 | 0.8 | -0.1 |
| Obesity-related hospital discharge or long-term disease diagnoses | -1.8 | -0.1 | -1.8 | -0.3 | -3.4 | 0.0 | 0.5 | 0.0 | 0.0 | 0.1 | -0.5 | -0.3 | -7.5 | 0.0 |
| Antidiabetic drug use or diabetes-related hospital discharge/long-term disease diagnoses | -0.5 | -0.1 | 0.9 | 0.1 | -2.4 | 0.0 | -1.4 | -0.1 | 0.1 | 0.0 | -0.2 | -0.2 | 1.0 | 0.1 |
| **Healthcare burden before pregnancy** |  |  |  |  |  |  |  |  |  |  |  |  |  |  |
| Prior hospitalization | 1.2 | -0.1 | 1.5 | 0.1 | 0.2 | 0.0 | 1.1 | -0.1 | 0.9 | 0.1 | 2.8 | -0.2 | 2.5 | 0.1 |
| No. of consultations with general practitioners | 3.5 | 0.0 | 5.2 | -0.3 | -6.3 | -0.1 | 10.4 | 0.1 | 7.0 | 0.7 | 10.5 | -0.2 | 7.4 | 0.6 |
| No. of prescribed drugs not antibiotics | 5.0 | -0.2 | 7.5 | -0.6 | -5.6 | 0.0 | 12.6 | 0.3 | 7.4 | 0.7 | 14 | -0.7 | 4.2 | 0.1 |
