# Supplementary material for: First-trimester exposure to macrolides and risk of major congenital malformations compared with amoxicillin: A French nationwide cohort study
Source: PLoS Med. 2025 Apr 15;22(4):e1004576. doi: 10.1371/journal.pmed.1004576 (PMC12021278; doi:10.1371/journal.pmed.1004576)
Supplement: S7 Table — (DOCX) [file pmed.1004576.s008.docx]

**S7 Table**. Adjusted risk differences (per 10,000 live-born infants) of any MCM and 42 selected individual MCMs (sorted by the most common to the least common MCMs in the organ-specific groups) in pregnancies exposed to macrolides overall during the first trimester compared with amoxicillin

|  | **N exposed events** | |  |
| --- | --- | --- | --- |
| **Outcome** | **Macrolide exposure** | **Amoxicillin exposure** | **Adjusted RD** |
|  | **(N total=140,707)** | **(N total=592,643)** | **(95% CI)** |
| **Any MCM overall** | 2432 | 10176 | 1.10 (-6.64 to 8.84) |
| **Nervous system** |  |  |  |
| Severe microcephaly | 52 | 215 | 0.06 (-1.08 to 1.21) |
| Hydrocephaly | 43 | 131 | 0.87 (-0.13 to 1.86) |
| Spina Bifida | 36 | 86 | 1.15 (0.26 to 2.05) |
| Agenesis of the corpus callosum | 24 | 126 | -0.41 (-1.20 to 0.38) |
| **Eye anomalies** |  |  |  |
| Congenital cataract | 20 | 84 | 0.00 (-0.71 to 0.72) |
| **Heart defects** |  |  |  |
| Atrioventricular septal defect | 441 | 1715 | 2.22 (-1.07 to 5.52) |
| Atrial septal defect | 289 | 1305 | -1.79 (-4.51 to 0.93) |
| Congenital pulmonary valve | 58 | 196 | 0.61 (-0.58 to 1.81) |
| D-TGA | 51 | 179 | 0.69 (-0.41 to 1.79) |
| Coarctation of aorta | 48 | 227 | -0.56 (-1.69 to 0.57) |
| Tetralogy of Fallot | 42 | 181 | -0.12 (-1.16 to 0.91) |
| Ventricular septal defect | 25 | 97 | 0.05 (-0.74 to 0.85) |
| PDA as only CHD in term infants | 22 | 108 | -0.24 (-0.99 to 0.52) |
| Hypoplastic left heart | 22 | 79 | 0.28 (-0.44 to 1.01) |
| Aortic valve atresia/stenosis | 18 | 58 | 0.27 (-0.38 to 0.93) |
| Double outlet right ventricle | 16 | 54 | 0.30 (-0.31 to 0.91) |
| Pulmonary valve atresia | 14 | 71 | -0.32 (-0.95 to 0.31) |
| **Oro-facial clefts** |  |  |  |
| Cleft lip with and without cleft palate | 102 | 507 | -1.23 (-2.85 to 0.40) |
| Cleft palate | 59 | 314 | -1.08 (-2.32 to 0.17) |
| **Digestive system** |  |  |  |
| Ano-rectal atresia | 46 | 168 | 0.52 (-0.52 to 1.57) |
| Oesophageal atresia | 34 | 137 | 0.09 (-0.82 to 1.01) |
| Diaphragmatic hernia | 22 | 122 | -0.43 (-1.19 to 0.34) |
| Hirschrung's disease | 18 | 66 | 0.17 (-0.50 to 0.83) |
| Atresia or stenosis of intestine | 17 | 66 | 0.15 (-0.49 to 0.79) |
| Anomalies of intestinal fixation | 14 | 54 | 0.02 (-0.57 to 0.62) |
| **Abdominal wall defects** |  |  |  |
| Omphalocele | 21 | 92 | -0.05 (-0.76 to 0.67) |
| Gastroschisis | 17 | 64 | 0.31 (-0.31 to 0.93) |
| **Anomalies of kidney and urinary tract** |  |  |  |
| Hydronephrosis | 209 | 985 | -1.29 (-3.58 to 1.00) |
| Unilateral Renal Agenesis | 47 | 223 | -0.27 (-1.35 to 0.82) |
| Renal Dysplasia | 40 | 172 | -0.02 (-1.02 to 0.98) |
| Horseshoe kidney | 26 | 146 | -0.57 (-1.41 to 0.26) |
| Posterior urethral valve | 18 | 67 | 0.22 (-0.43 to 0.87) |
| **Genital anomalies** |  |  |  |
| Hypospadias | 346 | 1437 | 0.26 (-2.69 to 3.20) |
| **Limb anomalies** |  |  |  |
| Club foot | 131 | 559 | -0.18 (-2.00 to 1.64) |
| Polydactyly | 127 | 522 | 0.39 (-1.37 to 2.16) |
| Hip dislocation | 109 | 378 | 1.29 (-0.34 to 2.93) |
| Syndactyly | 31 | 67 | 0.87 (0.01 to 1.72) |
| Limb reduction defects | 27 | 148 | -0.65 (-1.52 to 0.21) |
| **Other anomalies** |  |  |  |
| Craniosynostoses | 55 | 254 | -0.55 (-1.75 to 0.65) |
| Vascular disruption anomalies | 43 | 183 | 0.10 (-0.93 to 1.13) |
| Laterality anomalies | 27 | 98 | 0.36 (-0.44 to 1.16) |
| Situs inversus | 18 | 57 | 0.40 (-0.24 to 1.04) |
