# Supplementary material for: First-trimester exposure to macrolides and risk of major congenital malformations compared with amoxicillin: A French nationwide cohort study
Source: PLoS Med. 2025 Apr 15;22(4):e1004576. doi: 10.1371/journal.pmed.1004576 (PMC12021278; doi:10.1371/journal.pmed.1004576)
Supplement: S8 Table — (DOCX) [file pmed.1004576.s009.docx]

**S8 Table.** Adjusted relative risks of any MCM and 42 selected individual MCMs (sorted by the most common to the least common MCMs in the organ-specific groups) in pregnancies exposed to macrolides overall compared with amoxicillin during a narrower exposure window

|  | **N exposed events** | |  |
| --- | --- | --- | --- |
| **Outcome** | **Macrolide exposure** | **Amoxicillin exposure** | **Adjusted RR** |
|  | **(N total=85,022)** | **(N total=463,018)** | **(95% CI)** |
| **Any MCM** | 1478 | 7931 | 1.02 (0.96-1.08) |
| **Nervous system** |  |  |  |
| Severe microcephaly | 27 | 169 | 0.89 (0.59-1.34) |
| Hydrocephaly | 24 | 98 | 1.30 (0.83-2.04) |
| Spina Bifida | 19 | 60 | 1.80 (1.07-3.01) |
| Agenesis of the corpus callosum | 14 | 94 | 0.83 (0.47-1.46) |
| **Eye anomalies** |  |  |  |
| Congenital cataract | 14 | 62 | 1.32 (0.74-2.36) |
| **Heart defects** |  |  |  |
| Atrioventricular septal defect | 254 | 1342 | 1.03 (0.90-1.18) |
| Atrial septal defect | 187 | 1006 | 1.02 (0.87-1.19) |
| Congenital pulmonary valve | 37 | 154 | 1.32 (0.92-1.90) |
| D-TGA | 34 | 144 | 1.28 (0.88-1.86) |
| Coarctation of aorta | 33 | 168 | 1.09 (0.75-1.58) |
| Tetralogy of Fallot | 26 | 144 | 1.00 (0.66-1.52) |
| Ventricular septal defect | 16 | 66 | 1.32 (0.76-2.28) |
| PDA as only CHD in term infants | 11 | 86 | 0.73 (0.39-1.37) |
| Hypoplastic left heart | 16 | 62 | 1.45 (0.83-2.52) |
| Aortic valve atresia/stenosis | 11 | 46 | 1.27 (0.66-2.46) |
| Double outlet right ventricle | 13 | 47 | 1.58 (0.85-2.92) |
| Pulmonary valve atresia | 9 | 56 | 0.85 (0.42-1.72) |
| **Oro-facial clefts** |  |  |  |
| Cleft lip with and without cleft palate | 59 | 414 | 0.78 (0.59-1.03) |
| Cleft palate | 37 | 246 | 1.00 (1.00-1.00) |
| **Digestive system** |  |  |  |
| Ano-rectal atresia | 27 | 140 | 1.07 (0.71-1.62) |
| Oesophageal atresia | 18 | 109 | 0.91 (0.55-1.50) |
| Diaphragmatic hernia | 12 | 96 | 0.69 (0.38-1.26) |
| Hirschrung's disease | 16 | 55 | 1.53 (0.88-2.68) |
| Atresia or stenosis of intestine | 11 | 55 | 1.07 (0.56-2.04) |
| Anomalies of intestinal fixation | 9 | 42 | 1.16 (0.56-2.39) |
| **Abdominal wall defects** |  |  |  |
| Omphalocele | 13 | 70 | 1.04 (0.58-1.89) |
| Gastroschisis | 7 | 60 | 0.63 (0.29-1.38) |
| **Anomalies of kidney and urinary tract** |  |  |  |
| Hydronephrosis | 132 | 757 | 0.96 (0.79-1.15) |
| Unilateral Renal Agenesis | 31 | 179 | 0.96 (0.65-1.41) |
| Renal Dysplasia | 30 | 136 | 1.21 (0.81-1.80) |
| Horseshoe kidney | 23 | 116 | 1.15 (0.73-1.80) |
| Posterior urethral valve | 11 | 53 | 1.18 (0.61-2.26) |
| **Genital anomalies** |  |  |  |
| Hypospadias | 201 | 1128 | 0.96 (0.83-1.12) |
| **Limb anomalies** |  |  |  |
| Club foot | 82 | 450 | 0.97 (0.77-1.23) |
| Polydactyly | 85 | 396 | 1.19 (0.94-1.51) |
| Hip dislocation | 63 | 297 | 1.16 (0.88-1.53) |
| Syndactyly | 21 | 46 | 2.38 (1.42-4.01) |
| Limb reduction defects | 16 | 114 | 0.77 (0.45-1.30) |
| **Other anomalies** |  |  |  |
| Craniosynostoses | 38 | 185 | 1.14 (0.80-1.61) |
| Vascular disruption anomalies | 23 | 158 | 0.78 (0.50-1.21) |
| Laterality anomalies | 17 | 86 | 1.08 (0.64-1.82) |
| Situs inversus | 12 | 51 | 1.26 (0.67-2.37) |
