# Supplementary material for: First-trimester exposure to macrolides and risk of major congenital malformations compared with amoxicillin: A French nationwide cohort study
Source: PLoS Med. 2025 Apr 15;22(4):e1004576. doi: 10.1371/journal.pmed.1004576 (PMC12021278; doi:10.1371/journal.pmed.1004576)
Supplement: S9 Table — (DOCX) [file pmed.1004576.s010.docx]

**S9 Table.** Adjusted relative risks of any MCM and 42 selected individual MCMs (sorted by the most common to the least common MCMs) in pregnancies exposed to each of the six macrolides compared with amoxicillin: results from the main analysis

|  | **Azithromycin** | **Spiramycin** | **Clarithromycin** | **Josamycin** | **Roxithromycin** | **Erythromycin** |
| --- | --- | --- | --- | --- | --- | --- |
| **Outcome** | **(n=42,583)** | **(n=35,259)** | **(n=21,525)** | **(n=19,099)** | **(n=18,257)** | **(n=7,460)** |
| ***Any MCM overall*** | *1.01 (0.94-1.09)* | *1.00 (0.92-1.09)* | *0.97 (0.87-1.08)* | *1.01 (0.90-1.12)* | *1.06 (0.95-1.18)* | *1.04 (0.88-1.24)* |
| Atrioventricular septal defect | 1.15 (0.96-1.37) | 1.01 (0.83-1.24) | 1.02 (0.79-1.32) | 1.13 (0.88-1.45) | 1.06 (0.81-1.39) | 1.33 (0.92-1.94) |
| Hypospadias | 1.08 (0.88-1.31) | 0.86 (0.68-1.09) | 1.18 (0.91-1.54) | 0.75 (0.53-1.04) | 1.21 (0.91-1.59) | 0.81 (0.49-1.35) |
| Atrial septal defect | 0.85 (0.68-1.06) | 0.91 (0.71-1.17) | 0.88 (0.65-1.20) | 1.02 (0.75-1.40) | 1.08 (0.80-1.47) | 0.99 (0.58-1.67) |
| Hydronephrosis | 0.85 (0.65-1.11) | 1.11 (0.86-1.43) | 0.82 (0.56-1.21) | 0.88 (0.61-1.28) | 0.81 (0.53-1.23) | 0.99 (0.56-1.74) |
| Club foot | 1.00 (0.72-1.40) | 0.80 (0.54-1.18) | 1.03 (0.66-1.61) | 1.07 (0.68-1.70) | 1.03 (0.64-1.67) | 0.90 (0.40-2.02) |
| Polydactyly | 1.14 (0.84-1.56) | 0.66 (0.41-1.06) | 0.98 (0.60-1.60) | **1.50 (1.01-2.23)** | 1.15 (0.72-1.85) | 0.85 (0.38-1.90) |
| Hip dislocation | 1.20 (0.82-1.76) | 1.26 (0.86-1.86) | 1.00 (0.58-1.73) | 1.26 (0.75-2.12) | 1.45 (0.88-2.38) | 0.86 (0.32-2.32) |
| Cleft lip with or without cleft palate | 0.81 (0.55-1.20) | 0.85 (0.57-1.25) | 1.01 (0.62-1.63) | 0.98 (0.60-1.62) | 0.57 (0.30-1.12) | 1.12 (0.53-2.36) |
| Cleft palate | 0.79 (0.49-1.29) | 0.81 (0.48-1.37) | 1.09 (0.60-1.96) | 0.69 (0.33-1.46) | 0.51 (0.21-1.25) | 0.51 (0.13-2.05) |
| Pulmonary valve stenosis | 0.94 (0.53-1.65) | **1.59 (0.98-2.59)** | 1.33 (0.71-2.49) | 0.79 (0.32-1.92) | 1.04 (0.50-2.18) | 1.25 (0.40-3.91) |
| Craniosynostose | 1.08 (0.70-1.68) | 0.83 (0.46-1.49) | 0.70 (0.33-1.51) | 1.00 (0.50-2.03) | 0.48 (0.18-1.30) | 1.36 (0.51-3.65) |
| Severe microcephaly | 1.17 (0.72-1.92) | 0.82 (0.43-1.56) | 1.05 (0.51-2.17) | 0.99 (0.47-2.10) | 0.83 (0.36-1.89) | 1.44 (0.54-3.89) |
| D-TGA | 1.07 (0.60-1.90) | 1.41 (0.83-2.40) | 0.82 (0.33-2.03) | 1.23 (0.58-2.63) | **2.04 (1.07-3.88)** | **1.89 (0.70-5.11)** |
| Coarctation of aorta | 0.82 (0.47-1.42) | 1.15 (0.68-1.94) | 0.95 (0.46-1.94) | 1.22 (0.63-2.38) | 0.39 (0.12-1.22) | **2.11 (0.94-4.76)** |
| Unilateral renal agenesis | 0.59 (0.30-1.17) | 1.30 (0.80-2.11) | 0.39 (0.12-1.24) | 0.56 (0.21-1.51) | **1.92 (1.06-3.47)** | 0.76 (0.19-3.07) |
| Ano-rectal atresia | 1.20 (0.67-2.14) | 0.97 (0.51-1.84) | **1.59 (0.79-3.18)** | 1.10 (0.49-2.49) | 1.28 (0.56-2.92) | 0.98 (0.24-3.95) |
| Hydrocephaly | 1.39 (0.77-2.49) | **1.94 (1.13-3.33)** | 1.22 (0.53-2.82) | 0.95 (0.35-2.56) | 1.02 (0.38-2.78) | **1.88 (0.60-5.93)** |
| Vascular disruption anomalies | 1.31 (0.76-2.27) | 0.77 (0.39-1.52) | 1.43 (0.72-2.86) | 0.83 (0.34-2.02) | 0.58 (0.18-1.83) | **1.78 (0.66-4.80)** |
| Tetralogy of Fallot | 0.65 (0.33-1.29) | 0.93 (0.49-1.77) | 0.87 (0.38-2.01) | 1.19 (0.56-2.54) | 1.09 (0.48-2.49) | **1.73 (0.64-4.67)** |
| Multicystic renal dysplasia | 0.93 (0.51-1.70) | 0.86 (0.44-1.69) | 0.49 (0.15-1.55) | 1.27 (0.60-2.70) | **1.56 (0.75-3.24)** | 0.95 (0.24-3.84) |
| Spina bifida | **1.58 (0.80-3.10)** | 1.20 (0.52-2.77) | **1.99 (0.84-4.70)** | **2.52 (1.16-5.45)** | **2.81 (1.28-6.16)** | 1.02 (0.14-7.34) |
| Esophageal atresia | 1.35 (0.76-2.38) | 1.15 (0.58-2.27) | 0.39 (0.09-1.58) | 0.90 (0.33-2.45) | 1.45 (0.64-3.33) | 0 |
| Syndactyly | **1.72 (0.83-3.54)** | **2.10 (1.04-4.25)** | **1.54 (0.60-3.99)** | **2.23 (0.90-5.54)** | **1.95 (0.77-4.97)** | 1.15 (0.16-8.35) |
| Limb reduction defects | 0.81 (0.40-1.62) | 0.90 (0.44-1.84) | 0.53 (0.17-1.68) | 1.07 (0.44-2.62) | 0.22 (0.03-1.59) | 0.59 (0.08-4.20) |
| Laterality anomalies | 0.83 (0.36-1.94) | 1.48 (0.72-3.08) | 0.75 (0.18-3.09) | 0.94 (0.30-2.98) | **1.91 (0.80-4.55)** | **1.61 (0.40-6.57)** |
| Lobulated, fused, and horseshoe kidney | 0.69 (0.33-1.43) | 1.36 (0.71-2.59) | 0.41 (0.10-1.67) | 1.09 (0.45-2.66) | 0.46 (0.11-1.88) | 1.11 (0.27-4.50) |
| Ventricular septal defect | **1.51 (0.80-2.84)** | 0.55 (0.17-1.74) | 0.79 (0.24-2.54) | 1.27 (0.47-3.45) | 1.46 (0.58-3.72) | **1.73 (0.64-4.67)** |
| Agenesis of the corpus callosum | 0.90 (0.45-1.79) | 1.20 (0.58-2.47) | 0.68 (0.21-2.18) | 0.24 (0.03-1.75) | 0.27 (0.04-1.95) | 1.32 (0.33-5.34) |
| PDA as only CHD in term infants | 0.70 (0.30-1.63) | 0.52 (0.17-1.65) | 0.56 (0.14-2.32) | 0.60 (0.15-2.41) | **1.95 (0.87-4.37)** | 0.86 (0.12-6.21) |
| Hypoplastic left heart | 1.21 (0.55-2.68) | **1.56 (0.72-3.42)** | 0.73 (0.18-3.06) | 1.17 (0.37-3.70) | 1.34 (0.42-4.32) | 1.10 (0.15-7.96) |
| Diaphragmatic hernia | 0.74 (0.33-1.63) | 0.71 (0.29-1.74) | 0.99 (0.36-2.72) | 0.80 (0.25-2.50) | 0.79 (0.25-2.54) | 0.70 (0.10-5.01) |
| Omphalocele | 0.70 (0.29-1.73) | 1.33 (0.61-2.89) | 0.60 (0.15-2.48) | 0.69 (0.17-2.81) | 1.46 (0.53-4.02) | 0.98 (0.14-7.04) |
| Congenital cataract | 0.48 (0.15-1.56) | **1.62 (0.78-3.36)** | 0.71 (0.17-2.97) | 1.09 (0.35-3.46) | 1.36 (0.47-3.96) | 0 |
| Aortic valve atresia/stenosis | **1.82 (0.81-4.08)** | **1.65 (0.71-3.87)** | 0.43 (0.06-3.16) | 1.04 (0.25-4.27) | 1.13 (0.27-4.68) | **1.52 (0.37-6.17)** |
| Posterior urethral valve | 1.14 (0.45-2.90) | 1.28 (0.51-3.19) | 0.90 (0.22-3.79) | 0.88 (0.22-3.59) | **2.26 (0.81-6.31)** | 0 |
| Hirschrung's disease | 1.42 (0.60-3.38) | 1.16 (0.47-2.91) | 0.84 (0.20-3.55) | 0.93 (0.23-3.79) | **1.93 (0.66-5.62)** | 0 |
| Situs inversus | 0.96 (0.34-2.71) | **1.85 (0.79-4.35)** | 0 | **1.62 (0.51-5.20)** | **2.73 (0.98-7.62)** | 1.43 (0.20-10.36) |
| Atresia or stenosis of intestine | **1.54 (0.67-3.53)** | 1.30 (0.52-3.25) | 0 | 1.40 (0.44-4.45) | 0.97 (0.23-4.16) | 1.18 (0.16-8.54) |
| Gastroschisis | **1.75 (0.69-4.42)** | 0.45 (0.11-1.85) | **3.81 (1.58-9.19)** | 1.02 (0.25-4.17) | 0.74 (0.10-5.39) | **2.56 (0.62-10.50)** |
| Double outlet right ventricle | 0.62 (0.15-2.57) | **1.57 (0.62-3.98)** | 0.70 (0.09-5.16) | **2.24 (0.81-6.20)** | 1.34 (0.32-5.63) | **3.01 (0.73-12.45)** |
| Pulmonary valve atresia | 0.98 (0.41-2.34) | 1.25 (0.50-3.11) | 1.07 (0.33-3.51) | 0 | 0 | 0 |
| Anomalies of intestinal fixation | 1.14 (0.45-2.91) | **1.71 (0.68-4.32)** | 0 | 0 | 1.14 (0.27-4.74) | **3.25 (0.79-13.39)** |
| Note: Cells containing zero are the malformations with zero exposed events. Cells containing point estimates (95% CI) in blue are those with aRR greater than 1.50. | | | | | | |
