# Supplementary material for: First-trimester exposure to macrolides and risk of major congenital malformations compared with amoxicillin: A French nationwide cohort study
Source: PLoS Med. 2025 Apr 15;22(4):e1004576. doi: 10.1371/journal.pmed.1004576 (PMC12021278; doi:10.1371/journal.pmed.1004576)
Supplement: S10 Table — (DOCX) [file pmed.1004576.s011.docx]

**S10 Table.** Relative risks of any MCM and 42 selected individual MCMs (*sorted by the most common to the least common MCMs in the organ-specific groups)* in pregnancies exposed to each of six individual macrolides during the first trimester compared with amoxicillin: results from main, sensitivity, and negative control analyses

*(Note: To simplify the result table, we did not present the number of exposed events for each macrolide exposure group)*

|  |  |  | **Relative risk (95% CI)** |  |  |
| --- | --- | --- | --- | --- | --- |
| **Outcome** | **Main analysis**  **(Unadjusted)** | **Main analysis**  **(Adjusted)** | **Monotherapy**  **(Adjusted)** | **Narrower exposure window (Adjusted)** | **Negative control analysis (Adjusted)** |
| **AZITHROMYCIN** |  |  |  |  |  |
| **Any MCM overall** | 1.03 (0.96-1.11) | 1.01 (0.94-1.09) | 0.97 (0.89-1.06) | 1.05 (0.94-1.18) | 0.92 (0.83-1.02) |
| **Nervous system** |  |  |  |  |  |
| Severe microcephaly | 1.23 (0.77-1.97) | 1.17 (0.72-1.92) | 1.07 (0.60-1.92) | 1.21 (0.59-2.48) | 0.59 (0.26-1.33) |
| Hydrocephaly | 1.38 (0.78-2.44) | 1.39 (0.77-2.49) | 1.02 (0.47-2.23) | 1.03 (0.37-2.84) | 1.48 (0.76-2.88) |
| Spina Bifida | 1.62 (0.84-3.11) | 1.58 (0.80-3.10) | 1.87 (0.91-3.84) | 1.45 (0.45-4.66) | 0.55 (0.13-2.24) |
| Agenesis of the corpus callosum | 0.99 (0.51-1.95) | 0.90 (0.45-1.79) | 0.99 (0.48-2.06) | 1.05 (0.38-2.86) | 0.90 (0.37-2.20) |
| **Eye anomalies** |  |  |  |  |  |
| Congenital cataract | 0.50 (0.16-1.57) | 0.48 (0.15-1.56) | 0.39 (0.09-1.68) | 1.30 (0.40-4.22) | 0.39 (0.05-2.80) |
| **Heart defects** |  |  |  |  |  |
| Atrioventricular septal defect | 1.15 (0.97-1.37) | 1.15 (0.96-1.37) | 1.10 (0.90-1.35) | 1.06 (0.81-1.39) | 0.75 (0.56-0.99) |
| Atrial septal defect | 0.91 (0.73-1.13) | 0.85 (0.68-1.06) | 0.86 (0.67-1.12) | 0.94 (0.68-1.29) | 0.90 (0.68-1.19) |
| Congenital pulmonary valve | 0.99 (0.58-1.71) | 0.94 (0.53-1.65) | 0.86 (0.45-1.65) | 0.92 (0.38-2.25) | 0.15 (0.02-1.04) |
| D-TGA | 1.01 (0.58-1.77) | 1.07 (0.60-1.90) | 1.03 (0.52-2.05) | 0.99 (0.43-2.26) | 1.27 (0.65-2.48) |
| Coarctation of aorta | 0.86 (0.50-1.47) | 0.82 (0.47-1.42) | 0.52 (0.24-1.13) | 0.97 (0.43-2.20) | 0.65 (0.27-1.57) |
| Tetralogy of Fallot | 0.69 (0.35-1.35) | 0.65 (0.33-1.29) | 0.55 (0.24-1.27) | 0.70 (0.26-1.91) | 0 |
| Ventricular septal defect | 1.72 (0.95-3.14) | 1.51 (0.80-2.84) | 0.97 (0.40-2.32) | 1.49 (0.54-4.13) | 0.61 (0.19-1.96) |
| PDA as only CHD in term infants | 0.77 (0.34-1.76) | 0.70 (0.30-1.63) | 0.54 (0.19-1.53) | 0.83 (0.26-2.65) | 1.61 (0.65-3.96) |
| Hypoplastic left heart | 1.23 (0.57-2.67) | 1.21 (0.55-2.68) | 0.70 (0.21-2.26) | 0.84 (0.20-3.47) | 1.54 (0.56-4.22) |
| Aortic valve atresia/stenosis | 1.68 (0.77-3.68) | 1.82 (0.81-4.08) | 2.04 (0.85-4.89) | 1.21 (0.29-5.05) | 1.22 (0.38-3.87) |
| Double outlet right ventricle | 0.52 (0.13-2.11) | 0.62 (0.15-2.57) | 0.79 (0.19-3.34) | 0.51 (0.07-3.71) | 1.21 (0.38-3.82) |
| Pulmonary valve atresia | 1.18 (0.51-2.71) | 0.98 (0.41-2.34) | 1.17 (0.46-3.00) | 1.26 (0.39-4.08) | 0.31 (0.04-2.27) |
| **Oro-facial clefts** |  |  |  |  |  |
| Cleft lip with and without cleft palate | 0.77 (0.53-1.12) | 0.81 (0.55-1.20) | 0.81 (0.52-1.28) | 0.53 (0.26-1.08) | 0.92 (0.58-1.45) |
| Cleft palate | 0.80 (0.50-1.28) | 0.79 (0.49-1.29) | 0.92 (0.55-1.54) | 0.97 (0.50-1.90) | 0.63 (0.28-1.42) |
| **Digestive system** |  |  |  |  |  |
| Ano-rectal atresia | 1.08 (0.61-1.89) | 1.20 (0.67-2.14) | 1.39 (0.75-2.55) | 0.79 (0.29-2.14) | 1.40 (0.69-2.85) |
| Oesophageal atresia | 1.42 (0.82-2.46) | 1.35 (0.76-2.38) | 1.60 (0.84-3.02) | 0.69 (0.22-2.19) | 0.33 (0.08-1.32) |
| Diaphragmatic hernia | 0.80 (0.37-1.71) | 0.74 (0.33-1.63) | 0.70 (0.28-1.76) | 1.44 (0.58-3.56) | 0.58 (0.18-1.83) |
| Hirschrung's disease | 1.27 (0.55-2.92) | 1.42 (0.60-3.38) | 0.96 (0.29-3.19) | 2.99 (1.27-7.04) | 0 |
| Atresia or stenosis of intestine | 1.48 (0.68-3.22) | 1.54 (0.67-3.53) | 1.73 (0.72-4.16) | 0.88 (0.21-3.62) | 0.81 (0.20-3.36) |
| Anomalies of intestinal fixation | 1.29 (0.52-3.22) | 1.14 (0.45-2.91) | 0.53 (0.13-2.23) | 1.03 (0.24-4.34) | 1.71 (0.53-5.48) |
| **Abdominal wall defects** |  |  |  |  |  |
| Omphalocele | 0.76 (0.31-1.84) | 0.70 (0.29-1.73) | 0.99 (0.39-2.53) | 0.74 (0.18-3.04) | 0.91 (0.29-2.88) |
| Gastroschisis | 1.09 (0.44-2.70) | 1.75 (0.69-4.42) | 1.82 (0.65-5.10) | 0.47 (0.06-3.40) | 1.95 (0.85-4.47) |
| **Anomalies of kidney and urinary tract** |  |  |  |  |  |
| Hydronephrosis | 0.83 (0.64-1.08) | 0.85 (0.65-1.11) | 0.71 (0.51-1.00) | 0.97 (0.67-1.42) | 1.21 (0.87-1.68) |
| Unilateral Renal Agenesis | 0.56 (0.29-1.09) | 0.59 (0.30-1.17) | 0.76 (0.38-1.53) | 0.81 (0.33-1.98) | 1.11 (0.57-2.15) |
| Renal Dysplasia | 0.97 (0.54-1.74) | 0.93 (0.51-1.70) | 1.08 (0.58-2.03) | 1.40 (0.65-3.00) | 0.62 (0.23-1.69) |
| Horseshoe kidney | 0.76 (0.37-1.55) | 0.69 (0.33-1.43) | 0.51 (0.20-1.28) | 1.20 (0.52-2.73) | 1.47 (0.69-3.16) |
| Posterior urethral valve | 1.04 (0.42-2.58) | 1.14 (0.45-2.90) | 0.85 (0.26-2.78) | 2.03 (0.73-5.66) | 1.40 (0.51-3.83) |
| **Genital anomalies** |  |  |  |  |  |
| Hypospadias | 1.08 (0.90-1.31) | 1.08 (0.88-1.31) | 0.98 (0.78-1.24) | 1.06 (0.79-1.43) | 0.77 (0.56-1.07) |
| **Limb anomalies** |  |  |  |  |  |
| Club foot | 1.05 (0.76-1.43) | 1.00 (0.72-1.40) | 1.09 (0.76-1.56) | 1.10 (0.70-1.73) | 0.72 (0.43-1.21) |
| Polydactyly | 1.23 (0.91-1.66) | 1.14 (0.84-1.56) | 1.04 (0.73-1.50) | 1.44 (0.95-2.18) | 1.20 (0.83-1.73) |
| Hip dislocation | 1.14 (0.79-1.65) | 1.20 (0.82-1.76) | 1.04 (0.67-1.61) | 0.96 (0.51-1.80) | 1.28 (0.79-2.08) |
| Syndactyly | 1.87 (0.93-3.75) | 1.72 (0.83-3.54) | 1.50 (0.63-3.60) | 2.71 (1.05-6.95) | 1.12 (0.35-3.58) |
| Limb reduction defects | 0.85 (0.43-1.66) | 0.81 (0.40-1.62) | 1.04 (0.51-2.13) | 0.69 (0.22-2.17) | 0.51 (0.16-1.61) |
| **Other anomalies** |  |  |  |  |  |
| Craniosynostoses | 1.26 (0.82-1.93) | 1.08 (0.70-1.68) | 0.90 (0.53-1.55) | 1.42 (0.75-2.70) | 1.45 (0.83-2.52) |
| Vascular disruption anomalies | 1.14 (0.67-1.93) | 1.31 (0.76-2.27) | 1.27 (0.68-2.39) | 0.50 (0.16-1.58) | 1.74 (0.99-3.05) |
| Laterality anomalies | 0.85 (0.37-1.94) | 0.83 (0.36-1.94) | 1.19 (0.51-2.79) | 1.09 (0.40-2.99) | 0.25 (0.03-1.78) |
| Situs inversus | 0.98 (0.35-2.69) | 0.96 (0.34-2.71) | 1.31 (0.46-3.76) | 1.29 (0.40-4.21) | 0 |
| **SPIRAMYCIN** |  |  |  |  |  |
| **Any MCM overall** | 0.99 (0.91-1.07) | 1.00 (0.92-1.09) | 1.00 (0.91-1.09) | 1.02 (0.93-1.11) | 0.98 (0.90-1.06) |
| **Nervous system** |  |  |  |  |  |
| Severe microcephaly | 0.78 (0.41-1.47) | 0.82 (0.43-1.56) | 0.75 (0.35-1.61) | 0.89 (0.47-1.70) | 0.89 (0.51-1.55) |
| Hydrocephaly | 1.92 (1.13-3.28) | 1.94 (1.13-3.33) | 2.17 (1.23-3.82) | 1.84 (1.03-3.30) | 1.18 (0.64-2.18) |
| Spina Bifida | 1.17 (0.51-2.68) | 1.20 (0.52-2.77) | 1.47 (0.63-3.42) | 1.17 (0.47-2.94) | 1.09 (0.51-2.36) |
| Agenesis of the corpus callosum | 1.07 (0.52-2.18) | 1.20 (0.58-2.47) | 1.07 (0.47-2.45) | 1.36 (0.66-2.81) | 0.52 (0.19-1.40) |
| **Eye anomalies** |  |  |  |  |  |
| Congenital cataract | 1.60 (0.77-3.31) | 1.62 (0.78-3.36) | 1.74 (0.79-3.82) | 1.68 (0.77-3.70) | 1.78 (0.86-3.70) |
| **Heart defects** |  |  |  |  |  |
| Atrioventricular septal defect | 1.01 (0.83-1.23) | 1.01 (0.83-1.24) | 1.04 (0.83-1.29) | 1.00 (0.81-1.23) | 0.86 (0.70-1.04) |
| Atrial septal defect | 0.86 (0.68-1.10) | 0.91 (0.71-1.17) | 0.85 (0.64-1.13) | 0.95 (0.73-1.22) | 0.94 (0.76-1.17) |
| Congenital pulmonary valve | 1.54 (0.95-2.50) | 1.59 (0.98-2.59) | 1.52 (0.88-2.64) | 1.63 (0.98-2.70) | 1.21 (0.72-2.05) |
| D-TGA | 1.41 (0.83-2.39) | 1.41 (0.83-2.40) | 0.91 (0.44-1.86) | 1.51 (0.89-2.59) | 1.18 (0.71-1.97) |
| Coarctation of aorta | 1.11 (0.66-1.87) | 1.15 (0.68-1.94) | 0.82 (0.42-1.60) | 1.12 (0.64-1.98) | 0.96 (0.57-1.62) |
| Tetralogy of Fallot | 0.93 (0.49-1.76) | 0.93 (0.49-1.77) | 0.68 (0.30-1.54) | 0.90 (0.46-1.78) | 1.29 (0.78-2.15) |
| Ventricular septal defect | 0.52 (0.16-1.64) | 0.55 (0.17-1.74) | 0.49 (0.12-2.00) | 0.43 (0.11-1.77) | 0.39 (0.12-1.22) |
| PDA as only CHD in term infants | 0.47 (0.15-1.47) | 0.52 (0.17-1.65) | 0.45 (0.11-1.82) | 0.56 (0.18-1.77) | 1.29 (0.63-2.66) |
| Hypoplastic left heart | 1.49 (0.69-3.23) | 1.56 (0.72-3.42) | 1.63 (0.70-3.81) | 1.93 (0.92-4.06) | 2.20 (1.13-4.27) |
| Aortic valve atresia/stenosis | 1.74 (0.75-4.03) | 1.65 (0.71-3.87) | 1.54 (0.61-3.90) | 1.77 (0.75-4.19) | 0.99 (0.40-2.46) |
| Double outlet right ventricle | 1.56 (0.62-3.89) | 1.57 (0.62-3.98) | 1.58 (0.56-4.46) | 1.69 (0.67-4.30) | 0.64 (0.20-2.03) |
| Pulmonary valve atresia | 1.18 (0.48-2.93) | 1.25 (0.50-3.11) | 1.20 (0.43-3.32) | 1.35 (0.54-3.38) | 0.81 (0.30-2.21) |
| **Oro-facial clefts** |  |  |  |  |  |
| Cleft lip with and without cleft palate | 0.90 (0.61-1.32) | 0.85 (0.57-1.25) | 0.87 (0.57-1.34) | 0.91 (0.62-1.35) | 0.94 (0.68-1.32) |
| Cleft palate | 0.80 (0.48-1.35) | 0.81 (0.48-1.37) | 0.87 (0.50-1.53) | 0.88 (0.52-1.49) | 0.75 (0.44-1.28) |
| **Digestive system** |  |  |  |  |  |
| Ano-rectal atresia | 1.00 (0.53-1.89) | 0.97 (0.51-1.84) | 1.19 (0.62-2.27) | 1.23 (0.68-2.22) | 1.02 (0.54-1.94) |
| Oesophageal atresia | 1.10 (0.56-2.17) | 1.15 (0.58-2.27) | 0.96 (0.42-2.20) | 1.19 (0.60-2.36) | 0.75 (0.37-1.52) |
| Diaphragmatic hernia | 0.69 (0.28-1.69) | 0.71 (0.29-1.74) | 0.93 (0.38-2.31) | 0.75 (0.30-1.84) | 0.57 (0.23-1.40) |
| Hirschrung's disease | 1.27 (0.51-3.16) | 1.16 (0.47-2.91) | 1.66 (0.66-4.21) | 1.22 (0.49-3.07) | 1.24 (0.54-2.87) |
| Atresia or stenosis of intestine | 1.27 (0.51-3.16) | 1.30 (0.52-3.25) | 0.63 (0.15-2.58) | 1.35 (0.54-3.41) | 1.67 (0.72-3.87) |
| Anomalies of intestinal fixation | 1.56 (0.62-3.89) | 1.71 (0.68-4.32) | 1.65 (0.59-4.65) | 1.76 (0.69-4.50) | 0.30 (0.04-2.17) |
| **Abdominal wall defects** |  |  |  |  |  |
| Omphalocele | 1.28 (0.59-2.76) | 1.33 (0.61-2.89) | 1.37 (0.59-3.17) | 1.24 (0.54-2.87) | 0.91 (0.37-2.23) |
| Gastroschisis | 0.53 (0.13-2.15) | 0.45 (0.11-1.85) | 0.26 (0.04-1.88) | 0.44 (0.11-1.80) | 0.88 (0.36-2.17) |
| **Anomalies of kidney and urinary tract** |  |  |  |  |  |
| Hydronephrosis | 1.11 (0.86-1.43) | 1.11 (0.86-1.43) | 1.16 (0.88-1.53) | 1.15 (0.88-1.49) | 1.05 (0.82-1.36) |
| Unilateral Renal Agenesis | 1.36 (0.84-2.19) | 1.30 (0.80-2.11) | 1.37 (0.80-2.33) | 1.31 (0.80-2.17) | 1.06 (0.63-1.78) |
| Renal Dysplasia | 0.88 (0.45-1.72) | 0.86 (0.44-1.69) | 0.66 (0.29-1.49) | 0.92 (0.47-1.81) | 0.72 (0.34-1.53) |
| Horseshoe kidney | 1.15 (0.61-2.18) | 1.36 (0.71-2.59) | 1.13 (0.52-2.43) | 1.61 (0.86-2.99) | 1.25 (0.66-2.38) |
| Posterior urethral valve | 1.25 (0.51-3.11) | 1.28 (0.51-3.19) | 1.03 (0.32-3.33) | 0.58 (0.14-2.39) | 0.18 (0.03-1.33) |
| **Genital anomalies** |  |  |  |  |  |
| Hypospadias | 0.88 (0.70-1.11) | 0.86 (0.68-1.09) | 0.82 (0.63-1.07) | 0.89 (0.70-1.14) | 0.91 (0.73-1.14) |
| **Limb anomalies** |  |  |  |  |  |
| Club foot | 0.81 (0.55-1.19) | 0.80 (0.54-1.18) | 0.90 (0.59-1.35) | 0.78 (0.52-1.17) | 0.88 (0.61-1.25) |
| Polydactyly | 0.58 (0.36-0.93) | 0.66 (0.41-1.06) | 0.64 (0.38-1.08) | 0.65 (0.39-1.07) | 1.22 (0.90-1.68) |
| Hip dislocation | 1.33 (0.91-1.96) | 1.26 (0.86-1.86) | 1.29 (0.86-1.93) | 1.28 (0.86-1.92) | 0.85 (0.56-1.29) |
| Syndactyly | 2.26 (1.13-4.53) | 2.10 (1.04-4.25) | 2.15 (0.97-4.78) | 2.64 (1.28-5.43) | 0.93 (0.38-2.30) |
| Limb reduction defects | 0.91 (0.45-1.85) | 0.90 (0.44-1.84) | 0.94 (0.44-2.02) | 0.87 (0.40-1.87) | 0.81 (0.40-1.66) |
| **Other anomalies** |  |  |  |  |  |
| Craniosynostoses | 0.79 (0.45-1.42) | 0.83 (0.46-1.49) | 0.75 (0.38-1.46) | 0.94 (0.52-1.69) | 0.90 (0.54-1.50) |
| Vascular disruption anomalies | 0.83 (0.42-1.61) | 0.77 (0.39-1.52) | 0.50 (0.21-1.23) | 0.79 (0.40-1.54) | 1.08 (0.64-1.83) |
| Laterality anomalies | 1.37 (0.67-2.82) | 1.48 (0.72-3.08) | 1.67 (0.76-3.65) | 1.45 (0.70-3.02) | 1.20 (0.56-2.60) |
| Situs inversus | 1.77 (0.76-4.10) | 1.85 (0.79-4.35) | 1.93 (0.76-4.92) | 1.79 (0.76-4.21) | 1.47 (0.58-3.68) |
| **CLARITHROMYCIN** |  |  |  |  |  |
| **Any MCM overall** | 0.96 (0.87-1.07) | 0.97 (0.87-1.08) | 0.97 (0.86-1.09) | 1.08 (0.92-1.28) | 0.75 (0.61-0.94) |
| **Nervous system** |  |  |  |  |  |
| Severe microcephaly | 1.02 (0.51-2.07) | 1.05 (0.51-2.17) | 1.36 (0.66-2.84) | 0 | 1.72 (0.64-4.62) |
| Hydrocephaly | 1.26 (0.56-2.86) | 1.22 (0.53-2.82) | 1.70 (0.73-3.98) | 0.58 (0.08-4.21) | 0.68 (0.10-4.88) |
| Spina Bifida | 1.92 (0.84-4.39) | 1.99 (0.84-4.70) | 2.18 (0.85-5.57) | 4.55 (1.64-12.68) | 0 |
| Agenesis of the corpus callosum | 0.66 (0.21-2.06) | 0.68 (0.21-2.18) | 0.83 (0.26-2.65) | 0 | 0.69 (0.10-4.91) |
| **Eye anomalies** |  |  |  |  |  |
| Congenital cataract | 0.66 (0.16-2.67) | 0.71 (0.17-2.97) | 0.49 (0.07-3.62) | 0 | 0 |
| **Heart defects** |  |  |  |  |  |
| Atrioventricular septal defect | 1.01 (0.79-1.30) | 1.02 (0.79-1.32) | 1.15 (0.88-1.52) | 0.93 (0.61-1.42) | 0.78 (0.47-1.29) |
| Atrial septal defect | 0.95 (0.71-1.28) | 0.88 (0.65-1.20) | 0.95 (0.68-1.34) | 1.32 (0.88-1.99) | 0.66 (0.36-1.23) |
| Congenital pulmonary valve | 1.55 (0.84-2.84) | 1.33 (0.71-2.49) | 1.04 (0.48-2.25) | 2.17 (0.95-4.94) | 1.07 (0.26-4.29) |
| D-TGA | 0.77 (0.32-1.87) | 0.82 (0.33-2.03) | 0.93 (0.34-2.55) | 0.40 (0.06-2.88) | 1.43 (0.46-4.48) |
| Coarctation of aorta | 0.97 (0.48-1.96) | 0.95 (0.46-1.94) | 0.77 (0.31-1.90) | 0.71 (0.17-2.87) | 1.33 (0.43-4.15) |
| Tetralogy of Fallot | 0.91 (0.40-2.06) | 0.87 (0.38-2.01) | 0.74 (0.27-2.03) | 1.30 (0.41-4.11) | 0.50 (0.07-3.57) |
| Ventricular septal defect | 0.85 (0.27-2.69) | 0.79 (0.24-2.54) | 1.12 (0.34-3.64) | 1.96 (0.48-8.07) | 1.73 (0.43-7.01) |
| PDA as only CHD in term infants | 0.51 (0.13-2.06) | 0.56 (0.14-2.32) | 0.35 (0.05-2.55) | 0 | 0 |
| Hypoplastic left heart | 0.70 (0.17-2.84) | 0.73 (0.18-3.06) | 1.07 (0.25-4.48) | 0.97 (0.13-7.05) | 1.40 (0.20-10.10) |
| Aortic valve atresia/stenosis | 0.47 (0.07-3.43) | 0.43 (0.06-3.16) | 0.53 (0.07-3.92) | 1.13 (0.15-8.31) | 0 |
| Double outlet right ventricle | 0.51 (0.07-3.69) | 0.70 (0.09-5.16) | 0.96 (0.13-7.23) | 1.28 (0.17-9.35) | 2.63 (0.65-10.71) |
| Pulmonary valve atresia | 1.16 (0.37-3.69) | 1.07 (0.33-3.51) | 0.95 (0.22-3.98) | 0.97 (0.13-7.10) | 0 |
| **Oro-facial clefts** |  |  |  |  |  |
| Cleft lip with and without cleft palate | 0.98 (0.61-1.56) | 1.01 (0.62-1.63) | 0.96 (0.55-1.69) | 0.86 (0.38-1.93) | 0.84 (0.35-2.03) |
| Cleft palate | 1.05 (0.59-1.87) | 1.09 (0.60-1.96) | 1.23 (0.65-2.35) | 1.41 (0.62-3.19) | 0 |
| **Digestive system** |  |  |  |  |  |
| Ano-rectal atresia | 1.47 (0.75-2.88) | 1.59 (0.79-3.18) | 1.62 (0.74-3.55) | 1.23 (0.39-3.87) | 1.89 (0.60-5.92) |
| Oesophageal atresia | 0.40 (0.10-1.62) | 0.39 (0.09-1.58) | 0.54 (0.13-2.20) | 0.54 (0.08-3.89) | 0.57 (0.08-4.10) |
| Diaphragmatic hernia | 0.90 (0.33-2.44) | 0.99 (0.36-2.72) | 1.31 (0.47-3.64) | 0 | 0 |
| Hirschrung's disease | 0.83 (0.20-3.41) | 0.84 (0.20-3.55) | 0.58 (0.08-4.29) | 1.95 (0.47-8.18) | 0 |
| Atresia or stenosis of intestine | 0 | 0 | 0 | 0 | 0 |
| Anomalies of intestinal fixation | 0 | 0 | 0 | 0 | 0 |
| **Abdominal wall defects** |  |  |  |  |  |
| Omphalocele | 0.60 (0.15-2.43) | 0.60 (0.15-2.48) | 0.41 (0.06-2.99) | 0 | 0 |
| Gastroschisis | 2.58 (1.12-5.96) | 3.81 (1.58-9.19) | 3.18 (1.10-9.16) | 2.26 (0.55-9.33) | 0 |
| **Anomalies of kidney and urinary tract** |  |  |  |  |  |
| Hydronephrosis | 0.78 (0.54-1.14) | 0.82 (0.56-1.21) | 0.78 (0.50-1.23) | 0.88 (0.48-1.60) | 0.64 (0.29-1.43) |
| Unilateral Renal Agenesis | 0.37 (0.12-1.16) | 0.39 (0.12-1.24) | 0.55 (0.17-1.73) | 0 | 0 |
| Renal Dysplasia | 0.48 (0.15-1.50) | 0.49 (0.15-1.55) | 0.65 (0.20-2.06) | 1.00 (0.25-4.03) | 2.60 (0.96-7.00) |
| Horseshoe kidney | 0.38 (0.09-1.52) | 0.41 (0.10-1.67) | 0 | 1.59 (0.50-5.06) | 1.49 (0.37-6.01) |
| Posterior urethral valve | 0.82 (0.20-3.35) | 0.90 (0.22-3.79) | 1.13 (0.27-4.77) | 1.26 (0.17-9.17) | 0 |
| **Genital anomalies** |  |  |  |  |  |
| Hypospadias | 1.17 (0.90-1.51) | 1.18 (0.91-1.54) | 1.17 (0.86-1.58) | 1.43 (0.97-2.09) | 0.65 (0.34-1.25) |
| **Limb anomalies** |  |  |  |  |  |
| Club foot | 1.03 (0.67-1.60) | 1.03 (0.66-1.61) | 0.71 (0.39-1.31) | 1.26 (0.67-2.37) | 0.71 (0.27-1.90) |
| Polydactyly | 0.90 (0.55-1.45) | 0.98 (0.60-1.60) | 0.95 (0.54-1.66) | 1.37 (0.70-2.66) | 0.34 (0.09-1.37) |
| Hip dislocation | 1.02 (0.60-1.74) | 1.00 (0.58-1.73) | 0.77 (0.39-1.50) | 0.62 (0.20-1.92) | 1.89 (0.94-3.80) |
| Syndactyly | 2.05 (0.83-5.10) | 1.54 (0.60-3.99) | 2.18 (0.83-5.75) | 2.57 (0.62-10.75) | 2.77 (0.68-11.25) |
| Limb reduction defects | 0.56 (0.18-1.75) | 0.53 (0.17-1.68) | 0.48 (0.12-1.96) | 1.09 (0.27-4.44) | 0.66 (0.09-4.70) |
| **Other anomalies** |  |  |  |  |  |
| Craniosynostoses | 0.76 (0.36-1.61) | 0.70 (0.33-1.51) | 0.83 (0.36-1.89) | 1.28 (0.47-3.46) | 0.68 (0.17-2.74) |
| Vascular disruption anomalies | 1.35 (0.69-2.64) | 1.43 (0.72-2.86) | 1.07 (0.43-2.67) | 1.56 (0.58-4.24) | 0 |
| Laterality anomalies | 0.56 (0.14-2.28) | 0.75 (0.18-3.09) | 1.07 (0.26-4.42) | 0.69 (0.09-4.94) | 4.23 (1.56-11.49) |
| Situs inversus | 0 | 0 | 0 | 0 | 3.78 (0.92-15.47) |
| **ROXITHROMYCIN** |  |  |  |  |  |
| **Any MCM overall** | 1.06 (0.95-1.18) | 1.06 (0.95-1.18) | 1.05 (0.92-1.19) | 1.00 (0.85-1.18) | 1.10 (0.95-1.28) |
| **Nervous system** |  |  |  |  |  |
| Severe microcephaly | 0.91 (0.40-2.04) | 0.83 (0.36-1.89) | 0.71 (0.25-1.95) | 0.67 (0.17-2.73) | 1.14 (0.42-3.06) |
| Hydrocephaly | 0.99 (0.37-2.68) | 1.02 (0.38-2.78) | 0.71 (0.17-2.88) | 0.53 (0.07-3.83) | 0.88 (0.22-3.56) |
| Spina Bifida | 2.64 (1.22-5.71) | 2.81 (1.28-6.16) | 3.27 (1.40-7.66) | 1.91 (0.46-7.88) | 0 |
| Agenesis of the corpus callosum | 0.26 (0.04-1.84) | 0.27 (0.04-1.95) | 0.34 (0.05-2.47) | 0 | 1.10 (0.27-4.44) |
| **Eye anomalies** |  |  |  |  |  |
| Congenital cataract | 1.55 (0.57-4.22) | 1.36 (0.47-3.96) | 1.17 (0.33-4.15) | 2.08 (0.50-8.57) | 0 |
| **Heart defects** |  |  |  |  |  |
| Atrioventricular septal defect | 1.08 (0.83-1.40) | 1.06 (0.81-1.39) | 1.01 (0.74-1.39) | 1.10 (0.75-1.61) | 1.09 (0.76-1.56) |
| Atrial septal defect | 1.09 (0.81-1.48) | 1.08 (0.80-1.47) | 0.97 (0.67-1.41) | 1.15 (0.75-1.76) | 0.99 (0.64-1.52) |
| Congenital pulmonary valve | 1.32 (0.65-2.69) | 1.04 (0.50-2.18) | 0.95 (0.40-2.23) | 0.72 (0.18-2.92) | 1.41 (0.52-3.79) |
| D-TGA | 1.81 (0.96-3.43) | 2.04 (1.07-3.88) | 2.61 (1.32-5.16) | 1.49 (0.55-4.05) | 1.03 (0.33-3.23) |
| Coarctation of aorta | 0.43 (0.14-1.34) | 0.39 (0.12-1.22) | 0.17 (0.02-1.22) | 0.69 (0.17-2.77) | 1.47 (0.61-3.57) |
| Tetralogy of Fallot | 0.53 (0.17-1.67) | 0.58 (0.18-1.83) | 0.25 (0.03-1.81) | 0.36 (0.05-2.56) | 0.99 (0.32-3.09) |
| Ventricular septal defect | 1.08 (0.48-2.43) | 1.09 (0.48-2.49) | 0.73 (0.23-2.30) | 0.78 (0.19-3.14) | 1.71 (0.70-4.16) |
| PDA as only CHD in term infants | 1.67 (0.68-4.11) | 1.46 (0.58-3.72) | 1.85 (0.68-5.00) | 3.42 (1.24-9.46) | 2.36 (0.87-6.39) |
| Hypoplastic left heart | 1.23 (0.39-3.90) | 1.34 (0.42-4.32) | 0.59 (0.08-4.36) | 1.94 (0.47-8.00) | 1.00 (0.14-7.18) |
| Aortic valve atresia/stenosis | 2.10 (0.98-4.52) | 1.95 (0.87-4.37) | 1.75 (0.68-4.51) | 1.33 (0.33-5.43) | 2.10 (0.67-6.64) |
| Double outlet right ventricle | 1.23 (0.39-3.90) | 1.34 (0.42-4.32) | 0.59 (0.08-4.36) | 1.94 (0.47-8.00) | 1.00 (0.14-7.18) |
| Pulmonary valve atresia | 1.12 (0.27-4.58) | 1.13 (0.27-4.68) | 0.74 (0.10-5.42) | 1.11 (0.15-8.07) | 0 |
| **Oro-facial clefts** |  |  |  |  |  |
| Cleft lip with and without cleft palate | 0.58 (0.30-1.11) | 0.57 (0.30-1.12) | 0.52 (0.23-1.18) | 0 | 1.28 (0.70-2.32) |
| Cleft palate | 0.52 (0.21-1.25) | 0.51 (0.21-1.25) | 0.54 (0.20-1.47) | 0.21 (0.03-1.53) | 0.96 (0.36-2.59) |
| **Digestive system** |  |  |  |  |  |
| Ano-rectal atresia | 1.16 (0.51-2.62) | 1.28 (0.56-2.92) | 1.66 (0.73-3.80) | 0.83 (0.21-3.37) | 0 |
| Oesophageal atresia | 1.42 (0.63-3.22) | 1.45 (0.64-3.33) | 0.70 (0.17-2.85) | 1.10 (0.27-4.47) | 0.82 (0.20-3.33) |
| Diaphragmatic hernia | 0.80 (0.25-2.51) | 0.79 (0.25-2.54) | 1.05 (0.32-3.43) | 0 | 0 |
| Hirschrung's disease | 1.97 (0.72-5.40) | 1.93 (0.66-5.62) | 1.77 (0.48-6.51) | 0.98 (0.14-7.12) | 1.88 (0.46-7.66) |
| Atresia or stenosis of intestine | 0.98 (0.24-4.02) | 0.97 (0.23-4.16) | 0.59 (0.08-4.53) | 0.99 (0.14-7.17) | 1.26 (0.17-9.10) |
| Anomalies of intestinal fixation | 1.20 (0.29-4.93) | 1.14 (0.27-4.74) | 0.68 (0.09-4.99) | 1.33 (0.18-9.79) | 2.54 (0.62-10.47) |
| **Abdominal wall defects** |  |  |  |  |  |
| Omphalocele | 1.41 (0.52-3.84) | 1.46 (0.53-4.02) | 2.00 (0.72-5.56) | 1.53 (0.37-6.26) | 1.60 (0.39-6.48) |
| Gastroschisis | 0.51 (0.07-3.66) | 0.74 (0.10-5.39) | 0 | 0 | 0.79 (0.11-5.71) |
| **Anomalies of kidney and urinary tract** |  |  |  |  |  |
| Hydronephrosis | 0.76 (0.50-1.15) | 0.81 (0.53-1.23) | 0.90 (0.57-1.43) | 0.71 (0.37-1.37) | 1.09 (0.64-1.84) |
| Unilateral Renal Agenesis | 1.75 (0.98-3.12) | 1.92 (1.06-3.47) | 2.22 (1.16-4.27) | 1.30 (0.48-3.52) | 1.21 (0.45-3.25) |
| Renal Dysplasia | 1.51 (0.74-3.07) | 1.56 (0.75-3.24) | 1.99 (0.94-4.18) | 2.54 (1.12-5.77) | 1.79 (0.66-4.84) |
| Horseshoe kidney | 0.44 (0.11-1.79) | 0.46 (0.11-1.88) | 0.56 (0.14-2.33) | 1.05 (0.26-4.28) | 1.05 (0.26-4.24) |
| Posterior urethral valve | 1.94 (0.71-5.31) | 2.26 (0.81-6.31) | 1.45 (0.35-6.05) | 3.88 (1.21-12.51) | 0 |
| **Genital anomalies** |  |  |  |  |  |
| Hypospadias | 1.22 (0.93-1.60) | 1.21 (0.91-1.59) | 1.22 (0.89-1.67) | 1.00 (0.64-1.55) | 1.18 (0.78-1.78) |
| **Limb anomalies** |  |  |  |  |  |
| Club foot | 1.05 (0.65-1.67) | 1.03 (0.64-1.67) | 1.06 (0.61-1.83) | 1.06 (0.55-2.05) | 0.61 (0.25-1.48) |
| Polydactyly | 1.12 (0.70-1.79) | 1.15 (0.72-1.85) | 1.07 (0.61-1.86) | 1.86 (1.07-3.25) | 1.04 (0.54-2.01) |
| Hip dislocation | 1.46 (0.90-2.37) | 1.45 (0.88-2.38) | 1.21 (0.67-2.20) | 1.54 (0.76-3.11) | 1.50 (0.78-2.91) |
| Syndactyly | 2.42 (0.98-6.01) | 1.95 (0.77-4.97) | 1.15 (0.28-4.79) | 2.24 (0.54-9.30) | 1.68 (0.41-6.83) |
| Limb reduction defects | 0.22 (0.03-1.57) | 0.22 (0.03-1.59) | 0.28 (0.04-2.04) | 0 | 1.35 (0.43-4.25) |
| **Other anomalies** |  |  |  |  |  |
| Craniosynostoses | 0.51 (0.19-1.37) | 0.48 (0.18-1.30) | 0.32 (0.08-1.33) | 0.64 (0.16-2.57) | 1.54 (0.69-3.46) |
| Vascular disruption anomalies | 0.53 (0.17-1.67) | 0.58 (0.18-1.83) | 0.25 (0.03-1.81) | 0.36 (0.05-2.56) | 0.99 (0.32-3.09) |
| Laterality anomalies | 1.99 (0.87-4.53) | 1.91 (0.80-4.55) | 2.05 (0.73-5.80) | 1.24 (0.30-5.06) | 1.48 (0.37-6.02) |
| Situs inversus | 2.28 (0.83-6.28) | 2.73 (0.98-7.62) | 1.84 (0.44-7.63) | 1.02 (0.14-7.45) | 1.30 (0.18-9.44) |
| **JOSAMYCIN** |  |  |  |  |  |
| **Any MCM overall** | 1.00 (0.90-1.12) | 1.01 (0.90-1.12) | 0.96 (0.85-1.10) | 0.97 (0.85-1.10) | 1.00 (0.90-1.11) |
| **Nervous system** |  |  |  |  |  |
| Severe microcephaly | 1.01 (0.48-2.14) | 0.99 (0.47-2.10) | 1.21 (0.53-2.73) | 0.93 (0.38-2.27) | 0.86 (0.41-1.82) |
| Hydrocephaly | 0.95 (0.35-2.56) | 0.95 (0.35-2.56) | 0.98 (0.31-3.08) | 1.29 (0.48-3.52) | 0.59 (0.19-1.85) |
| Spina Bifida | 2.53 (1.17-5.46) | 2.52 (1.16-5.45) | 2.46 (0.99-6.10) | 2.61 (1.05-6.52) | 1.18 (0.43-3.19) |
| Agenesis of the corpus callosum | 0.25 (0.03-1.76) | 0.24 (0.03-1.75) | 0.31 (0.04-2.26) | 0 | 0.67 (0.22-2.12) |
| **Eye anomalies** |  |  |  |  |  |
| Congenital cataract | 1.11 (0.35-3.51) | 1.09 (0.35-3.46) | 1.01 (0.25-4.12) | 1.06 (0.26-4.33) | 0.38 (0.05-2.76) |
| **Heart defects** |  |  |  |  |  |
| Atrioventricular septal defect | 1.12 (0.87-1.44) | 1.13 (0.88-1.45) | 0.93 (0.67-1.29) | 1.11 (0.83-1.49) | 1.11 (0.88-1.40) |
| Atrial septal defect | 0.97 (0.71-1.33) | 1.02 (0.75-1.40) | 1.12 (0.79-1.59) | 0.99 (0.69-1.44) | 1.06 (0.80-1.40) |
| Congenital pulmonary valve | 0.79 (0.33-1.92) | 0.79 (0.32-1.92) | 0.64 (0.20-2.00) | 0.83 (0.31-2.24) | 1.51 (0.80-2.85) |
| D-TGA | 1.21 (0.57-2.58) | 1.23 (0.58-2.63) | 0.98 (0.36-2.66) | 1.15 (0.47-2.80) | 1.15 (0.57-2.33) |
| Coarctation of aorta | 1.23 (0.63-2.39) | 1.22 (0.63-2.38) | 0.72 (0.27-1.95) | 1.34 (0.63-2.87) | 0.98 (0.49-1.99) |
| Tetralogy of Fallot | 1.20 (0.56-2.55) | 1.19 (0.56-2.54) | 1.15 (0.47-2.80) | 1.33 (0.59-3.02) | 1.03 (0.49-2.19) |
| Ventricular septal defect | 1.28 (0.47-3.48) | 1.27 (0.47-3.45) | 0.46 (0.06-3.29) | 1.90 (0.69-5.22) | 1.77 (0.83-3.78) |
| PDA as only CHD in term infants | 0.57 (0.14-2.33) | 0.60 (0.15-2.41) | 0.80 (0.20-3.25) | 0.77 (0.19-3.15) | 0.29 (0.04-2.11) |
| Hypoplastic left heart | 1.18 (0.37-3.73) | 1.17 (0.37-3.70) | 1.09 (0.27-4.48) | 1.57 (0.49-5.02) | 1.20 (0.38-3.80) |
| Aortic valve atresia/stenosis | 1.07 (0.26-4.38) | 1.04 (0.25-4.27) | 1.37 (0.33-5.64) | 0.68 (0.09-4.97) | 0.78 (0.19-3.19) |
| Double outlet right ventricle | 2.30 (0.83-6.35) | 2.24 (0.81-6.20) | 2.42 (0.75-7.80) | 2.03 (0.63-6.56) | 0.39 (0.05-2.82) |
| Pulmonary valve atresia | 0 | 0 | 0 | 0 | 1.51 (0.55-4.13) |
| **Oro-facial clefts** |  |  |  |  |  |
| Cleft lip with and without cleft palate | 0.98 (0.60-1.61) | 0.98 (0.60-1.62) | 0.86 (0.46-1.62) | 1.03 (0.59-1.78) | 0.71 (0.42-1.20) |
| Cleft palate | 0.69 (0.33-1.46) | 0.69 (0.33-1.46) | 0.68 (0.28-1.65) | 0.52 (0.19-1.40) | 0.40 (0.15-1.07) |
| **Digestive system** |  |  |  |  |  |
| Ano-rectal atresia | 1.11 (0.49-2.50) | 1.10 (0.49-2.49) | 1.26 (0.52-3.07) | 0.91 (0.34-2.47) | 0.56 (0.18-1.75) |
| Oesophageal atresia | 0.91 (0.34-2.45) | 0.90 (0.33-2.45) | 1.30 (0.48-3.52) | 0.87 (0.28-2.74) | 0.17 (0.02-1.22) |
| Diaphragmatic hernia | 0.76 (0.24-2.40) | 0.80 (0.25-2.50) | 0.76 (0.19-3.08) | 0.34 (0.05-2.48) | 0.63 (0.20-1.99) |
| Hirschrung's disease | 0.94 (0.23-3.84) | 0.93 (0.23-3.79) | 0.73 (0.10-5.29) | 1.17 (0.28-4.79) | 1.55 (0.57-4.24) |
| Atresia or stenosis of intestine | 1.41 (0.44-4.49) | 1.40 (0.44-4.45) | 1.87 (0.58-5.98) | 1.16 (0.28-4.78) | 0.99 (0.24-4.05) |
| Anomalies of intestinal fixation | 0 | 0 | 0 | 0 | 1.74 (0.54-5.56) |
| **Abdominal wall defects** |  |  |  |  |  |
| Omphalocele | 0.67 (0.17-2.74) | 0.69 (0.17-2.81) | 0.48 (0.07-3.42) | 0.94 (0.23-3.83) | 1.92 (0.84-4.38) |
| Gastroschisis | 0.97 (0.24-3.96) | 1.02 (0.25-4.17) | 0.66 (0.09-4.78) | 0.56 (0.08-4.08) | 1.40 (0.51-3.81) |
| **Anomalies of kidney and urinary tract** |  |  |  |  |  |
| Hydronephrosis | 0.88 (0.61-1.28) | 0.88 (0.61-1.28) | 0.96 (0.63-1.47) | 0.67 (0.41-1.11) | 1.15 (0.83-1.60) |
| Unilateral Renal Agenesis | 0.56 (0.21-1.50) | 0.56 (0.21-1.51) | 0.61 (0.19-1.91) | 0.72 (0.27-1.93) | 0.80 (0.35-1.79) |
| Renal Dysplasia | 1.26 (0.59-2.69) | 1.27 (0.60-2.70) | 1.42 (0.63-3.22) | 1.44 (0.63-3.26) | 0.97 (0.40-2.36) |
| Horseshoe kidney | 1.06 (0.44-2.59) | 1.09 (0.45-2.66) | 1.19 (0.44-3.23) | 0.57 (0.14-2.32) | 0.67 (0.21-2.11) |
| Posterior urethral valve | 0.93 (0.23-3.78) | 0.88 (0.22-3.59) | 0.61 (0.08-4.44) | 0.59 (0.08-4.24) | 2.12 (0.93-4.84) |
| **Genital anomalies** |  |  |  |  |  |
| Hypospadias | 0.76 (0.54-1.06) | 0.75 (0.53-1.04) | 0.73 (0.49-1.08) | 0.76 (0.52-1.12) | 1.04 (0.78-1.38) |
| **Limb anomalies** |  |  |  |  |  |
| Club foot | 1.05 (0.67-1.67) | 1.07 (0.68-1.70) | 0.87 (0.48-1.58) | 1.10 (0.66-1.84) | 0.89 (0.55-1.44) |
| Polydactyly | 1.55 (1.04-2.29) | 1.50 (1.01-2.23) | 1.28 (0.79-2.07) | 1.72 (1.12-2.64) | 0.77 (0.47-1.27) |
| Hip dislocation | 1.23 (0.74-2.06) | 1.26 (0.75-2.12) | 1.31 (0.73-2.32) | 1.23 (0.67-2.24) | 1.42 (0.91-2.22) |
| Syndactyly | 2.32 (0.93-5.74) | 2.23 (0.90-5.54) | 0.64 (0.09-4.64) | 2.01 (0.62-6.49) | 1.50 (0.55-4.10) |
| Limb reduction defects | 1.05 (0.43-2.56) | 1.07 (0.44-2.62) | 1.47 (0.60-3.60) | 0.87 (0.28-2.74) | 0.57 (0.18-1.79) |
| **Other anomalies** |  |  |  |  |  |
| Craniosynostoses | 0.98 (0.48-1.98) | 1.00 (0.50-2.03) | 1.20 (0.56-2.55) | 1.23 (0.58-2.62) | 0.85 (0.42-1.72) |
| Vascular disruption anomalies | 0.85 (0.35-2.06) | 0.83 (0.34-2.02) | 0.89 (0.33-2.40) | 0.61 (0.19-1.90) | 1.25 (0.64-2.43) |
| Laterality anomalies | 0.95 (0.30-3.00) | 0.94 (0.30-2.98) | 0.91 (0.22-3.71) | 0.37 (0.05-2.65) | 0.92 (0.29-2.90) |
| Situs inversus | 1.63 (0.51-5.21) | 1.62 (0.51-5.20) | 1.47 (0.36-6.05) | 0.61 (0.08-4.45) | 0.54 (0.07-3.90) |
| **ERYTHROMYCIN** |  |  |  |  |  |
| **Any MCM overall** | 1.01 (0.85-1.20) | 1.04 (0.88-1.24) | 1.06 (0.86-1.30) | 1.00 (0.83-1.22) | 0.94 (0.81-1.09) |
| **Nervous system** |  |  |  |  |  |
| Severe microcephaly | 1.48 (0.55-3.97) | 1.44 (0.54-3.89) | 1.11 (0.28-4.50) | 1.29 (0.41-4.04) | 0.68 (0.22-2.13) |
| Hydrocephaly | 1.82 (0.58-5.71) | 1.88 (0.60-5.93) | 2.83 (0.90-8.93) | 1.61 (0.40-6.53) | 0.35 (0.05-2.52) |
| Spina Bifida | 0.92 (0.13-6.63) | 1.02 (0.14-7.34) | 0 | 0 | 0.56 (0.08-4.05) |
| Agenesis of the corpus callosum | 1.26 (0.31-5.10) | 1.32 (0.33-5.34) | 0.93 (0.13-6.65) | 1.73 (0.42-7.02) | 1.30 (0.41-4.07) |
| **Eye anomalies** |  |  |  |  |  |
| Congenital cataract | 0 | 0 | 0 | 0 | 2.07 (0.65-6.59) |
| **Heart defects** |  |  |  |  |  |
| Atrioventricular septal defect | 1.30 (0.89-1.88) | 1.33 (0.92-1.94) | 1.49 (0.97-2.29) | 1.10 (0.70-1.74) | 0.96 (0.68-1.36) |
| Atrial septal defect | 0.85 (0.50-1.44) | 0.99 (0.58-1.67) | 0.73 (0.35-1.55) | 1.05 (0.59-1.86) | 0.76 (0.48-1.21) |
| Congenital pulmonary valve | 1.22 (0.39-3.80) | 1.25 (0.40-3.91) | 1.80 (0.57-5.65) | 1.53 (0.49-4.80) | 1.13 (0.42-3.05) |
| D-TGA | 1.77 (0.66-4.78) | 1.89 (0.70-5.11) | 2.05 (0.65-6.46) | 2.18 (0.80-5.91) | 0.58 (0.14-2.33) |
| Coarctation of aorta | 2.10 (0.93-4.72) | 2.11 (0.94-4.76) | 3.05 (1.35-6.90) | 2.77 (1.22-6.27) | 0 |
| Tetralogy of Fallot | 1.76 (0.65-4.73) | 1.73 (0.64-4.67) | 1.94 (0.62-6.10) | 1.04 (0.26-4.21) | 1.29 (0.53-3.13) |
| Ventricular septal defect | 0.82 (0.11-5.87) | 0.86 (0.12-6.21) | 1.29 (0.18-9.33) | 1.14 (0.16-8.24) | 0.97 (0.24-3.92) |
| PDA as only CHD in term infants | 1.47 (0.36-5.96) | 1.52 (0.37-6.17) | 0 | 0.88 (0.12-6.36) | 1.09 (0.27-4.44) |
| Hypoplastic left heart | 1.01 (0.14-7.23) | 1.10 (0.15-7.96) | 0 | 1.30 (0.18-9.41) | 0 |
| Aortic valve atresia/stenosis | 0 | 0 | 0 | 0 | 0 |
| Double outlet right ventricle | 2.94 (0.72-12.06) | 3.01 (0.73-12.45) | 4.52 (1.08-18.84) | 3.53 (0.85-14.64) | 0 |
| Pulmonary valve atresia | 0 | 0 | 0 | 0 | 0.71 (0.10-5.12) |
| **Oro-facial clefts** |  |  |  |  |  |
| Cleft lip with and without cleft palate | 1.10 (0.52-2.31) | 1.12 (0.53-2.36) | 0.96 (0.36-2.58) | 1.14 (0.51-2.56) | 0.85 (0.44-1.65) |
| Cleft palate | 0.51 (0.13-2.03) | 0.51 (0.13-2.05) | 0.38 (0.05-2.69) | 0.62 (0.15-2.49) | 0.59 (0.19-1.85) |
| **Digestive system** |  |  |  |  |  |
| Ano-rectal atresia | 0.95 (0.23-3.81) | 0.98 (0.24-3.95) | 1.42 (0.35-5.77) | 1.16 (0.29-4.71) | 1.04 (0.33-3.25) |
| Oesophageal atresia | 0 | 0 | 0 | 0 | 0 |
| Diaphragmatic hernia | 0.65 (0.09-4.66) | 0.70 (0.10-5.01) | 1.14 (0.16-8.16) | 0.84 (0.12-6.03) | 0 |
| Hirschrung's disease | 0 | 0 | 0 | 0 | 0 |
| Atresia or stenosis of intestine | 1.20 (0.17-8.67) | 1.18 (0.16-8.54) | 1.72 (0.24-12.46) | 1.36 (0.19-9.87) | 2.79 (0.88-8.87) |
| Anomalies of intestinal fixation | 2.94 (0.72-12.06) | 3.25 (0.79-13.39) | 2.35 (0.32-17.07) | 1.87 (0.26-13.66) | 0 |
| **Abdominal wall defects** |  |  |  |  |  |
| Omphalocele | 0.86 (0.12-6.19) | 0.98 (0.14-7.04) | 1.42 (0.20-10.21) | 1.18 (0.16-8.51) | 0.63 (0.09-4.52) |
| Gastroschisis | 2.48 (0.61-10.14) | 2.56 (0.62-10.50) | 3.65 (0.89-15.06) | 1.30 (0.18-9.40) | 2.44 (0.89-6.67) |
| **Anomalies of kidney and urinary tract** |  |  |  |  |  |
| Hydronephrosis | 0.97 (0.55-1.71) | 0.99 (0.56-1.74) | 1.35 (0.74-2.44) | 1.00 (0.54-1.88) | 0.68 (0.38-1.23) |
| Unilateral Renal Agenesis | 0.71 (0.18-2.87) | 0.76 (0.19-3.07) | 0.58 (0.08-4.18) | 0.92 (0.23-3.69) | 0.74 (0.24-2.30) |
| Renal Dysplasia | 0.92 (0.23-3.72) | 0.95 (0.24-3.84) | 1.39 (0.34-5.61) | 0.57 (0.08-4.07) | 0.72 (0.18-2.92) |
| Horseshoe kidney | 1.09 (0.27-4.39) | 1.11 (0.27-4.50) | 0 | 0.67 (0.09-4.81) | 0.43 (0.06-3.04) |
| Posterior urethral valve | 0 | 0 | 0 | 0 | 0.69 (0.10-4.99) |
| **Genital anomalies** |  |  |  |  |  |
| Hypospadias | 0.83 (0.50-1.38) | 0.81 (0.49-1.35) | 0.80 (0.43-1.50) | 0.78 (0.44-1.38) | 1.37 (0.97-1.92) |
| **Limb anomalies** |  |  |  |  |  |
| Club foot | 0.85 (0.38-1.91) | 0.90 (0.40-2.02) | 0.92 (0.34-2.45) | 0.71 (0.27-1.90) | 0.98 (0.52-1.83) |
| Polydactyly | 0.91 (0.41-2.04) | 0.85 (0.38-1.90) | 1.18 (0.53-2.64) | 0.68 (0.25-1.82) | 1.38 (0.85-2.24) |
| Hip dislocation | 0.84 (0.31-2.25) | 0.86 (0.32-2.32) | 0.90 (0.29-2.80) | 1.06 (0.40-2.86) | 1.12 (0.56-2.25) |
| Syndactyly | 1.19 (0.16-8.54) | 1.15 (0.16-8.35) | 1.80 (0.25-13.05) | 1.63 (0.22-11.87) | 0.66 (0.09-4.76) |
| Limb reduction defects | 0.54 (0.08-3.83) | 0.59 (0.08-4.20) | 0.87 (0.12-6.26) | 0.76 (0.11-5.42) | 0.35 (0.05-2.50) |
| **Other anomalies** |  |  |  |  |  |
| Craniosynostoses | 1.25 (0.47-3.36) | 1.36 (0.51-3.65) | 1.53 (0.49-4.78) | 1.71 (0.63-4.60) | 0.42 (0.11-1.70) |
| Vascular disruption anomalies | 1.74 (0.64-4.67) | 1.78 (0.66-4.80) | 2.53 (0.94-6.85) | 1.51 (0.48-4.74) | 2.04 (1.00-4.13) |
| Laterality anomalies | 1.62 (0.40-6.57) | 1.61 (0.40-6.57) | 1.29 (0.18-9.30) | 0.88 (0.12-6.36) | 0 |
| Situs inversus | 1.39 (0.19-10.06) | 1.43 (0.20-10.36) | 2.10 (0.29-15.31) | 1.54 (0.21-11.23) | 0 |
