# Supplementary material for: First-trimester exposure to macrolides and risk of major congenital malformations compared with amoxicillin: A French nationwide cohort study
Source: PLoS Med. 2025 Apr 15;22(4):e1004576. doi: 10.1371/journal.pmed.1004576 (PMC12021278; doi:10.1371/journal.pmed.1004576)
Supplement: S11 Table — (DOCX) [file pmed.1004576.s012.docx]

**S11 Table.** Supplementary analysis - Adjusted relative risks of any MCM and 42 selected individual MCMs (sorted by the most common to the least common MCMs in the organ-specific groups) in pregnancies exposed to macrolides overall during the first trimester compared with the cephalosporin exposure group

We conducted a supplementary analysis in which the comparator group was pregnancies exposed to cephalosporin antibiotics (ATC group J01DB, J01DC, J01DD, and J01DE) during the first trimester. We included 165,308 pregnancies in the macrolide exposure group and 205,074 in the cephalosporin exposure group (same inclusion and exclusion criteria as those in the main analysis). After the propensity score, two pregnancies were excluded from the macrolide exposure group and one from the cephalosporin exposure group due to trimming. The table below presents the adjusted relative risks of any MCM and 42 selected individual MCMs for the macrolide exposure compared to the cephalosporin exposure.

|  | **N exposed events (After Propensity score)** | |  |
| --- | --- | --- | --- |
| **Outcome** | **Macrolide exposure** | **Cephalosporin exposure** | **Adjusted RR** |
|  | **(N total=165,306)** | **(N total=205,073)** | **(95% CI)** |
| **Any MCM overall** | 2875 | 3530 | 1.01 (0.96-1.06) |
| **Nervous system** |  |  |  |
| Severe microcephaly | 61 | 63 | 1.17 (0.82-1.67) |
| Hydrocephaly | 43 | 51 | 1.05 (0.70-1.58) |
| Spina Bifida | 41 | 35 | 1.52 (0.98-2.35) |
| Agenesis of the corpus callosum | 30 | 36 | 1.08 (0.67-1.74) |
| **Eye anomalies** |  |  |  |
| Congenital cataract | 24 | 22 | 1.29 (0.74-2.27) |
| **Heart defects** |  |  |  |
| Atrioventricular septal defect | 517 | 569 | 1.13 (1.00-1.27) |
| Atrial septal defect | 341 | 459 | 0.93 (0.81-1.07) |
| Congenital pulmonary valve | 71 | 70 | 1.30 (0.95-1.78) |
| D-TGA | 66 | 70 | 1.20 (0.87-1.66) |
| Coarctation of aorta | 62 | 56 | 1.32 (0.92-1.91) |
| Tetralogy of Fallot | 50 | 61 | 0.97 (0.67-1.41) |
| Ventricular septal defect | 30 | 28 | 1.29 (0.77-2.15) |
| PDA as only CHD in term infants | 26 | 34 | 0.98 (0.61-1.59) |
| Hypoplastic left heart | 25 | 24 | 1.42 (0.85-2.35) |
| Aortic valve atresia/stenosis | 18 | 24 | 1.02 (0.57-1.83) |
| Double outlet right ventricle | 17 | 21 | 0.97 (0.52-1.83) |
| Pulmonary valve atresia | 19 | 21 | 1.15 (0.64-2.07) |
| **Oro-facial clefts** |  |  |  |
| Cleft lip with and without cleft palate | 122 | 185 | 0.82 (0.66-1.03) |
| Cleft palate | 71 | 100 | 0.85 (0.63-1.15) |
| **Digestive system** |  |  |  |
| Ano-rectal atresia | 53 | 57 | 1.10 (0.76-1.60) |
| Oesophageal atresia | 37 | 54 | 0.81 (0.54-1.23) |
| Diaphragmatic hernia | 27 | 40 | 0.86 (0.53-1.41) |
| Hirschrung's disease | 20 | 30 | 0.81 (0.46-1.43) |
| Atresia or stenosis of intestine | 21 | 24 | 1.21 (0.69-2.12) |
| Anomalies of intestinal fixation | 14 | 27 | 0.66 (0.35-1.24) |
| **Abdominal wall defects** |  |  |  |
| Omphalocele | 21 | 29 | 0.90 (0.52-1.55) |
| Gastroschisis | 22 | 42 | 0.65 (0.38-1.09) |
| **Anomalies of kidney and urinary tract** |  |  |  |
| Hydronephrosis | 244 | 331 | 0.93 (0.79-1.09) |
| Unilateral Renal Agenesis | 57 | 84 | 0.86 (0.62-1.20) |
| Renal Dysplasia | 48 | 46 | 1.24 (0.83-1.87) |
| Horseshoe kidney | 33 | 45 | 0.91 (0.59-1.40) |
| Posterior urethral valve | 20 | 22 | 1.14 (0.64-2.03) |
| **Genital anomalies** |  |  |  |
| Hypospadias | 417 | 466 | 1.10 (0.97-1.26) |
| **Limb anomalies** |  |  |  |
| Club foot | 151 | 182 | 1.01 (0.82-1.25) |
| Polydactyly | 161 | 183 | 1.07 (0.87-1.31) |
| Hip dislocation | 127 | 142 | 1.15 (0.91-1.46) |
| Syndactyly | 32 | 29 | 1.41 (0.86-2.29) |
| Limb reduction defects | 29 | 46 | 0.79 (0.50-1.27) |
| **Other anomalies** |  |  |  |
| Craniosynostoses | 66 | 105 | 0.76 (0.56-1.02) |
| Vascular disruption anomalies | 49 | 91 | 0.69 (0.49-0.97) |
| Laterality anomalies | 30 | 34 | 1.12 (0.69-1.82) |
| Situs inversus | 20 | 22 | 1.18 (0.65-2.16) |
