# Supplementary material for: First-trimester exposure to macrolides and risk of major congenital malformations compared with amoxicillin: A French nationwide cohort study
Source: PLoS Med. 2025 Apr 15;22(4):e1004576. doi: 10.1371/journal.pmed.1004576 (PMC12021278; doi:10.1371/journal.pmed.1004576)
Supplement: S12 Table — (DOCX) [file pmed.1004576.s013.docx]

**S12 Table.** Supplementary analysis - Adjusted relative risks of any MCM and 42 selected individual MCMs (sorted by the most common to the least common MCMs in the organ-specific groups) in pregnancies exposed to macrolides overall during the first trimester compared with the unexposed group

|  | **N exposed events (After Propensity score)** | |  |
| --- | --- | --- | --- |
| **Outcome** | **Macrolide exposure** | **Unexposed** | **Adjusted RR** |
|  | **(N total=140,708)** | **(N total=5,850,899)** | **(95% CI)** |
| **Any MCM overall** | 2432 | 95721 | 1.02 (0.98-1.06) |
| **Nervous system** |  |  |  |
| Severe microcephaly | 52 | 1839 | 1.15 (0.87-1.51) |
| Hydrocephaly | 43 | 1254 | 1.29 (0.95-1.76) |
| Spina Bifida | 36 | 845 | 1.77 (1.27-2.48) |
| Agenesis of the corpus callosum | 24 | 1279 | 0.77 (0.51-1.15) |
| **Eye anomalies** |  |  |  |
| Congenital cataract | 20 | 771 | 1.03 (0.66-1.60) |
| **Heart defects** |  |  |  |
| Atrioventricular septal defect | 441 | 15480 | 1.13 (1.02-1.24) |
| Atrial septal defect | 289 | 11318 | 0.98 (0.87-1.11) |
| Congenital pulmonary valve | 58 | 1648 | 1.39 (1.07-1.81) |
| D-TGA | 51 | 1868 | 1.09 (0.82-1.44) |
| Coarctation of aorta | 48 | 1935 | 0.98 (0.73-1.30) |
| Tetralogy of Fallot | 42 | 1800 | 0.95 (0.70-1.29) |
| Ventricular septal defect | 25 | 1031 | 0.98 (0.66-1.46) |
| PDA as only CHD in term infants | 22 | 806 | 1.13 (0.74-1.73) |
| Hypoplastic left heart | 22 | 667 | 1.26 (0.82-1.94) |
| Aortic valve atresia/stenosis | 18 | 561 | 1.27 (0.79-2.04) |
| Double outlet right ventricle | 16 | 556 | 1.10 (0.67-1.82) |
| Pulmonary valve atresia | 14 | 581 | 0.97 (0.57-1.65) |
| **Oro-facial clefts** |  |  |  |
| Cleft lip with and without cleft palate | 102 | 4828 | 0.86 (0.71-1.05) |
| Cleft palate | 59 | 2450 | 0.98 (0.76-1.27) |
| **Digestive system** |  |  |  |
| Ano-rectal atresia | 46 | 1492 | 1.26 (0.94-1.69) |
| Oesophageal atresia | 34 | 1321 | 1.03 (0.73-1.46) |
| Diaphragmatic hernia | 22 | 1228 | 0.71 (0.46-1.08) |
| Hirschrung's disease | 18 | 640 | 1.12 (0.70-1.80) |
| Atresia or stenosis of intestine | 17 | 539 | 1.26 (0.77-2.05) |
| Anomalies of intestinal fixation | 14 | 546 | 1.02 (0.60-1.75) |
| **Abdominal wall defects** |  |  |  |
| Omphalocele | 21 | 853 | 0.93 (0.60-1.44) |
| Gastroschisis | 17 | 753 | 0.94 (0.58-1.53) |
| **Anomalies of kidney and urinary tract** |  |  |  |
| Hydronephrosis | 209 | 8776 | 0.95 (0.83-1.10) |
| Unilateral Renal Agenesis | 47 | 2214 | 0.89 (0.66-1.19) |
| Renal Dysplasia | 40 | 1477 | 1.08 (0.79-1.48) |
| Horseshoe kidney | 26 | 1413 | 0.77 (0.52-1.13) |
| Posterior urethral valve | 18 | 659 | 1.13 (0.70-1.80) |
| **Genital anomalies** |  |  |  |
| Hypospadias | 346 | 13251 | 1.06 (0.95-1.18) |
| **Limb anomalies** |  |  |  |
| Club foot | 131 | 5017 | 1.06 (0.89-1.26) |
| Polydactyly | 127 | 5995 | 0.86 (0.72-1.03) |
| Hip dislocation | 109 | 4198 | 1.08 (0.89-1.31) |
| Syndactyly | 31 | 757 | 1.57 (1.09-2.26) |
| Limb reduction defects | 27 | 1301 | 0.83 (0.56-1.21) |
| **Other anomalies** |  |  |  |
| Craniosynostoses | 55 | 2336 | 0.96 (0.74-1.26) |
| Vascular disruption anomalies | 43 | 1765 | 0.95 (0.70-1.29) |
| Laterality anomalies | 27 | 788 | 1.40 (0.95-2.06) |
| Situs inversus | 18 | 426 | 1.73 (1.08-2.78) |
