# Supplementary material for: First-trimester exposure to macrolides and risk of major congenital malformations compared with amoxicillin: A French nationwide cohort study
Source: PLoS Med. 2025 Apr 15;22(4):e1004576. doi: 10.1371/journal.pmed.1004576 (PMC12021278; doi:10.1371/journal.pmed.1004576)
Supplement: S13 Table — (DOCX) [file pmed.1004576.s014.docx]

**S13 Table.** Supplementary analysis - Adjusted relative risks of 12 organ-specific MCM groups in pregnancies exposed to macrolides during the first trimester compared with amoxicillin

|  | **Macrolides** | | **Azithromycin** | | **Spiramycin** | | **Clarithromycin** | | **Roxithromycin** | | **Josamycin** | | **Erythromycin** | |
| --- | --- | --- | --- | --- | --- | --- | --- | --- | --- | --- | --- | --- | --- | --- |
| **Outcome (Organ-specific MCM groups)** | **N** | **aRR (95%CI)** | **N** | **aRR (95%CI)** | **N** | **aRR (95%CI)** | **N** | **aRR (95%CI)** | **N** | **aRR (95%CI)** | **N** | **aRR (95%CI)** | **N** | **aRR (95%CI)** |
| Heart | 809 | 1.02 (0.94-1.10) | 249 | 1.00 (0.88-1.15) | 195 | 1.02 (0.88-1.18) | 114 | 0.93 (0.77-1.12) | 114 | 1.09 (0.90-1.32) | 115 | 1.10 (0.91-1.32) | 49 | 1.25 (0.94-1.66) |
| Limb | 414 | 1.06 (0.94-1.18) | 133 | 1.09 (0.91-1.31) | 89 | 0.93 (0.75-1.16) | 59 | 0.99 (0.76-1.30) | 57 | 1.12 (0.86-1.47) | 69 | 1.31 (1.03-1.67) | 18 | 0.88 (0.55-1.40) |
| Urinary system | 331 | 0.93 (0.82-1.05) | 91 | 0.83 (0.67-1.03) | 103 | 1.14 (0.93-1.39) | 38 | 0.71 (0.51-0.98) | 46 | 1.03 (0.77-1.39) | 47 | 0.95 (0.71-1.27) | 17 | 0.90 (0.56-1.45) |
| Genital defects | 355 | 1.01 (0.90-1.14) | 113 | 1.05 (0.86-1.28) | 78 | 0.88 (0.70-1.11) | 64 | 1.21 (0.93-1.56) | 54 | 1.17 (0.89-1.55) | 36 | 0.75 (0.54-1.05) | 16 | 0.85 (0.52-1.39) |
| Digestive system | 152 | 0.97 (0.81-1.16) | 50 | 1.02 (0.76-1.37) | 40 | 1.02 (0.74-1.41) | 18 | 0.77 (0.48-1.23) | 24 | 1.19 (0.79-1.80) | 18 | 0.84 (0.53-1.35) | 6 | 0.74 (0.33-1.66) |
| Nervous system | 155 | 1.14 (0.96-1.37) | 52 | 1.20 (0.89-1.61) | 37 | 1.14 (0.82-1.60) | 24 | 1.16 (0.76-1.76) | 18 | 1.00 (0.62-1.62) | 18 | 0.97 (0.61-1.55) | 11 | 1.58 (0.87-2.86) |
| Orofacial clefts | 161 | 0.83 (0.70-0.99) | 46 | 0.80 (0.59-1.09) | 42 | 0.83 (0.61-1.14) | 30 | 1.04 (0.71-1.51) | 14 | 0.55 (0.32-0.94) | 23 | 0.87 (0.57-1.32) | 9 | 0.88 (0.46-1.71) |
| Musculoskeletal system | 37 | 1.15 (0.80-1.66) | 9 | 0.94 (0.48-1.86) | 9 | 1.00 (0.51-1.97) | 8 | 1.78 (0.86-3.71) | 5 | 1.30 (0.53-3.20) | 3 | 0.88 (0.33-2.38) | 3 | 1.77 (0.56-5.56) |
| Eye defects | 36 | 0.94 (0.65-1.36) | 10 | 0.83 (0.43-1.62) | 10 | 1.11 (0.58-2.11) | 2 | 0.34 (0.08-1.39) | 6 | 1.09 (0.47-2.55) | 7 | 1.35 (0.63-2.89) | 1 | 0.53 (0.07-3.80) |
| Respiratory system | 12 | 1.40 (0.72-2.72) | 5 | 1.60 (0.60-4.25) | 2 | 1.02 (0.24-4.26) | 1 | 0.70 (0.09-5.32) | 1 | 0.94 (0.13-6.94) | 2 | 1.81 (0.43-7.55) | 1 | 2.49 (0.34-18.30) |
| Ear | 8 | 1.00 (0.45-2.23) | 3 | 1.35 (0.40-4.58) | 3 | 1.53 (0.47-5.03) | 0 | N/A | 1 | 0.90 (0.12-7.06) | 1 | 0.95 (0.13-6.96) | 0 | N/A |
| Other defects | 126 | 0.99 (0.81-1.20) | 44 | 1.10 (0.80-1.51) | 29 | 0.90 (0.62-1.31) | 19 | 1.00 (0.63-1.60) | 13 | 0.77 (0.44-1.35) | 15 | 0.92 (0.56-1.51) | 10 | 1.53 (0.82-2.86) |
| *N = No. events in the macrolide exposure group (after propensity score adjustment)* | | | | | | | | | | | | | | |
