# Supplementary material for: First-trimester exposure to macrolides and risk of major congenital malformations compared with amoxicillin: A French nationwide cohort study
Source: PLoS Med. 2025 Apr 15;22(4):e1004576. doi: 10.1371/journal.pmed.1004576 (PMC12021278; doi:10.1371/journal.pmed.1004576)
Supplement: S14 Table — (DOCX) [file pmed.1004576.s015.docx]

**S14 Table**. Post-hoc analysis - Adjusted relative risks of spina bifida and syndactyly across baseline characteristic strata

|  | **Spina bifida** | | **Syndactyly** | |
| --- | --- | --- | --- | --- |
| **Baseline characteristic** | **N exposed events/N total (After PS)** | **Adjusted RR**  **(95% CI)** | **N exposed events/N total**  **(After PS)** | **Adjusted RR**  **(95% CI)** |
| **Maternal age** |  |  |  |  |
| $\leq$ 19 (young) | 2/4,584 | 2.47 (0.39-15.73) | 0/4,584 | N/A |
| 20-34 (normal) | 28/109,889 | 1.70 (1.09-2.66) | 25/109,889 | 1.55 (0.95-2.53) |
| $\geq$ 35 (old) | 6/26,232 | 2.40 (0.85-6.78) | 6/26,232 | 2.85 (0.94-8.66) |
| **Reimbursed folic acid supplementation** |  |  |  |  |
| Yes | 7/57,437 | 0.92 (0.40-2.10) | 19/57,437 | 2.42 (1.30-4.48) |
| No | 29/83,267 | 2.36 (1.49-3.75) | 12/83,267 | 1.11 (0.56-2.17) |
| **Assisted reproduction** |  |  |  |  |
| Yes | 2/5,929 | 6.49 (0.59-71.59) | 1/5,929 | 3.28 (0.30-36.18) |
| No | 34/134,777 | 1.75 (1.16-2.62) | 30/134,777 | 1.71 (1.09-2.68) |
| **Smoking-related conditions** |  |  |  |  |
| Yes | 33/125,158 | 2.10 (1.38-3.22) | 27/125,158 | 1.67 (1.04-2.68) |
| No | 3/15,546 | 0.73 (0.21-2.54) | 4/15,546 | 1.62 (0.48-5.53) |
| **Alcohol-related conditions** |  |  |  |  |
| Yes | 1/1,122 | 1.45 (0.15-14.26) | 0/1,122 | N/A |
| No | 35/139,683 | 1.82 (1.22-2.73) | 31/139,683 | 1.65 (1.06-2.58) |
| **Substance use disorders** |  |  |  |  |
| Yes | 0/767 | N/A | 0/767 | N/A |
| No | 36/139,937 | 1.82 (1.22-2.70) | 31/139,937 | 1.65 (1.06-2.58) |
| **Antihypertensive drug use** |  |  |  |  |
| Yes | 1/2,230 | N/A | 0/2,230 | N/A |
| No | 35/138,474 | 1.77 (1.18-2.64) | 31/138,474 | 1.69 (1.08-2.65) |
| **Obesity-related hospital discharge or long-term disease diagnoses** |  |  |  |  |
| Yes | 5/8,444 | 1.77 (0.62-5.05) | 4/8,444 | 5.22 (1.25-21.84) |
| No | 31/132,260 | 1.83 (1.19-2.81) | 27/132,260 | 1.51 (0.94-2.41) |
| **Antidiabetic drug use or diabetes-related hospital discharge/long-term disease diagnoses** |  |  |  |  |
| Yes | 3/1,196 | 3.46 (0.66-18.01) | 0/1,196 | N/A |
| No | 33/139,510 | 1.73 (1.14-2.61) | 31/139,510 | 1.65 (1.05-2.59) |
