# Supplementary material for: First-trimester exposure to macrolides and risk of major congenital malformations compared with amoxicillin: A French nationwide cohort study
Source: PLoS Med. 2025 Apr 15;22(4):e1004576. doi: 10.1371/journal.pmed.1004576 (PMC12021278; doi:10.1371/journal.pmed.1004576)
Supplement: S15 Table — (DOCX) [file pmed.1004576.s016.docx]

**S15 Table**. Post-hoc analysis - Results from the main analysis when applying propensity score overlap weights

|  | **N exposed events** | |  |
| --- | --- | --- | --- |
| **Outcome** | **Macrolide exposure** | **Amoxicillin exposure** | **Adjusted RR** |
|  | **(N total=140,708)** | **(N total=592,652)** | **(95% CI)** |
| **Any MCM overall** | 2432 | 10176 | 1.00 (0.96-1.05) |
| **Nervous system** |  |  |  |
| Severe microcephaly | 52 | 215 | 1.03 (0.76-1.40) |
| Hydrocephaly | 43 | 131 | 1.34 (0.95-1.91) |
| Spina Bifida | 36 | 86 | 1.77 (1.19-2.63) |
| Agenesis of the corpus callosum | 24 | 126 | 0.80 (0.51-1.24) |
| **Eye anomalies** |  |  |  |
| Congenital cataract | 20 | 84 | 1.03 (0.63-1.69) |
| **Heart defects** |  |  |  |
| Atrioventricular septal defect | 441 | 1715 | 1.07 (0.96-1.19) |
| Atrial septal defect | 289 | 1305 | 0.93 (0.81-1.05) |
| Congenital pulmonary valve | 58 | 196 | 1.21 (0.90-1.63) |
| D-TGA | 51 | 179 | 1.24 (0.90-1.70) |
| Coarctation of aorta | 48 | 227 | 0.90 (0.66-1.23) |
| Tetralogy of Fallot | 42 | 181 | 0.99 (0.70-1.39) |
| Ventricular septal defect | 25 | 97 | 1.05 (0.67-1.65) |
| PDA as only CHD in term infants | 22 | 108 | 0.85 (0.53-1.35) |
| Hypoplastic left heart | 22 | 79 | 1.26 (0.78-2.03) |
| Aortic valve atresia/stenosis | 18 | 58 | 1.31 (0.77-2.24) |
| Double outlet right ventricle | 16 | 54 | 1.37 (0.78-2.42) |
| Pulmonary valve atresia | 14 | 71 | 0.80 (0.45-1.42) |
| **Oro-facial clefts** |  |  |  |
| Cleft lip with and without cleft palate | 102 | 507 | 0.86 (0.70-1.07) |
| Cleft palate | 59 | 314 | 0.79 (0.60-1.05) |
| **Digestive system** |  |  |  |
| Ano-rectal atresia | 46 | 168 | 1.17 (0.84-1.63) |
| Oesophageal atresia | 34 | 137 | 1.01 (0.69-1.48) |
| Diaphragmatic hernia | 22 | 122 | 0.79 (0.50-1.26) |
| Hirschrung's disease | 18 | 66 | 1.22 (0.72-2.07) |
| Atresia or stenosis of intestine | 17 | 66 | 1.12 (0.65-1.93) |
| Anomalies of intestinal fixation | 14 | 54 | 1.08 (0.59-1.96) |
| **Abdominal wall defects** |  |  |  |
| Omphalocele | 21 | 92 | 0.94 (0.58-1.51) |
| Gastroschisis | 17 | 64 | 1.16 (0.67-1.99) |
| **Anomalies of kidney and urinary tract** |  |  |  |
| Hydronephrosis | 209 | 985 | 0.91 (0.78-1.06) |
| Unilateral Renal Agenesis | 47 | 223 | 0.92 (0.67-1.27) |
| Renal Dysplasia | 40 | 172 | 1.00 (0.71-1.42) |
| Horseshoe kidney | 26 | 146 | 0.79 (0.51-1.20) |
| Posterior urethral valve | 18 | 67 | 1.18 (0.69-2.00) |
| **Genital anomalies** |  |  |  |
| Hypospadias | 346 | 1437 | 0.99 (0.88-1.11) |
| **Limb anomalies** |  |  |  |
| Club foot | 131 | 559 | 1.00 (0.82-1.21) |
| Polydactyly | 127 | 522 | 1.05 (0.87-1.28) |
| Hip dislocation | 109 | 378 | 1.18 (0.95-1.47) |
| Syndactyly | 31 | 67 | 1.80 (1.16-2.78) |
| Limb reduction defects | 27 | 148 | 0.76 (0.50-1.16) |
| **Other anomalies** |  |  |  |
| Craniosynostoses | 55 | 254 | 0.90 (0.67-1.22) |
| Vascular disruption anomalies | 43 | 183 | 0.99 (0.71-1.39) |
| Laterality anomalies | 27 | 98 | 1.20 (0.78-1.84) |
| Situs inversus | 18 | 57 | 1.39 (0.82-2.38) |
