# Supplementary material for: First-trimester exposure to macrolides and risk of major congenital malformations compared with amoxicillin: A French nationwide cohort study
Source: PLoS Med. 2025 Apr 15;22(4):e1004576. doi: 10.1371/journal.pmed.1004576 (PMC12021278; doi:10.1371/journal.pmed.1004576)
Supplement: S16 Table — (DOCX) [file pmed.1004576.s017.docx]

**S6 Table**. Post-hoc analysis – Statistical significance of results from the main analyses with the Benjamini-Hochberg (BH) procedure (5% false discovery rate)

| **Individual MCMs** | **P-value** | **Rank** | **BH threshold** | **Significance** |
| --- | --- | --- | --- | --- |
| Spina Bifida | 0.00318 | 1 | 0.00119 | Not significant |
| Syndactyly | 0.02789 | 2 | 0.00238 | Not significant |
| Hydrocephaly | 0.06240 | 3 | 0.00357 | Not significant |
| Hip dislocation | 0.10329 | 4 | 0.00476 | Not significant |
| Cleft palate | 0.11256 | 5 | 0.00595 | Not significant |
| Cleft lip with and without cleft palate | 0.15806 | 6 | 0.00714 | Not significant |
| Situs inversus | 0.16857 | 7 | 0.00833 | Not significant |
| Limb reduction defects | 0.16947 | 8 | 0.00952 | Not significant |
| Atrioventricular septal defect | 0.17746 | 9 | 0.01071 | Not significant |
| D-TGA | 0.19174 | 10 | 0.01190 | Not significant |
| Atrial septal defect | 0.20645 | 11 | 0.01310 | Not significant |
| Horseshoe kidney | 0.21271 | 12 | 0.01429 | Not significant |
| Gastroschisis | 0.27951 | 13 | 0.01548 | Not significant |
| Hydronephrosis | 0.28122 | 14 | 0.01667 | Not significant |
| Double outlet right ventricle | 0.29153 | 15 | 0.01786 | Not significant |
| Congenital pulmonary valve | 0.29600 | 16 | 0.01905 | Not significant |
| Ano-rectal atresia | 0.30304 | 17 | 0.02024 | Not significant |
| Diaphragmatic hernia | 0.30531 | 18 | 0.02143 | Not significant |
| Agenesis of the corpus callosum | 0.34069 | 19 | 0.02262 | Not significant |
| Coarctation of aorta | 0.34767 | 20 | 0.02381 | Not significant |
| Laterality anomalies | 0.35024 | 21 | 0.02500 | Not significant |
| Pulmonary valve atresia | 0.35635 | 22 | 0.02619 | Not significant |
| Aortic valve atresia/stenosis | 0.38391 | 23 | 0.02738 | Not significant |
| Craniosynostose | 0.38956 | 24 | 0.02857 | Not significant |
| Hypoplastic left heart | 0.42034 | 25 | 0.02976 | Not significant |
| Posterior urethral valve | 0.48211 | 26 | 0.03095 | Not significant |
| PDA as only CHD in term infants | 0.55250 | 27 | 0.03214 | Not significant |
| Hirschrung's disease | 0.60947 | 28 | 0.03333 | Not significant |
| Atresia or stenosis of intestine | 0.62903 | 29 | 0.03452 | Not significant |
| Unilateral renal agenesis | 0.63995 | 30 | 0.03571 | Not significant |
| Polydactyly | 0.65687 | 31 | 0.03690 | Not significant |
| Tetralogy of Fallot | 0.81849 | 32 | 0.03810 | Not significant |
| Oesophageal atresia | 0.84340 | 33 | 0.03929 | Not significant |
| Vascular disruption anomalies | 0.84436 | 34 | 0.04048 | Not significant |
| Club foot | 0.84732 | 35 | 0.04167 | Not significant |
| Hypospadias | 0.86456 | 36 | 0.04286 | Not significant |
| Ventricular septal defect | 0.89212 | 37 | 0.04405 | Not significant |
| Omphalocele | 0.89467 | 38 | 0.04524 | Not significant |
| Severe microcephaly | 0.91248 | 39 | 0.04643 | Not significant |
| Anomalies of intestinal fixation | 0.93444 | 40 | 0.04762 | Not significant |
| Renal dysplasia | 0.96615 | 41 | 0.04881 | Not significant |
| Congenital cataract | 0.99638 | 42 | 0.05000 | Not significant |
