# Supplementary figures and images for: First-trimester exposure to macrolides and risk of major congenital malformations compared with amoxicillin: A French nationwide cohort study
Source: PLoS Med. 2025 Apr 15;22(4):e1004576. doi: 10.1371/journal.pmed.1004576 (PMC12021278; doi:10.1371/journal.pmed.1004576)

**S1 Fig.** Flowchart of the study cohort


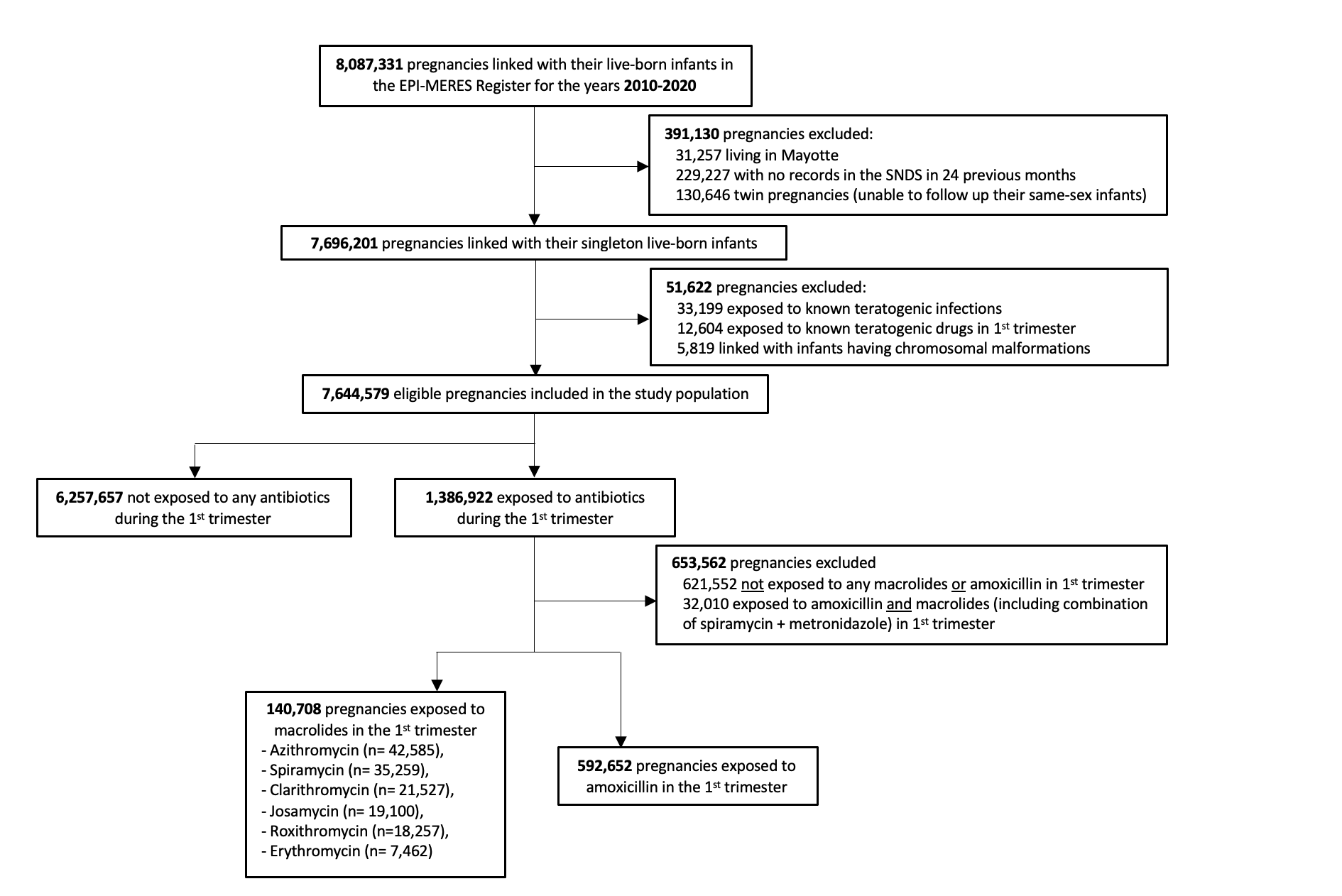

Supplement: S1 Fig — (DOCX) [file pmed.1004576.s018.docx]
