# Supplementary material for: First-trimester exposure to macrolides and risk of major congenital malformations compared with amoxicillin: A French nationwide cohort study
Source: PLoS Med. 2025 Apr 15;22(4):e1004576. doi: 10.1371/journal.pmed.1004576 (PMC12021278; doi:10.1371/journal.pmed.1004576)

**S2 Fig**. Flowchart for comparing the frequency of non-live births among pregnancies exposed to macrolides during the first trimester with those exposed to amoxicillin in the EPI-MERES Register

As the study population was limited to live births, we assessed the potential impact of selection bias in our study. Pregnancies ending in live births and non-live births (spontaneous abortions. therapeutic abortions after 22 gestational weeks, stillbirths, ectopic pregnancies. and other abnormal products of conception) from the EPI-MERES register for the years 2010-2020 were used. Pregnancies ending in early therapeutic (before 22 gestation weeks) and elective abortions were removed from the analysis due to data quality issues. We then compared the frequency of non-live births among pregnancies exposed to macrolides with those exposed to amoxicillin during the first trimester. If the non-live birth outcomes are not strongly associated with the macrolide exposure, any observed association was likely to be unbiased. As presented in the flowchart, the frequency of non-live births among pregnancies exposed to macrolide and those exposed to amoxicillin was 9.1% and 7.3%. respectively. The log-binomial regression with generalized estimating equations was used to estimate the crude relative risk of non-live birth outcomes among pregnancies exposed to macrolides compared with amoxicillin.


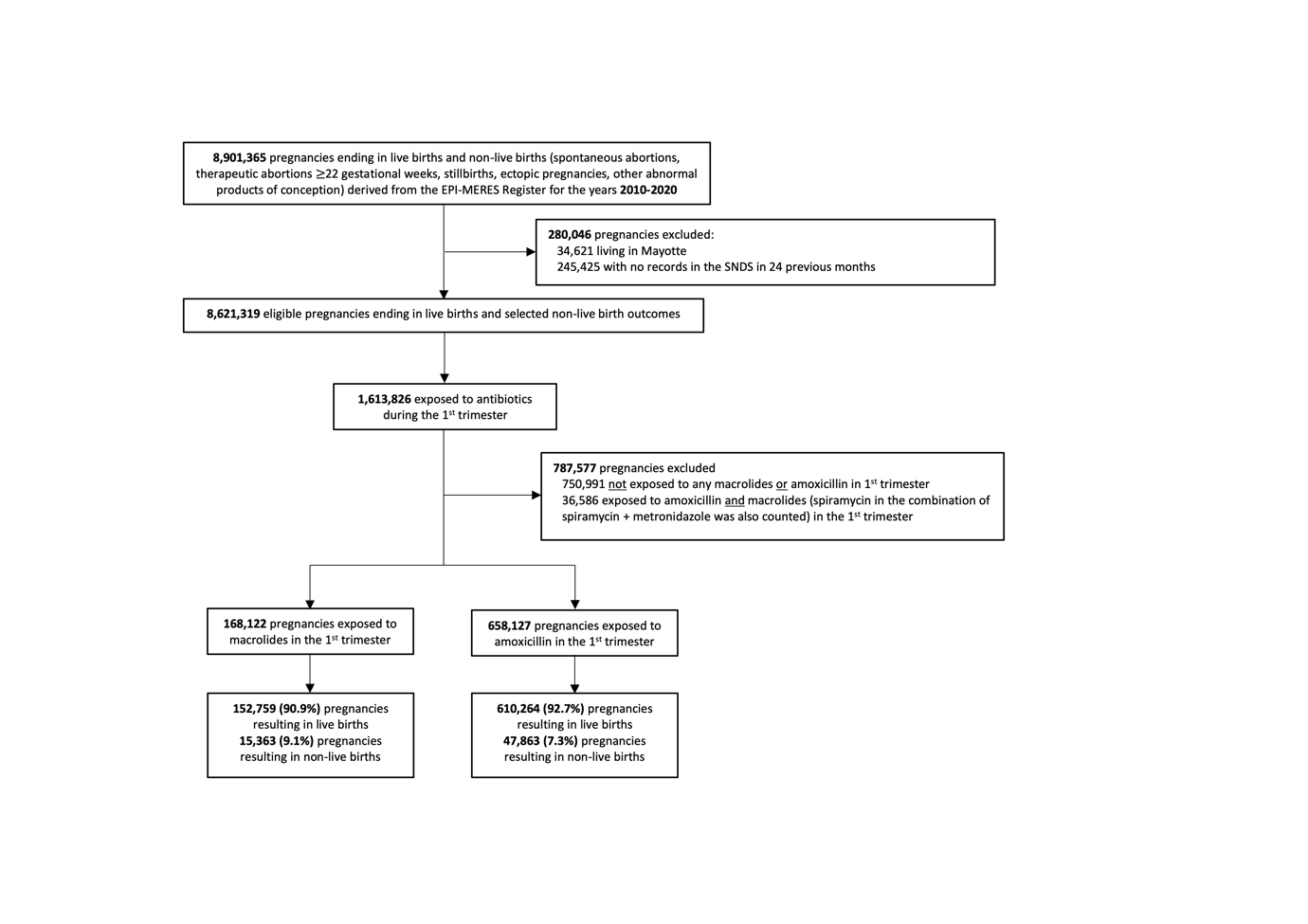

Supplement: S2 Fig — (DOCX) [file pmed.1004576.s019.docx]
