# Supplementary material for: First-trimester exposure to macrolides and risk of major congenital malformations compared with amoxicillin: A French nationwide cohort study
Source: PLoS Med. 2025 Apr 15;22(4):e1004576. doi: 10.1371/journal.pmed.1004576 (PMC12021278; doi:10.1371/journal.pmed.1004576)
Supplement: S1 Protocol — (DOCX) [file pmed.1004576.s021.docx]

**S1 Protocol**. Summary protocol

The study protocol was pre-specified and approved by the scientific committee of the EPI-PHARE team before conducting this study. While the protocol was not registered in a publicly accessible registry, it was documented in the EPI-PHARE repository. Below, we provide a summary of the study protocol.

| **Study Objective** | Evaluate the risk of major congenital malformations (MCMs) associated with exposure to macrolide antibiotics during the first trimester of pregnancy, focusing on several individual MCMs |
| --- | --- |
| **Study Type** | Observational study |
| **Study Design** | Population-based cohort study using data from the French National Health System (SNDS) |
| **Study Population** | Pregnancies linked with their singleton live-born infants derived from the 2010-2020 National Mother-Infant Register nested in the SNDS  - **Inclusion criteria**: the live-born infants from pregnant women aged 15-49 years; those living in French territories (except for Mayotte); those having at least one record in the SNDS for the 2 previous years  - **Exclusion criteria**: those identified with chromosomal abnormalities; infants exposed to teratogenic infections in utero; those exposed to known teratogenic medications during the first trimester |
| **Exposure and comparator groups** | - **Exposure groups:** $\geq$1 prescription filled for at least one macrolide, the first day of last menstrual period (LMP) through 84 days (first trimester of pregnancy)  *Macrolides include erythromycin (J01FA01), spiramycin (J01FA02), roxithromycin (J01FA06), josamycin (J01FA07), clarithromycin (J01FA09), azithromycin (J01FA10)  - **Comparator group**: $\geq$1 prescription filled for amoxicillin (J01CA04), LMP through 84 days |
| **Outcomes** | Risk of MCMs  Infants were followed up to 1 year (2 years for certain MCMs), death, or Dec 31, 2022. |
| **Statistical Analyses** | **Descriptive analysis**  - The number of affected live-born infants and prevalence among 10 000 infants in all exposure groups will be calculated for all individual MCMs.  Main analysis  - Baseline characteristics will be compared for pregnancies exposed to macrolides and those exposed to amoxicillin, using standardized differences.  - Propensity score fine stratification weighting is adopted to control potential confounders. We plan to estimate a separate propensity score for macrolide-exposed group using a logistic regression model that will include all covariates chosen a priori. Observations from non-overlapping regions of the propensity score distribution will be trimmed. We will create 50 strata based on the distribution of pregnancies exposed to macrolides. The pregnancies exposed to amoxicillin are weighted using the distribution of those exposed to macrolides among the propensity score strata and covariate balance will be reassessed.  - Crude and adjusted relative risks (RR) with 95% confidence interval (CI) are estimated using log-binomial regression with a weighting statement for individual MCMs.  **Subgroup analysis**  - Analyses are repeated for the groups exposed to each of the six individual macrolides  **Sensitivity analysis***:*   1. We redefine the exposure time window as between 5^th^ and 12^th^ week after the LMP 2. We restrict the study population to pregnancies having prescriptions filled for only one antibiotic agent during the first trimester 3. We conduct the negative control analysis by using the exposure time window as between 20^th^ and 32^nd^ week after the LMP   **Supplementary analysis**:   1. We compare the group exposed to macrolides with the group of pregnancies not exposed to any antibiotics from one month before pregnancy through the end of the first trimester 2. We examine the risk of overall MCMs and organ-specific groups 3. We stratify analyses for neural tube defects by the folic acid supplementation |
