# Supplementary material for: First-trimester exposure to macrolides and risk of major congenital malformations compared with amoxicillin: A French nationwide cohort study
Source: PLoS Med. 2025 Apr 15;22(4):e1004576. doi: 10.1371/journal.pmed.1004576 (PMC12021278; doi:10.1371/journal.pmed.1004576)
Supplement: Attachment — Submitted filename: Response to reviewers.docx [file pmed.1004576.s025.docx]

**COMMENTS from the Plos Medicine’s academic editor:**

**COMMENT 1**: Given that macrolides were tested against many different outcomes, it seems fairly likely that a statistically significant result could occur due to random chance. In this circumstance, it seems essential that results that are adjusted for multiple comparisons also be presented (even if original estimates are also maintained).

**RESPONSE**: We thank the editor for this comment. One of the strengths of our study lies in its focus on individual major congenital malformations (MCMs), rather than combining them into broader, organ-specific groups, which would have reduced the number of comparisons. However, aggregating such heterogeneous sets can mask potential effects. We acknowledge that analyzing multiple individual MCMs raises concerns about the possibility of false-positive findings due to multiple comparisons. Our primary objective was to avoid overlooking potential safety signals that warrant further investigation rather than prematurely dismissing associations solely based on statistical significance thresholds. To achieve this, we emphasized interpreting our findings based on the strength of associations, the precision of confidence intervals, the consistency observed across sensitivity analyses, and the null findings obtained negative control analysis rather than relying on statistical significance. This interpretation approach aligns with the methodology adopted in several previous studies [1,2].

However, to provide further transparency for readers, we performed a correction for multiple testing using the Benjamini-Hochberg method in the main analysis. Given the number of analyses conducted and the limited number of events in the exposed cohorts, it was unsurprising that none of the associations yielded a p-value below the corrected significance threshold. We mentioned this information in the Discussion section while still emphasizing in the manuscript that our interpretations were driven by the overall evidence rather than the statistical significance.

“To avoid overlooking potential safety signals that may warrant further investigation, no adjustments were made for multiple testing. Instead, we opted to interpret the results based on the association strength, the confidence interval width, the consistency in the results of sensitivity analyses, and the null findings in the negative control analysis. This practice has been adopted in previous studies [22,23].” (Methods section, page 11, lines 217-221)

“In our study, 5% of associations analyzed were expected to be statistically significant simply by chance. To provide transparency for readers, we applied the Benjamini-Hochberg correction with a 5% false discovery rate [36], and it was unsurprising that none of the associations in the main analysis remained statistically significant given the number of analyses conducted and the limited number of macrolide-exposed events. Although we acknowledge the possibility of false-positive findings, the p-value use could lead to missing potential safety signals [37]. Therefore, our interpretation was based on the association strength, the confidence interval width, the consistency of the results in sensitivity analyses, and the null negative control association.” (Discussion section, page 16, lines 338-346)

Additional references added:

Huybrechts KF, Straub L, Karlsson P, et al. Association of In Utero Antipsychotic Medication Exposure With Risk of Congenital Malformations in Nordic Countries and the US. JAMA Psychiatry. 2023;80: 156. doi:10.1001/jamapsychiatry.2022.4109

Patorno E, Hernandez-Diaz S, Huybrechts KF, et al. Gabapentin in pregnancy and the risk of adverse neonatal and maternal outcomes: A population-based cohort study nested in the US Medicaid Analytic eXtract dataset. Stock SJ, editor. PLoS Med. 2020;17: e1003322. doi:10.1371/journal.pmed.1003322

**COMMENT 2**: Another way to evaluate the robustness of the results would be to assess with association between macrolides and spinal bifida/syndactyly hold up in stratified analyses. One would expect the effect size would be similar across characteristics strata presented in Table 1 if these associations are indeed real. This could be presented as a supplement.

**RESPONSE:** We thank the editor for this valuable suggestion. We have conducted post-hoc analyses to assess whether the associations between macrolides and spina bifida/syndactyly remained consistent across different strata of characteristics. The results are presented in the Supplementary File. While we agree that stratified analyses could provide additional insights, major congenital malformations (MCMs) are rare outcomes, and the number of exposed cases within each stratum of the characteristics in Table 1 is limited. This lack of statistical power made it challenging to generate reliable estimates and draw meaningful conclusions from such analyses in some strata. Additionally, certain characteristics, such as lifestyle factors, were not adequately captured in the SNDS, further complicating the interpretation of stratified results. Nevertheless, we believe that these post-hoc analyses contributed to understanding the observed associations.

“For post-hoc analysis, stratified analyses were conducted based on certain baseline characteristics to determine whether the associations observed in the main analysis remained consistent across different subgroups.” (Methods section, page 11, lines 207-209)

“For post-hoc analysis, the aRRs for spina bifida and syndactyly remained elevated across subgroups (Table S14). In certain strata, the aRRs were towards the null with the limited number of exposed events” (Results section, page 14, lines 283-285)

“Given the limitations in accurately capturing certain variables in the SNDS, the results from subgroup analysis among pregnancies with reimbursed folic acid and from the stratified post-hoc analyses should be interpreted cautiously.” (Discussion section, page 17, lines 363-365)

**Reviewer 1:**

This study utilizes data from the Mother-Child EPI-MERES Register nested within the French Health Data System (SNDS) to conduct a large population-based cohort study. The findings indicate that the use of macrolide antibiotics by mothers during the first trimester does not have a strong teratogenic effect, but may increase the risk of spina bifida and syndactyly in offspring. This study has an adequate sample size, a reasonable research design, rigorous statistical analysis, fluent text expression, and interesting results that are of guiding significance for clinical maternal and child health care.

**RESPONSE:** We thank the reviewer for the comment.

I suggest that this study be published after addressing the following issues:

**COMMENT 1:** This study focuses on the impact of macrolide antibiotic use in the first trimester (from the first day of LMP to the end of the 12th gestational week) on major congenital malformations in offspring. However, considering that antibiotic exposure during the periconceptional period (shortly before pregnancy) may have residues in the body, how can the impact of this situation be excluded?

**RESPONSE**: We thank the reviewer for this comment. Major congenital malformations typically arise during the organogenesis period, which spans from week 3 to the end of week 8 after conception, corresponding to gestational weeks 5 to 10 after the last menstrual period (LMP) [3]. Since our exposure assessment began on the first day of LMP, it inherently captured antibiotic exposure occurring up to four weeks before the start of the organogenesis period. As antibiotics are usually short-term treatments, this approach ensures that potential residual effects from antibiotics during the periconceptional period are considered in the analysis. To clarify this, we have modified the sentence in the Methods section (page 8, lines 137-140): “The exposure to macrolides was defined as filling one or more prescriptions for any macrolide in outpatient settings during the first trimester, spanning from the first day of the last menstrual period (LMP) to the end of the 12th gestational week. This period encompasses the critical organogenesis phase, which occurs between the 5th and 10th gestational weeks, when the risk of MCMs is highest [18].”

Additional references included:

Moore KL, Persaud TV, Torchia MG. The Developing Human: Clinically Oriented Embryology. Ninth edition.

**COMMENT 2**: The study defines antibiotic exposure as oral macrolide use. How should the impact of other exposure routes, such as dermal intake, be considered?

**RESPONSE**: First, we would like to thank the reviewer for this comment, as we were not clear enough about the route of administration. In this study, antibiotic exposure was defined as the use of systemic macrolides including both oral and injectable routes. Regarding other exposure routes, such as dermal intake, certain macrolides, such as erythromycin, are available for treating skin conditions. For example, there were 1.75% (n=2,465) of pregnancies in the macrolide exposure group and 1.16% (n=6,898) of those in the amoxicillin exposure group having at least one prescription filled for topical erythromycin one month before pregnancy until the end of the first trimester. However, studies have shown that systemic absorption of topical erythromycin is 10-100 times lower than that of oral administration [4]. Therefore, the potential impact of dermal intake on systemic exposure is minimal and unlikely to significantly affect the results of this study. We have changed from “oral macrolides” to “systemic macrolides” in the Methods section (line 134) to reflect more precisely our exposure.

**COMMENT 3:** Given the evaluation of the effects of multiple antibiotics on various subtypes of birth defects, why did the authors not employ multiple testing analysis to correct the statistical results?

**RESPONSE:** The comment aligns with comment #1 from the Academic Editor. As we mentioned in the response to comment #1 from the Academic Editor, one of the strengths of our study lies in its focus on individual MCMs rather than combining them into broader, organ-specific groups, which could have reduced the number of comparisons. However, aggregating such heterogeneous sets can mask potential effects. We acknowledge that analyzing multiple individual MCMs raises concerns about the possibility of false-positive findings due to multiple comparisons. Our primary objective was to avoid overlooking potential safety signals that warrant further investigation rather than prematurely dismissing associations solely based on statistical significance thresholds. To achieve this, we emphasized interpreting our findings based on the strength of associations, the precision of confidence intervals, the consistency observed across sensitivity analyses, and the null findings obtained negative control analysis rather than relying on statistical significance. This interpretation approach aligns with the methodology adopted in several previous studies.

However, to provide further transparency for readers, we performed a correction for multiple testing using the Benjamini-Hochberg method in the main analysis. Given the number of analyses conducted and the limited number of events in the exposed cohorts, it was unsurprising that none of the associations yielded a p-value below the corrected significance threshold. We mentioned this information in the Discussion section while still emphasizing in the manuscript that our interpretations were driven by the overall evidence rather than the statistical significance.

“To avoid overlooking potential safety signals that may warrant further investigation, no adjustments were made for multiple testing. Instead, we opted to interpret the results based on the association strength, the confidence interval width, the consistency in the results of sensitivity analyses, and the null findings in the negative control analysis. This practice has been adopted in previous studies [22,23].” (Methods section, page 11, lines 217-221)

“In our study, 5% of associations analyzed were expected to be statistically significant simply by chance. To provide transparency for readers, we applied the Benjamini-Hochberg correction with a 5% false discovery rate [36], and it was unsurprising that none of the associations in the main analysis remained statistically significant given the number of analyses conducted and the limited number of macrolide-exposed events. Although we acknowledge the possibility of false-positive findings, the p-value use could lead to missing potential safety signals [37]. Therefore, our interpretation was based on the association strength, the confidence interval width, the consistency of the results in sensitivity analyses, and the null negative control association.” (Discussion section, page 16, lines 338-346)

Additional references added:

Huybrechts KF, Straub L, Karlsson P, et al. Association of In Utero Antipsychotic Medication Exposure With Risk of Congenital Malformations in Nordic Countries and the US. JAMA Psychiatry. 2023;80: 156. doi:10.1001/jamapsychiatry.2022.4109

Patorno E, Hernandez-Diaz S, Huybrechts KF, et al. Gabapentin in pregnancy and the risk of adverse neonatal and maternal outcomes: A population-based cohort study nested in the US Medicaid Analytic eXtract dataset. Stock SJ, editor. PLoS Med. 2020;17: e1003322. doi:10.1371/journal.pmed.1003322

**COMMENT 4**: It is recommended to add a discussion on the possible biological mechanisms of macrolide antibiotics leading to spina bifida or syndactyly in the discussion section.

RESPONSE: We thank the reviewer for this comment. Macrolides are known to cross the placenta and reach the fetus, raising concerns about the risk of MCMs. One proposed hypothesis is that macrolides may induce apoptosis [5], a process of programmed cell death that plays a critical role in shaping tissues and organs during organogenesis. If macrolides induce apoptosis in certain fetal tissues during the organogenesis period, it could disrupt normal development and lead to MCMs, including spina bifida or syndactyly. However, it is important to note that this remains a hypothetical mechanism, and to our knowledge, no established biological pathway currently explains the observed associations between first-trimester macrolide exposure and these specific malformations. Given the lack of definitive biological evidence, our findings should be interpreted with caution.

We have added a discussion in the manuscript: “There is some evidence from animal studies showing that prenatal exposure to certain macrolide antibiotics can cause fetal developmental toxicity [34]. To date, no established biological mechanism explains the observed associations with spina bifida and syndactyly. One proposed hypothesis is that macrolides can induce apoptosis [35], an important process in shaping tissues and organs during organogenesis. If macrolides induce apoptosis in certain fetal issues during the organogenesis period, it could disrupt normal fetal development and lead to MCMs.” (Discussion section, page 15, lines 318-324)

Additional references included:

Ishimatsu Y, Kadota J-I, Iwashita T, et al. Macrolide antibiotics induce apoptosis of human peripheral lymphocytes in vitro. International Journal of Antimicrobial Agents. 2004;24: 247–253. doi:10.1016/j.ijantimicag.2004.03.022

**Reviewer 2:**

The authors present a large body of work exploring the association between macrolide exposure in early pregnancy and major congenital malformations, compared with amoxicillin exposure. The study has been well designed and conducted with several sensitivity analyses to explore the robustness of the findings.

**RESPONSE**: We thank the reviewer for the comment.

Regarding the statistical approach, the authors have utilised a propensity score based fine stratification method and used 50 strata. These methods are appropriate, and they show balance achieved for the included covariates. Specific comments:

**COMMENT 1**: Did the authors consider alternate models to achieve further balance rather than trimming?

**RESPONSE**: In response, we have used propensity score overlap weighting as an alternative method to achieve balance without trimming. The results remained similar for the main analysis when applying overlap weighting. We have now included this analysis as a post-hoc analysis and presented the results in the Supporting Information(S15 Table).

“Additionally, we applied propensity score overlap weighting as an alternative method for fine stratification propensity score in the main analysis to evaluate the sensitivity of results to weighting methods.” (Methods section, page 11, lines 210-212)

COMMENT 2: Additionally, how were covariates selected for the models? Were DAGs considered for confounders? Given both control and exposed groups are antibiotic exposed they are likely similar and few variables would be considered confounders.

**RESPONSE**: Directed Acyclic Graphs (DAGs) were not used to identify confounders in our study. Instead, covariates were selected based on a comprehensive literature review and expert knowledge. The covariates included in the propensity score models were chosen as potential confounders or factors known to be associated with major congenital malformations (MCMs) or their proxies.

**COMMENT 3**: Missing data - what proportion of data were missing and how was this handled?

**RESPONSE**: As our study used claims data, missing values were minimal among the covariates included in our study. For the deprivation index, missing data were categorized as a separate group, ensuring they were accounted for in the analysis. No missing data were observed for the other covariates. This information has been clarified in the Methods section of our manuscript (page 9, lines 167-169) “For the deprivation index, missing data were treated as a separate category, while no missing data were observed for other covariates.”

Other considerations:

**COMMENT 4**: Title: consider adding mention to the comparison to amoxicillin - this better reflects the actual study.

**RESPONSE**: We thank the reviewer for the suggestion. We have changed the title accordingly: “First-trimester exposure to macrolides and risk of major congenital malformations compared with amoxicillin: A French nationwide cohort study”.

**COMMENT 5**: Abstract: include the overall RR in the abstract and 95% CI. Line 46 of the conclusion should be amended to association and not effect - this is a population based study. Consider 'are not strongly associated with'.

**RESPONSE**: We thank the reviewer for the suggestion. The overall RR for any MCM has been added, and the Conclusions in the Abstract have been revised accordingly.

“After adjustment for measured confounders, macrolide exposure during the first trimester was not associated with any MCM overall (aRR 1.00, 95% CI 0.96-1.05) compared with amoxicillin.” (Abstract, page 2, lines 37-39)

“The findings indicate that macrolide exposure during the first trimester is not strongly associated with an increased risk for most individual MCMs, which is reassuring.” (Abstract, page 3, lines 48-49)

**COMMENT 6**: Introduction: objective should be updated to include amoxicillin as - ie exposure to macrolides compared with amoxicillin to better reflect the study.

**RESPONSE**: We have modified the objective in the introduction: “Using data from the French National Health System (SNDS), one of the largest databases of its kind, this study aimed to evaluate the association between first-trimester exposure to macrolides and the risk of MCMs, with a specific focus on individual MCMs, compared with amoxicillin.” (Page 6, lines 93-96)

Results:

**COMMENT 7**: Why is maternal age highlighted by exposure groups but not other covariates? Further discussion on how the two groups differ is needed.

**RESPONSE**: We have modified and expanded the results section to highlight how the two exposure groups differed before propensity score adjustment. “Pregnancies exposed to macrolides were more likely to be among women having assisted reproduction and have lower mean gestational age at the time of first exposure to the antibiotic of interest compared to pregnancies exposed to amoxicillin (Table 1 and S6 Table). The two groups were otherwise similar in terms of other baseline characteristics.” (Page 12, line 233-236)

**COMMENT 8:** Include the percent of each macrolide rather than only n.

**RESPONSE**: We have now included the percentage for each macrolide in the Results section, page 12, lines 230-232.

“Among the macrolide exposure group, pregnancies were exposed most frequently to azithromycin (n=42,585; 30.3%), followed by spiramycin (n=35,259; 25.1%), clarithromycin (n=21,527; 15.3%), josamycin (n=19,100; 13.6%), roxithromycin (n=18,257; 13.0%), and erythromycin (n=7,462; 5.3%).”

**COMMENT 9**: Line 200 - is this trying to get to suggest nulliparity? May be better to say that it was the proportion of women with only one live birth in the dataset or nulliparous based on data from 2010 - 2020.

**RESPONSE**: In our study, "parity" was defined as the number of live births recorded since 2010. As suggested in comment #20 by reviewer 3, due to left censoring in the data, this variable does not accurately reflect true nulliparity and is, therefore, not meaningful. As a result, we have removed this variable from the propensity score model and revised the manuscript accordingly. This adjustment had minimal impact on the results, and the interpretation of the findings remained unchanged.

**COMMENT 10**: Needs to clarified whether prevalence per 10,000 is based on raw data or adjusted throughout.

**RESPONSE**: We have clarified in the manuscript that the prevalence per 10,000 is based on raw data and is referred to as the "crude prevalence" throughout (Methods section, page 9, line 169).

**COMMENT 11**: Suggest including the unadjusted relative risk for the overall results and adjusted - this gives the reader an idea of the impact of confounding

**RESPONSE**: We thank the reviewer for this suggestion. We have added the unadjusted relative risks and the overall major congenital malformation risk at the top of Figure 1.

**COMMENT 12**: Line 221 consider comparing the prevalence statistically and whether this is a clinically meaningful difference

**RESPONSE:** We have estimated adjusted risk differences (RD), which more accurately quantify the difference in the risk of MCMs between the macrolide and amoxicillin exposure groups. Additionally, we have expanded the Discussion section to address the clinical relevance of these findings.

“The adjusted RD per 10,000 live-born infants was 1.15 (95% CI 0.26-2.05) for spina bifida and 0.87 (95% CI 0.01-1.72) for syndactyly (S7 Table).” (Results section, page 13, lines 254-256)

“Conversely, increased risks of spina bifida and syndactyly following exposure to macrolides were observed. While associations were observed on a relative scale, the risk differences were relatively small.” (Discussion section, page 15, lines 305-307)

**COMMENT 13**: line 226: include a summary of these supplemental findings, they show the robustness of the findings.

**RESPONSE**: We have added a summary of the results from sensitivity analyses for spina bifida and syndactyly in the Results section.

“The elevated aRRs for spina bifida and syndactyly persisted across sensitivity analyses, with spina bifida showing a significant increase in the monotherapy analysis (aRR 2.02, 95% CI 1.31–3.01) and syndactyly in the narrower exposure window analysis (aRR 2.38, 95% CI 1.42–4.01).” (Results section, page 13, lines 256-262)

**COMMENT 14**: Replace 'RR estimates'; could be along the lines of the association between /the adjusted relative risk etc

**RESPONSE**: We have replaced ‘RR estimates’ with adjusted relative risks throughout the manuscript.

**COMMENT 15**: Line 235 - needs more detail, a broad statement for a result - did the 95% CIs cross 1? Difficult to draw conclusions from this.

**RESPONSE**: We have now revised the paragraph in the Results section with more details:

“Estimates for spina bifida and syndactyly were consistently elevated in most subgroup analyses, except for certain associations (spiramcyin/erythromycin with spina bifida, erythromycin with syndactyly). The aRRs of spina bifida ranged from 1.57 (95% CI 0.80-3.08) for azithromycin to 2.73 (95% CI 1.25-6.00) for roxithromycin. For syndactyly, aRRs ranged from 1.55 (95% CI 0.60-4.01) for clarithromycin to 2.22 (95% CI 0.90-5.51) for josamycin.” (page 13, lines 268-273)

**COMMENT 16:** A major consideration is that folic acid is often available over the counter, thus if this is obtained via linkage there will be many women in the control group who are indeed taking folic acid - introducing significant bias into this analysis. Is the point of this analysis to show that folic acid may attenuate this association? If so, the lack of accurate exposure is a major limitation and the authors should consider removing this analysis.

**RESPONSE**: We thank the reviewer for this comment. Since folic acid supplementation is often available over the counter, its use cannot be completely captured in the reimbursement data. We then revised the term in the manuscript to “reimbursed folic acid supplementation” to reflect the variable more accurately. However, we have retained the stratified analysis to give additional insights, as suggested by the Academic Editor. In this analysis, among pregnancies having reimbursed folic acid supplementation (i.e., pregnancies for whom it is certain that folic acid was dispensed), the association with spina bifida was attenuated towards the null. In this subgroup, it is possible that some pregnant women had taken over-the-counter folic acid; however, there is no argument to think that the use of over-the-counter folic acid was different between the two exposure groups (macrolides and amoxicillin). This could lead to a non-differential misclassification bias, resulting in a loss of statistical power and a bias toward the null hypothesis. Although this is a limitation in our study, we thought it would be interesting to present the results of this analysis. In fact, if acid folic supplementation during the first trimester of pregnancy among women exposed to macrolides no longer shows an association with spina bifida, it is important to present the results of this analysis, as it may help reinforce the need to receive folic acid during the first trimester.

“Given the limitations in accurately capturing certain variables in the SNDS, the results from subgroup analysis among pregnancies with reimbursed folic acid and from the stratified post-hoc analyses should be interpreted cautiously.” (Discussion section, page 17, lines 363-365)

COMMENT 17: Live birth bias analysis is discussed in discussion but not results - should be added if discussed and were any statistical analysis done on this to compare the difference?

**RESPONSE**: We have now added the statistical analysis that was used for live birth bias assessment and its results in the manuscript.

“Finally, since the study population was limited to live births, we assessed the crude RR of non-live births among pregnancies exposed to macrolides during the first trimester compared with those exposed to amoxicillin in the EPI-MERES register. If macrolide exposure is not strongly associated with non-live births, any observed association for high mortality MCMs is unlikely to be biased [21].” (Methods section, page 11, lines 212-216)

“Finally, the crude RR for non-live births among pregnancies exposed to macrolides during the first trimester compared to amoxicillin in the EPI-MERES Register was 1.26 (95% CI 1.23-1.28) (S2 Figure).” (Results section, page 14, lines 286-288)

“First, the study population was restricted to pregnancies resulting in live births, as information on MCMs was rarely available for pregnancies resulting in non-live birth outcomes. Since macrolide exposure was shown not to be strongly associated with non-live births, this bias is unlikely to affect our results.” (Discussion section, page 17, lines 350-354)

**COMMENT 18**: BMI: Is there no information on BMI? The obesity related conditions will only capture women hospitalised but not overall BMI, this is an important maternal characteristic in the setting of anomalies. If this data is not available it should be acknowledged.

**RESPONSE**: We thank the reviewer for this comment. As the reviewer noticed, BMI data is not available in the SNDS. To account for maternal obesity, we utilized obesity-related diagnoses recorded in hospital discharge data, including primary, related, or associated diagnoses for any hospitalization occurring from five years before pregnancy up to the pregnancy outcome, including delivery hospitalization. Maternal obesity is a recognized risk factor for MCMs and is usually documented during hospitalizations in pregnancy. In our study, obesity-related diagnoses were recorded for 6.4% of pregnancies in the amoxicillin exposure group, which is in line with the obesity rate of 9.1% reported in France between 2009 and 2011 [6], considering potential underreporting in medico-administrative data. While this approach allowed us to partially account for obesity, we still acknowledge the limitation that we could not assess obesity severity due to the absence of maternal BMI measurements.

“Fourth, there was potential residual confounding by unmeasured and poorly measured variables in the SNDS, such as maternal body mass index and lifestyle factors, which may be risk factors for birth defects [38,39].” (Discussion section, page 17, lines 359-361)

Additional references added:

Persson M, Cnattingius S, Villamor E, Söderling J, Pasternak B, Stephansson O, et al. Risk of major congenital malformations in relation to maternal overweight and obesity severity: cohort study of 1.2 million singletons. BMJ. 2017;357: j2563. doi:10.1136/bmj.j2563

**COMMENT 19:** Tables: Pregnancy related conditions - parity and folic acid are not conditions - adjust titles

**RESPONSE:** We now have removed the “parity” variable to respond to comment #20 from the Reviewer 3 and changed the category name to “Pregnancy-related healthcare utilization”

**COMMENT 20**: Table 2 - are these unadjusted prevalences? If so this needs to be made clear.

**RESPONSE**: We have modified the table title with “crude prevalence”.

**COMMENT 21**: Table S10 - describes compared with the unexposed group but the table still shows amoxicillin - not total unexposed.

**RESPONSE**: We now have corrected the table.

**COMMENT 22**: Figure 1: consider including the unadjusted relative risk to clearly show the impact of confounding/adjustment on the results. Additionally, including the overall major congenital malformation risk at the top of the table is useful.

**RESPONSE:** We have added the unadjusted relative risks and the overall major congenital malformation risk at the top of Figure 1.

COMMENT 23: Figure 2 is difficult to interpret. Why not include the 95% CIs so the reader can draw their conclusions off this data. The figure is large, considered removing or condensing.

**RESPONSE**: We thank the reviewer for the suggestion. In response, we have moved Figure 2 to the Supporting Information in the table format (S9 Table). Additionally, we have included the 95% confidence intervals for each result to enhance interpretability and allow readers to draw more informed conclusions.

**Reviewer 3:**

Thank you for the opportunity to review this manuscript. The authors present an interesting study to understand the association between macrolide antibiotics, which were commonly used in pregnancy, and major congenital malformations using the French National Health System database. Overall, this study is well-designed, but I would like to suggest a few clarifications and suggestions to further strengthen the manuscript.

**RESPONSE**: We thank the reviewer for this comment.

Introduction

**COMMENT 1**: Line 66: Please mention the types of studies included in the meta-analysis (eg, case-control, cohort design) and their pooled sample sizes in the main text.

**RESPONSE**: The following sentence has now been revised in the Introduction section (page 5, lines 86-88): “The latest meta-analysis by Keskin-Arslan et al., which included 18 cohort and case-control studies involving 21,541 infants exposed to overall macrolides, did not suggest an increased risk of MCMs following all macrolide exposure except for a small increase in risk following azithromycin exposure.”

RESPONSE:

Method - Data sources

**COMMENT 2**: Line 81: Can you provide a bit further details on the database used? For example, please provide an essential explanation of the SNDS (eg, the type of data source) or provide relevant references. Regarding the EPI-MERES register, please provide simple linkage methods and linkage % from the total population. As for the date of conception, can you briefly describe how this information is collected in this database?

**RESPONSE**: We have provided the details on the SNDS in the Supporting Information (S1 Text). We also have added information on the type of data sources, the mother-infant linkage, and the date of conception in the Methods section.

“The SNDS contains comprehensive health insurance reimbursement and hospitalization data for over 99% of the French population.” (Methods section, page 6, lines 100-101)

“The mothers were linked with their infants primarily using a deterministic algorithm, with a linkage rate exceeding 95%. The date of conception was calculated by subtracting the gestational age, which is mostly available in the hospitalization data, from the pregnancy outcome date.” (Methods section, page 6, lines 105-108)

**COMMENT 3**: Line 90: Could you elaborate on "Data for same-sex twins, however, are not included due to technical constraints."? Does that mean that you cannot distinguish between the twins in the data? Or we cannot see the record of the outcome at all?

**RESPONSE**: The data in the SNDS does not allow to distinguish between same-sex twins. As a result, we were unable to identify outcomes for each twin separately in such cases. We have modified the relevant sentence: “However, data for same-sex twins are excluded as we cannot distinguish between the twins in the dataset.” (Methods section, page 6, lines 108-109)

Method - Study population

**COMMENT 4**: Line 104: Is there any reason why the patients were restricted to those who had at least one record in the SNDS in the 2 preceding years?

**RESPONSE**: We excluded pregnant women without healthcare records in the SNDS during the 2 years preceding pregnancy (representing less than 1% of the population) as these women were likely to reside outside France before or during the pregnancy period. This criterion was necessary for the assessment of exposures and covariates, as these rely on reimbursement data in the SNDS. We have clarified this in the Methods section: “We identified pregnancies from women aged 15-49 years living in French territories (excluding Mayotte) who had at least one record in the SNDS in the two preceding years (to ensure reliable exposure and covariate assessments) and linked with their singleton live-born infants in the EPI-MERES for the years 2010-2020.” (Page 7, line 124)

**COMMENT 5**: Line 106: Could you clarify the definition or codes for chromosomal abnormality?

**RESPONSE:** The codes used to identify chromosomal abnormalities, along with other major congenital malformations, are detailed in S3 Table of the Supporting Information. We have added the following clarification to the manuscript: “Details of the algorithms, including those for chromosomal anomalies, are provided in S3 Table.” (Page 8, lines 154-155).

**COMMENT 6**: Line 109-111: Is there any reference about "Pregnancies with three or more prescriptions filled for spiramycin between the date of conception and the date of delivery were classified as having Toxoplasmosis and excluded at this stage."?

**RESPONSE**: We thank the reviewer for this comment. In France, spiramycin is prescribed to pregnant women as soon as a maternal toxoplasmosis infection is diagnosed, and the treatment is continued until delivery. Since toxoplasmosis is a teratogenic infection, we aimed to exclude pregnancies that may have been affected by this infection. Based on the above-mentioned practice, we assumed that pregnant women who had at least three prescriptions for spiramycin during pregnancy were likely to have maternal toxoplasmosis and thus excluded them from the study. As spiramycin is a part of our work, we have clarified this assumption and modified the sentence accordingly in the Methods section.

“Pregnancies with three or more prescriptions filled for spiramycin between conception and delivery were assumed to have a maternal toxoplasmosis infection in our algorithms and excluded.” (Methods section, page 7, lines 130-132)

Method - Exposure

**COMMENT 7**: Line 116: Is there any reason or rationale for including only oral formulations while excluding injectables?

**RESPONSE**: We thank the reviewer for this comment. We were not clear enough about this. We did not exclude injectable formulation of macrolides from our study. To clarify, the SNDS database captures antibiotic use in outpatient settings but does not capture antibiotic use during hospitalizations. We have revised the relevant sentence (Methods section, lines 136-138) in the manuscript to avoid any ambiguity: “The exposure to macrolides was defined as having one or more prescriptions filled for any systemic macrolide in outpatient settings during the first trimester, spanning from the first day of LMP to the end of the 12th gestational week.”

**COMMENT 8**: Line 119: Does the 'index date' mean the start date of follow-up? If not, please avoid using the 'index date' term. If any, please clarify the start and end dates of follow-up as well as censoring criteria in the Method section.

**RESPONSE**: We thank the reviewer for the comment. The index date mentioned referred to the time point to define exposure in the study (the first day of exposure). As suggested by the reviewer, we have removed the term “index date” from the manuscript.

Method - Outcomes

**COMMENT 9**: Potential outcome misclassification: Is there any validation study or positive predictive values (PPVs) for the outcome (MCMs)?

**RESPONSE**: We thank the reviewer for this question. No validation studies or positive predictive values for the major congenital malformations (MCMs) in the SNDS have been conducted. However, the MCMs selected from the EUROCAT classification are accurately identifiable in the hospital discharge database of the SNDS. As enrollment in the French health insurance system is mandatory for all residents, the completeness of follow-up data for infants was ensured for outcome ascertainment in our study. Additionally, the ICD-10 diagnosis codes and medical procedures used in our algorithms have been applied in previous research, notably the study by Blotière et al. (2019). The study of Blotière et al., which investigated the association between prenatal exposure to antiepileptic drugs and the risk of MCMs, successfully confirmed the teratogenicity of valproic acid and topiramate, further supporting the reliability of our algorithms.

We also compared the prevalence of MCMs among live births detected by our algorithms with the prevalence of MCMs reported by the EUROCAT [7]. From 2010 to 2021, the prevalence of MCMs was 181 cases per 10,000 births in the EPI-MERES Register (compared to 204 in the EUROCAT database). This prevalence increased from 168 cases in 2010-2012 to 189 in 2019-2021 (versus an increase from 202 to 204 in the EUROCAT. The prevalence of individual MCM subtypes among live births was generally consistent with EUROCAT data (median relative difference: -3%), though it was markedly lower among stillbirths and fetuses from medical terminations (-96%). This comparison has been added in the Supporting Information (S3 Table).

“The algorithms have been previously applied in the study by Blotière et al. (2019) [24], which successfully confirmed the association between valproate and the risk of MCMs.” (Methods section, page 8, lines 155-157)

Additional references added:

Blotière P-O, Raguideau F, Weill A, Elefant E, Perthus I, Goulet V, et al. Risks of 23 specific malformations associated with prenatal exposure to 10 antiepileptic drugs. Neurology. 2019;93. doi:10.1212/WNL.0000000000007696

**COMMENT 10**: Did you define a minimum follow-up period, such as at least one or two years of follow-up to assess the outcome (except for censoring criteria)? For example, the inclusion period would be 2010-2020 and the follow-up period would be up to 2022. Or were infants with a follow-up time of only one day included as well? Please clarify the end dates of follow-up (or study period) in this study.

**RESPONSE**: Although the mother-infant linkage covers data from 2010 to 2020, follow-up data for both mothers and infants were available until 2022 at the time of analysis. To address this, we have clarified in the Methods section: “The follow-up data were available through 2022.” (Page 8, line 153)

Method - Covariates

**COMMENT 11**: Please clarify the measurement window for some covariates, such as lifestyle factors.

**RESPONSE:** Their assessment period spanned from 5 years prior to pregnancy until the end of pregnancy, including delivery hospitalizations. We have added the assessment window for three lifestyle factors and obesity-related conditions in S4 Table (Supporting Information).

**COMMENT 12:** Hypertension was defined based on antihypertensive drug prescriptions. However, these drugs have many other indications than hypertension (eg, migraine, anxiety, heart failure, etc). How about using the term 'antihypertensive drug use' rather than 'hypertension'? As for obesity, I doubt the accuracy of the E66 code, as obesity is not usually well-recorded in the claims database. I suggest at least using the term "clinical visits due to obesity". As for diabetes, some antidiabetic drugs can have other indications (such as obesity, and PCOS). I suggest using the term "antidiabetic drug use" rather than diabetes.

**RESPONSE**: We thank the reviewer for the suggestion. In response, we have revised the terminology for the variables as follows: “antihypertensive drug use,” “obesity-related hospital discharge/long-term disease diagnoses,” and “antidiabetic drug use or diabetes-related hospital discharge/long-term disease diagnoses.” Additionally, we have updated the category name to “Proxies for pre-existing health conditions” to more accurately reflect the variables used.

**COMMENT 13:** Line 137-9: "Gestational week when antibiotic treatment was started was also considered for the analysis between macrolide and amoxicillin groups." Please clarify how the gestational weeks exposed were handled from a statistical or methodological perspective.

**RESPONSE**: The gestational week when treatment with macrolides or amoxicillin was initiated was included as a covariate in the propensity score model. To provide greater clarity, we have reorganized the corresponding paragraph in the Methods section (Page 9, lines 159-164).

Method - Statistical analysis

**COMMENT 14**: Line 156: Could you clarify why generalized estimating equations were used? Is it to consider any correlation due to more than one delivery episode in the same woman?

**RESPONSE**: Generalized estimating equations were used to account for the correlation between multiple pregnancies within the same woman. We have added this information “To account for the correlation between multiple pregnancies in the same woman, generalized estimating equations were applied.” in the Methods section (Page 9, lines 185).

**COMMENT 15**: Line 157-8: Could you explain more about the sentence "No adjustments were made for multiple testing."?

**RESPONSE**: In this study, we assessed the risk of 42 different individual major congenital malformations (MCMs) associated with macrolide exposure during the first trimester. However, we did not report p-values, nor did we apply adjustments for multiple testing. For a more detailed explanation of this approach, we refer the reviewer to our response to comment #1 from the Academic Editor.

“To avoid overlooking potential safety signals that may warrant further investigation, no adjustments were made for multiple comparisons. Instead, we opted to interpret the results based on the association strength, the confidence interval width, the consistency in the results of sensitivity analyses, and the null findings in the negative control analysis. This practice has been adopted in previous studies [22,23].” (Methods section, page 11, lines 217-221)

Method - Sensitivity and supplementary analysis

**COMMENT 16**: Line 165-7: Could you clarify the rationale of the first sensitivity analysis? Is it to exclude the possible influence of other ingredients and purely look at the effect of the specific ingredient?

**RESPONSE**: The first sensitivity analysis mainly aimed to control for potential confounding factors related to infection severity, as more severe infections may lead to different antibiotic treatments. Additionally, pregnant women prescribed macrolides may have a history of penicillin allergy, as indicated by previous prescriptions. This analysis helps to reduce the impact of these factors. We have clarified the rationale of the first sensitivity analysis in the Methods section.

“First, we restricted our analysis to pregnancies exposed to a single antibiotic agent (monotherapy) during the first trimester to address potential confounding by infection severity.” (Methods section, page 10, lines 193-194)

**COMMENT 17**: Line 177-9: How accurate is the data on maternal folic acid supplementation in the SNDS? I am curious because supplements are available privately or as over-the-counter drugs and often are not recorded in claims data.

**RESPONSE**: As noted in response to comment #16 from Reviewer 2, since folic acid supplementation is often available over the counter, its use cannot be completely captured in the reimbursement data. We then revised the term in the manuscript to “reimbursed folic acid supplementation” to reflect the variable more accurately. However, we have retained the stratified analysis to give additional insights, as suggested by the Academic Editor. In this analysis, among pregnancies having reimbursed folic acid supplementation (i.e. pregnancies for whom it is certain that folic acid was dispensed), the association with spina bifida was attenuated towards the null. In this subgroup, it is possible that some pregnant women had taken over-the-counter folic acid; however, there is no argument to think that the use of over-the-counter folic acid was different between the two exposure groups (macrolides and amoxicillin). This could lead to a non-differential misclassification bias, resulting in a loss of statistical power and a bias toward the null hypothesis. Although this is a limitation in our study, we thought it would be interesting to present the results of this analysis. In fact, if acid folic supplementation during the first trimester of pregnancy among women exposed to macrolides no longer shows an association with spina bifida, it is important to present it, as it may help reinforce the need to receive folic acid during the first trimester.

“Given the limitations in accurately capturing certain variables in the SNDS, the results from subgroup analysis among pregnancies with reimbursed folic acid and from the stratified post-hoc analyses should be interpreted cautiously.” (Discussion section, page 17, lines 363-365)

Results/Discussion

**COMMENT 20**: Line 199-201 & Table 1: "Parity" refers to the number of times a woman has given birth to a fetus with >20 or 24 weeks of gestational age, regardless of whether the infant was born alive or stillborn. In Table 1, does the "Parity" mean that the first birth since 2010? If then, because of left censoring in the data, I don't think the variable is actual nulliparity (that is, a woman who has never given birth before 2010) and the variable would be not really meaningful.

**RESPONSE:** We thank the reviewer for this remark. In our study, "parity" was defined as the number of live births recorded since 2010. We agree that, due to left censoring in the data, this variable does not accurately reflect true nulliparity and is therefore not meaningful. As a result, we have removed this variable from the propensity score model and revised the manuscript accordingly. This adjustment had minimal impact on the results, and the interpretation of the findings remained unchanged.

**COMMENT 21**: Table 1: In France, are all assisted reproduction (oocyte pick-up, embryo transfer, and artificial insemination) reimbursed? Would this variable have a low probability of misclassification?

**RESPONSE**: All assisted reproduction technologies, including artificial insemination, oocyte pick-up, and embryo transfer, are fully covered by French health insurance after medical validation and up to the maternal age of 43 years. Given that, the likelihood of misclassification for this variable is minimal. This information has been added to the variable definition in Table S4 (Supporting Information).

**COMMENT 22**: Can you assess any dose or duration-response relationship if information on the dose or duration of drug prescription is available?

**RESPONSE**: In this study, we did not assess the dose-effect relationship between macrolide exposure and risk of malformations as we were able to determine the exact number of tablets dispensed in the SNDS and the prescribed doses. Furthermore, we did not know the adherence of pregnant women to antibiotic treatments. However, most pregnant women (94.3%) in our study filled only one prescription for the macrolides during the first trimester (S5 Table)). We have now discussed this limitation in the Discussion section

“Fifth, we did not evaluate any dose-effect relationship as we were unable to determine the prescribed doses, the exact number of dispensed tablets, and the adherence of pregnant women to antibiotic treatments.” (Discussion section, page 16, lines 365-367)

**COMMENT 23**: Figure 1: The figure is very long, and it is not easy to directly identify the organ system associated with each outcome. I suggest that the outcome should be first classified by the organ system like Table 2, and then sorted by the most common to the least common MCMs in the organ system.

**RESPONSE**: We thank the reviewer for this suggestion. Figure 1 has been classified by the organ system and then sorted by the most common to the least common MCMs in each organ system.

COMMENT 24: Figure 2: The figure focuses on the upper limit of 95% CI, but I suggest presenting both lower and upper limits because some estimates should have very wide CIs, which means that the risk can be observed by chance. This figure is good, but it is difficult to the statistical significance.

RESPONSE: In response to the comment #23 from Reviewer 2, we have moved Figure 2 to the Supporting Information in the table format (S9 Table). Additionally, we have included the 95% confidence intervals for each result to enhance interpretability and allow readers to draw more informed conclusions.

**COMMENT 25**: Line 293: Is there any reason to refer only to Andersson et al. in this sentence? Is this study the largest existing prior study?

**RESPONSE**: Yes, the study by Andersson et al. (2021) is the largest existing prior study. The sentence has been revised accordingly: “The strengths of this study include its large, unselected cohort of pregnancies based on a national healthcare database with the number of pregnancies exposed to macrolides more than 10 times higher than in the largest prior study by Andersson et al. (2021).” (Discussion section, page 16, line 335)

**References**

1.	Huybrechts KF, Straub L, Karlsson P, Pazzagli L, Furu K, Gissler M, et al. Association of In Utero Antipsychotic Medication Exposure With Risk of Congenital Malformations in Nordic Countries and the US. JAMA Psychiatry. 2023;80: 156. doi:10.1001/jamapsychiatry.2022.4109

2.	Patorno E, Hernandez-Diaz S, Huybrechts KF, Desai RJ, Cohen JM, Mogun H, et al. Gabapentin in pregnancy and the risk of adverse neonatal and maternal outcomes: A population-based cohort study nested in the US Medicaid Analytic eXtract dataset. Stock SJ, editor. PLoS Med. 2020;17: e1003322. doi:10.1371/journal.pmed.1003322

3.	Persaud TVN, Moore KL. Causes and Prenatal Diagnosis of Congenital Abnormalities. JOGN Nursing. 1974;3: 50–55. doi:10.1111/j.1552-6909.1974.tb01236.x

4.	Carls A, Jedamzik J, Witt L, Hohmann N, Burhenne J, Mikus G. Systemic exposure of topical erythromycin in comparison to oral administration and the effect on cytochrome P450 3A4 activity. Brit J Clinical Pharma. 2014;78: 1433–1440. doi:10.1111/bcp.12497

5.	Ishimatsu Y, Kadota J-I, Iwashita T, Nagata T, Ishii H, Shikuwa C, et al. Macrolide antibiotics induce apoptosis of human peripheral lymphocytes in vitro. International Journal of Antimicrobial Agents. 2004;24: 247–253. doi:10.1016/j.ijantimicag.2004.03.022

6.	Fuchs F, Senat M-V, Rey E, Balayla J, Chaillet N, Bouyer J, et al. Impact of maternal obesity on the incidence of pregnancy complications in France and Canada. Sci Rep. 2017;7: 10859. doi:10.1038/s41598-017-11432-5

7.	Marty L, Miranda S, Weill A, Dray-Spira R. Prévalence des malformations congénitales majeures en France entre 2010 et 2021 : estimations parmi les neuf millions d’enfants et fœtus du registre EPI-MERES. Journal of Epidemiology and Population Health. 2024;72: 202373. doi:10.1016/j.jeph.2024.202373
