# Supplementary material for: First-trimester exposure to macrolides and risk of major congenital malformations compared with amoxicillin: A French nationwide cohort study
Source: PLoS Med. 2025 Apr 15;22(4):e1004576. doi: 10.1371/journal.pmed.1004576 (PMC12021278; doi:10.1371/journal.pmed.1004576)
Supplement: Attachment — Submitted filename: Response letter_R2.docx [file pmed.1004576.s026.docx]

**Requests from the Plos Medicine’s academic editor:**

**COMMENT 1**: He asked that the authors include the analysis that was adjusted for multiple comparisons more formally in the Methods section and discuss this in the Results.

**RESPONSE**: We thank the Academic Editor for the suggestion. We have added the adjustment for multiple comparisons in the Methods section as a post-hoc analysis. Specifically, we applied the Benjamini-Hochberg (BH) method with a 5% false discovery rate to correct for the occurrence of false positives. The BH method uses sequential modified Bonferroni correction for multiple hypothesis testing [1]. This method controls the false discovery rate, making it less stringent than the Bonferroni correction, which controls the family-wise error rate.

We have added the following sentence in the Method Section (page 11, lines 221-223):

*“However, to provide transparency for readers, an additional analysis was performed using the Benjamini-Hochberg correction with a 5% false discovery rate to account for multiple comparisons in the main analysis [24].”*

In the Result Section, (page 14, lines 291-294), we have added the following sentence:

“*Finally, the p-values from 42 associations assessed in the main analysis are presented in S16 Table. After adjusting for multiple comparisons, none of the p-values remained statistically significant.”*

Details of the results are presented in the Supporting Information (S16 Table).

**S16 Table**. Post-hoc analysis – Statistical significance of results from the main analyses with the Benjamini-Hochberg (BH) procedure (5% false discovery rate)

| **Individual MCMs** | **P-value** | **Rank** | **BH threshold** | **Significance** |
| --- | --- | --- | --- | --- |
| Spina Bifida | 0.00318 | 1 | 0.00119 | Not significant |
| Syndactyly | 0.02789 | 2 | 0.00238 | Not significant |
| Hydrocephaly | 0.06240 | 3 | 0.00357 | Not significant |
| Hip dislocation | 0.10329 | 4 | 0.00476 | Not significant |
| Cleft palate | 0.11256 | 5 | 0.00595 | Not significant |
| Cleft lip with and without cleft palate | 0.15806 | 6 | 0.00714 | Not significant |
| Situs inversus | 0.16857 | 7 | 0.00833 | Not significant |
| Limb reduction defects | 0.16947 | 8 | 0.00952 | Not significant |
| Atrioventricular septal defect | 0.17746 | 9 | 0.01071 | Not significant |
| D-TGA | 0.19174 | 10 | 0.01190 | Not significant |
| Atrial septal defect | 0.20645 | 11 | 0.01310 | Not significant |
| Horseshoe kidney | 0.21271 | 12 | 0.01429 | Not significant |
| Gastroschisis | 0.27951 | 13 | 0.01548 | Not significant |
| Hydronephrosis | 0.28122 | 14 | 0.01667 | Not significant |
| Double outlet right ventricle | 0.29153 | 15 | 0.01786 | Not significant |
| Congenital pulmonary valve | 0.29600 | 16 | 0.01905 | Not significant |
| Ano-rectal atresia | 0.30304 | 17 | 0.02024 | Not significant |
| Diaphragmatic hernia | 0.30531 | 18 | 0.02143 | Not significant |
| Agenesis of the corpus callosum | 0.34069 | 19 | 0.02262 | Not significant |
| Coarctation of aorta | 0.34767 | 20 | 0.02381 | Not significant |
| Laterality anomalies | 0.35024 | 21 | 0.02500 | Not significant |
| Pulmonary valve atresia | 0.35635 | 22 | 0.02619 | Not significant |
| Aortic valve atresia/stenosis | 0.38391 | 23 | 0.02738 | Not significant |
| Craniosynostose | 0.38956 | 24 | 0.02857 | Not significant |
| Hypoplastic left heart | 0.42034 | 25 | 0.02976 | Not significant |
| Posterior urethral valve | 0.48211 | 26 | 0.03095 | Not significant |
| PDA as only CHD in term infants | 0.55250 | 27 | 0.03214 | Not significant |
| Hirschrung's disease | 0.60947 | 28 | 0.03333 | Not significant |
| Atresia or stenosis of intestine | 0.62903 | 29 | 0.03452 | Not significant |
| Unilateral Renal Agenesis | 0.63995 | 30 | 0.03571 | Not significant |
| Polydactyly | 0.65687 | 31 | 0.03690 | Not significant |
| Tetralogy of Fallot | 0.81849 | 32 | 0.03810 | Not significant |
| Oesophageal atresia | 0.84340 | 33 | 0.03929 | Not significant |
| Vascular disruption anomalies | 0.84436 | 34 | 0.04048 | Not significant |
| Club foot | 0.84732 | 35 | 0.04167 | Not significant |
| Hypospadias | 0.86456 | 36 | 0.04286 | Not significant |
| Ventricular septal defect | 0.89212 | 37 | 0.04405 | Not significant |
| Omphalocele | 0.89467 | 38 | 0.04524 | Not significant |
| Severe microcephaly | 0.91248 | 39 | 0.04643 | Not significant |
| Anomalies of intestinal fixation | 0.93444 | 40 | 0.04762 | Not significant |
| Renal Dysplasia | 0.96615 | 41 | 0.04881 | Not significant |
| Congenital cataract | 0.99638 | 42 | 0.05000 | Not significant |

**COMMENT 2**: He also asked that the authors incorporate this analysis into the authors’ interpretation of the results in the Discussion (not just in the limitations section).

**RESPONSE**: We thank the Academic Editor for the comment. In response, we have now added the interpretation of the results for the adjustment for multiple comparisons in the Discussion Section of the manuscript (page 16, lines 330-343).

*“Given the heterogeneity of pathophysiological mechanisms underlying multiple MCMs [37], it is crucial to investigate MCMs individually to avoid missing safety signals. As numerous MCMs were assessed concurrently in our study, concerns over multiple comparisons might arise. When applying the Benjamini-Hochberg correction, it was almost expected that none of the associations in the main analysis remained statistically significant considering the large number of analyses conducted and the limited number of macrolide-exposed events. Although we cannot entirely rule out the possibility that the increased risk for spina bifida and syndactyly was observed by chance, the elevated estimate points persisted across individual macrolide and sensitivity analyses. Additionally, the findings from the negative control analysis were, as expected, null. Furthermore, an increased risk of syndactyly and neural tube defects associated with macrolide exposure during the first trimester was observed in some previous studies [10,31,33]. Thus, these findings should be considered as safety signals warranting further investigation.”*

Additional references:

Toufaily MH, Westgate M, Lin AE, Holmes LB. Causes of Congenital Malformations. Birth Defects Research. 2018;110: 87–91. doi:10.1002/bdr2.1105

In the Limitation Section of the manuscript, we have retained the following sentence:

*“Although we acknowledge the possibility of false-positive findings, the p-value use could lead to missing potential safety signals [38]. Therefore, our interpretation was based on the association strength, the confidence interval width, the consistency of the results in sensitivity analyses, and the null negative control association. Given the exploratory nature of our study, we need to keep the observed associations under surveillance and reevaluate them in future research. Replicating our findings in different populations and studies is essential to establish more robust evidence.”*

**Comments from the Reviewers:**

**Reviewer 1:**

**COMMENT**: Upon this second review, I'm pleased to note that the authors have effectively addressed all the concerns I raised in my initial review. The authors have carefully revised the manuscript, providing clear explanations and relevant data. Therefore, I recommend this manuscript for publication in the journal.

**RESPONSE**: We thank the reviewer for the positive feedback.

**Reviewer 2:**

**COMMENT 1**: Were all covaries listed in the “covariates” heading included in the models? It is unclear how this is currently worded with “considered”.

**RESPONSE**: All covariates listed in the “Covariates” heading were included in the models. We have now replaced “considered” with “included”. (Methods section, page 8, lines 160)

**COMMENT 2**: How was gestational age at exposure handled? This is an important difference that was not clear earlier. It is plausible that the earlier exposure window for macrolides compared with amoxicillin (approx. 1 week earlier) may influence the outcome (the greatest risk of neural tube defects occurring approximately weeks 3-4).

**RESPONSE**: In our study, the gestational age at exposure was included as a covariate in the propensity score model.

“*The gestational week at which treatment with macrolides or amoxicillin was initiated was also included*.” (Methods section, page 9, line 164)

“*We estimated a separate propensity score for each macrolide exposure group using a logistic regression model that included all covariates listed above*.” (Methods section, page 9, lines 172-174)

**COMMENT 3**: The authors refer to the Benjamini-Hochberg correction, although appropriate given the number of hypotheses tested, this is not seen in the manuscript (only discussion). Details of this should be included, including what p-value was set and the results of this (as a supplement).

**RESPONSE**: We thank Reviewer 2 for this suggestion, which is aligned with Comment 1 of the Academic Editor. We thank the Academic Editor for the suggestion. We have added the adjustment for multiple comparisons in the Methods section as a post-hoc analysis. Specifically, we applied the Benjamini-Hochberg (BH) method with a 5% false discovery rate to correct for the occurrence of false positives. The BH method uses sequential modified Bonferroni correction for multiple hypothesis testing [1]. This method controls the false discovery rate, making it less stringent than the Bonferroni correction, which controls the family-wise error rate.

We have added the following sentence in the Method Section (page 11, lines 221-223):

*“However, to provide transparency for readers, an additional analysis was performed using the Benjamini-Hochberg correction with a 5% false discovery rate to account for multiple comparisons in the main analysis [24].”*

In the Result Section, (page 14, lines 291-294), we have added the following sentence:

“*Finally, the p-values from 42 associations assessed in the main analysis are presented in S16 Table. After adjusting for multiple comparisons, none of the p-values remained statistically significant.”*

Details of the results are presented in the Supporting Information (S16 Table).

**COMMENT 4**: The potential limitation of misclassification of folic acid use due to only prescriptions captured needs to be further highlighted.

**RESPONSE**: We thank the reviewer for the comment. In our study, we defined folic acid supplementation as “reimbursed folic acid supplementation” to clarify that the folic acid variable relied solely on prescription data, which may not fully capture the actual usage. Although the variable could not fully capture the actual usage, there is no reason to think that the use of over-the-counter folic acid was different between the macrolide and amoxicillin exposure groups. Therefore, this bias source is likely to have a small impact on our result interpretation.

In the analysis stratified by folic acid use, the non-differential misclassification is unlikely to alter our result interpretation. In the stratum of pregnancies without reimbursed folic acid supplementation, the non-differential misclassification could reduce statistical power and bias results toward the null. Despite this potential bias, the relative risk for spina bifida among women exposed to macrolides compared with the amoxicillin group remained elevated aRR=2.39 [1.52;3.77]. Conversely, in the stratum of pregnancies having reimbursed folic acid supplementation (i.e., pregnancies for whom folic acid was certainly dispensed), the association with spina bifida was attenuated towards the null. If this association is true, it may help reinforce the need to receive folic acid during early pregnancy, so it is important to present this result.

In response, we have now added the following sentence in the Limitation Section (lines 373-375):

“*Although non-reimbursed folic acid supplements are not captured in the SNDS, their use is probably not distributed differently between the macrolide and amoxicillin exposure groups. The non-differential misclassification bias might reduce the statistical power but is unlikely to affect markedly our interpretation.”*

**Reviewer 3:**

**COMMENT:** I sincerely appreciate the dedicated efforts of the authors in addressing my comments and the thoughtful guidance provided by the editors. All my comments have been thoroughly addressed, and I am grateful for the opportunity to contribute to this excellent work.

**RESPONSE**: We thank the reviewer for the positive feedback.

**Editorial requests:**

**REQUEST 1:** Please ensure that the paper adheres to the PLOS Data Availability Policy (see http://journals.plos.org/plosmedicine/s/data-availability). For data residing with a third party, authors are required to provide instructions with contact information (web or email address) for obtaining the data. Please note that a study author cannot be the contact person for the data.

**RESPONSE**: We have added contact information in Data sharing statement section (lines 395-400):

“*However, any individual or organization (public or private; for-profit or non-profit), can request access to anonymized SNDS data to conduct research or evaluations of public interest. upon authorization from the National Commission for Data Protection and Liberties (CNIL), via the French Health Data Hub (https://www.health-data-hub.fr/page/faq-english under section “About the data available through the Health Data Hub”, or by contacting* [*hdh@health-data-hub.fr)*](mailto:hdh@health-data-hub.fr))*.”*

**REQUEST 2**: It appears that the STROBE checklist was not uploaded in this revision. Could the authors make sure this is uploaded for the next revision please? Please also ensure that the checklist has section and paragraph numbers in the right hand column, rather than page numbers.

**RESPONSE**: The STROBE Checklist has now been uploaded.

**REQUEST 3**: Thank you for providing an Author Summary. The purpose of this section is to make your findings accessible to a wider audience. As such, some terms, though they may seem obvious to a specialist, may need some explanation. For example, please explain that macrolides are a class of antibiotics (in the first sentence). It may also be beneficial to (very briefly) explain why macrolides were compared to amoxicillin.

**RESPONSE**: We have updated the corresponding sentences in the Author Summary.

“*Macrolides are a frequently prescribed class of antibiotics during pregnancy.”* (line 53)

“*This nationwide cohort study compared the risk of MCMs overall and 42 individual MCMs among over 140,000 pregnancies exposed to macrolides to over 592,000 pregnancies exposed to amoxicillin (a commonly used antibiotic during pregnancy with a well-established safety profile) during the first trimester*.” (lines 58-61)

**References for the response letter**

1.	Benjamini Y, Hochberg Y. Controlling the False Discovery Rate: A Practical and Powerful Approach to Multiple Testing. Journal of the Royal Statistical Society: Series B (Methodological). 1995;57: 289–300. doi:10.1111/j.2517-6161.1995.tb02031.x
