# Supplementary material for: First-trimester exposure to macrolides and risk of major congenital malformations compared with amoxicillin: A French nationwide cohort study
Source: PLoS Med. 2025 Apr 15;22(4):e1004576. doi: 10.1371/journal.pmed.1004576 (PMC12021278; doi:10.1371/journal.pmed.1004576)
Supplement: Attachment — Submitted filename: PointByPointResponse 15 Mar.docx [file pmed.1004576.s027.docx]

**Requests from Editors:**

**GENERAL EDITORIAL REQUESTS**

* Please confirm that your title complies with PLOS Medicine's style. Your title must be nondeclarative and not a question. It should begin with main concept if possible. "Effect of" or "risk of" should be used only if causality can be inferred, i.e., for an RCT. Please place the study design ("A randomized controlled trial," "A retrospective study," "A modelling study," etc.) in the subtitle (ie, after a colon).

RESPONSE: We confirm that our study title complies with PLOS Medicine’s style.

* Please confirm that your abstract complies with our requirements, including providing all the information relevant to this study type https://journals.plos.org/plosmedicine/s/submission-guidelines#loc-abstract19.

RESPONSE: We confirm that our abstract complies with your requirements.

* Please ensure that all abbreviations are defined at first use throughout the text.

RESPONSE: We confirm that all abbreviations were defined at first use throughout the text. We have now added an Abbreviations section on page 19.

* Please use a comma to separate the confidence intervals as using a 'dash' can be confused with a minus sign.

RESPONSE: We have now used “to” instead of a ‘dash’ to separate the confidence intervals.

FUNDING STATEMENT

* The funding statement should include: specific grant numbers, initials of authors who received each award, URLs to sponsors’ websites. Also, please state whether any sponsors or funders (other than the named authors) played any role in study design, data collection and analysis, the decision to publish, or preparation of the manuscript. If they had no role in the research, include this sentence: “The funders had no role in study design, data collection and analysis, decision to publish, or preparation of the manuscript.”

RESPONSE: We declared that no funding had been provided for this study.

COMPETING INTERESTS STATEMENT

* All authors must declare their relevant competing interests per the PLOS policy, which can be seen here: https://journals.plos.org/plosmedicine/s/competing-interests For authors with ties to industry, please indicate whether any of the interests has a financial stake in the results of the current study.

RESPONSE: We declared that all authors have no competing interests.

OBSERVATIONAL, COHORT, CROSS-SECTIONAL, AND CASE CONTROL STUDIES

* Did your study have a prospective protocol or analysis plan? Please state this (either way) early in the Methods section.

a) If a prospective analysis plan (from your funding proposal, IRB or other ethics committee submission, study protocol, or other planning document written before analyzing the data) was used in designing the study, please include the relevant prospectively written document with your revised manuscript as a Supporting Information file to be published alongside your study, and cite it in the Methods section. A legend for this file should be included at the end of your manuscript.

b) If no such document exists, please make sure that the Methods section transparently describes when analyses were planned, and when/why any data-driven changes to analyses took place.

c) In either case, changes in the analysis-- including those made in response to peer review comments-- should be identified as such in the Methods section of the paper, with rationale."

RESPONSE: The study protocol was pre-specified and approved by the scientific committee of the EPI-PHARE team before conducting this study. While the study protocol was not registered in a publicly accessible registry, it was documented in the EPI-PHARE repository. We have now provided a summary of the study protocol in the S1 Protocol of the Supporting Information with the citation to the list of legends of the supporting information files.

* Your study is observational and therefore causality or calculation of risk cannot be inferred. Please remove language that implies causality and refer to associations instead.

RESPONSE: Throughout the manuscript, we have carefully used language that refers to associations (e.g., “...associated with an increased risk of…”).

**Comments from Reviewers:**

Reviewer #2: I thank the authors for their considered response and addressing these queries.

RESPONSE: We thank the reviewer for the positive feedback.

**Technical requests:**

**Journal Requirements**

1) The file inventory includes files for Figures 1A,B,C,D.We would recommend either combining these into a single figure file with separate internal panels, or renumbering them as individual figures , as we are not able to publish multiple components of a single figure as separate files.

NOTE: Please ensure that all included figures are referenced to ensure that your paper is fully reviewed.

RESPONSE: We have now renumbered 4 panels of the Figure 1 as individual figures – Figures 1, 2, 3, and 4. Accordingly, we have updated the manuscript and the corresponding figure legends.

2) We have noticed that you have uploaded " S1_Checklist.doc" file as supporting information but you have not included it in the list of legends. Please add a citation of this SI file into the list of legends of your supporting information files.

RESPONSE: We have now added the citation S1 Checklist to the list of legends of the supporting information files.
